# Extension of Tian Shan along a nascent shear zone

**DOI:** 10.1038/s41467-026-74965-2

**Published:** 2026-07-22

**Authors:** Qi Ou, John R. Elliott, Yasser Maghsoudi, Chris Rollins, Milan Lazecky, Tim J. Wright

**Affiliations:** 1 COMET, School of Earth, Environment and Sustainability, University of Leeds, Leeds, United Kingdom; 2 COMET, School of GeoSciences, University of Edinburgh, Edinburgh, United Kingdom; 3 COMET, Department of Earth and Environmental Sciences, University of Exeter, Penryn Campus, Penryn, TR10 9FE Cornwall United Kingdom; 4 Earth Sciences New Zealand, 1 Fairway Drive, Lower Hutt, 5011 New Zealand

**Keywords:** Tectonics, Hydrogeology, Cryospheric science

## Abstract

Our understanding of the dynamics of mountain belt growth is hampered by the lack of high-resolution kinematic observations spanning entire orogenic belts. This is particularly the case for the structurally complex and nascent Tian Shan plateau. Here we use 8 years of Sentinel-1 data across 2 million square kilometres of the Tian Shan to show that the mountain range is extending along its strike, predominantly by rotating along a northeast-trending distributed shear zone. This zone is conjugate to the range strike but aligned with fast axes of shear-wave splitting measurements and a band of strike-slip earthquakes. We interpret this broad zone of shear as resulting from the clockwise rotation of the indenting Tarim Basin, which drives the northeast-to-east motion of the part of the Tian Shan southeast of the cryptic shear zone. Conjugate strike-slip components on numerous basin-bounding faults within the Tian Shan likely facilitate this escape. The present-day vertical deformation of Tian Shan results from a mix of tectonic, climatic, and anthropogenic forcings, with uplift of the highest peak facilitated by thrust along a south-dipping Nalati fault that could be promoted by deglaciation.

## Abstract (web summary)

In this study, the authors identify a northeast-trending distributed shear zone that is slicing the Tian Shan mountain into two halves. The plate boundary is growing inland with India’s continued indentation into Eurasia.

## Introduction

The Tian Shan orogenic belt, reactivated in the late Cenozoic as a far-field response to the ongoing Indo-Eurasia collision^1^, is a unique example of a young plateau at an intermediate stage of mountain building. Sustained shortening and thickening of the crust has created the highest and longest mountain belt in Central Asia, which hosts some of the largest earthquakes on the continent^2^. As one of the best illustrations of an intercontinental mountain belt, this region provides an ideal case of using the kinematics of an entire range for understanding the dynamics of continental collision and mountain growth^3^.

**Fig. 1:**
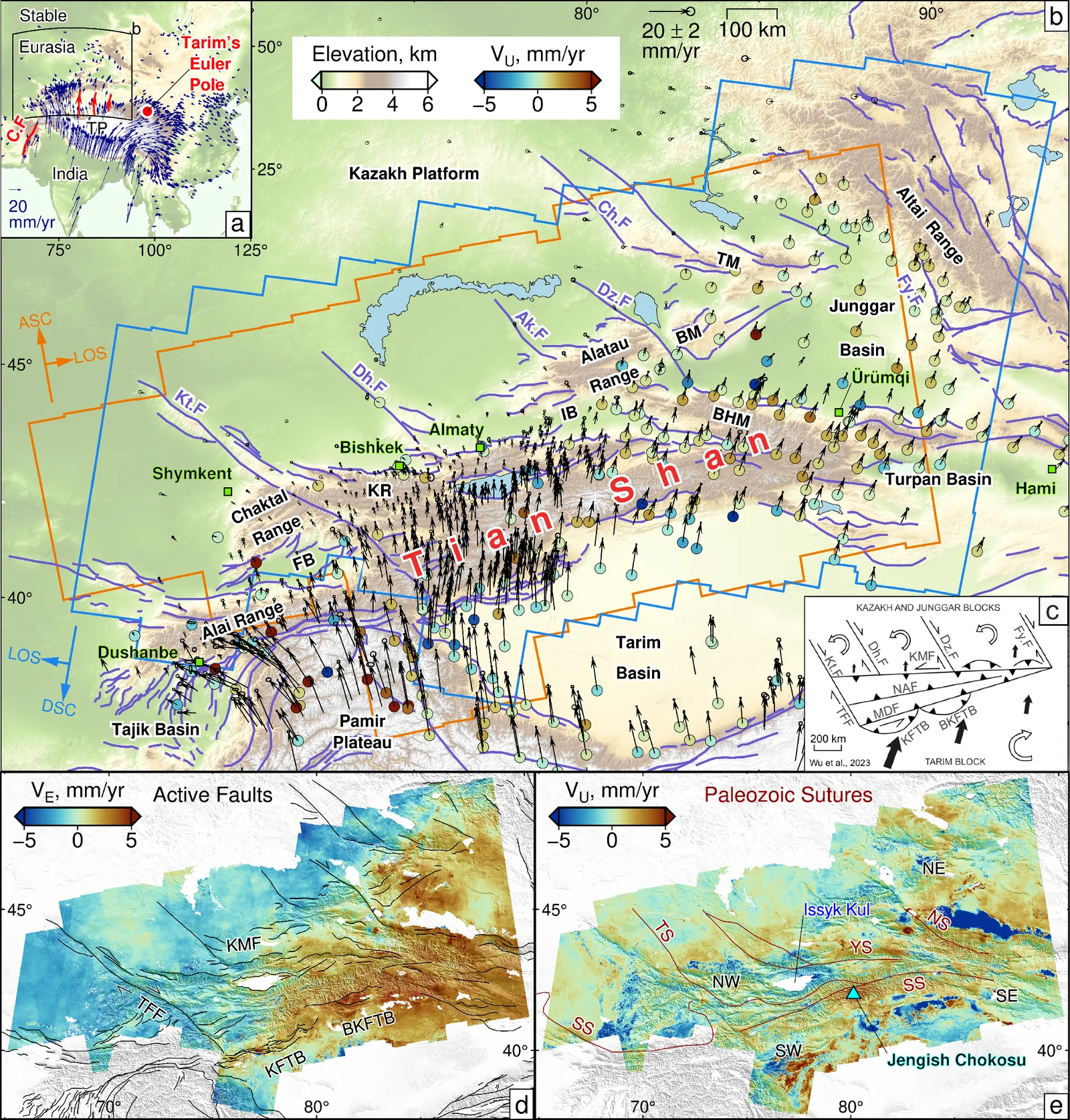
Tectonic overview of the Tian Shan with GNSS and InSAR velocities. **a** Location of the intracontinental mountain belt of the Tian Shan, Central Asia, in the Indo-Eurasia collision zone with dark blue arrows showing horizontal Global Navigation Satellite System (GNSS) velocities with ellipses showing 1-sigma uncertainties^59^ in a Fixed-Eurasia reference frame. C.F Chaman Fault. Red dot indicates Tarim’s Euler Pole^45^. Red arrows indicate clockwise rotation of the Tarim Basin. Digital Elevation Model is from the Shuttle Radar Topography Mission^177^. **b** Kinematic overview of the Tian Shan, with black arrows and coloured dots showing horizontal and vertical GNSS velocities we compiled from 22 published studies (Section “Velocity mosaicking, decomposition”). Orange (ascending) and blue (descending) polygons show extents of Sentinel-1 data processed in this study. FB Fergana Basin, IB Ili Basin, KR Kyrgyz Range, BM Baerluke Mountain, TM Taerbahatai Mountain, BHM Borohoro Mountain. Cities are labelled in green. **c** Tectonic model adapted from Wu et al.^16^. TFF Talas-Ferghana Fault, KMF Kemin Fault, NAF Nalati Fault, MDF Maidan Fault. Northwest-trending platform-cutting right-lateral strike-slip faults are labelled as Kt.F Karatau-Talas-Fergana Fault, Dh.F Dzhalair-Naiman Fault, Ak.F Aktas Fault, Dz.F Dzhungarian fault, and Ch.F Chingiz fault^22,119^. Along the southern range front are Kalpin Fold-and-Thrust Belt (KFTB) and Baicheng-Kuche Fold-and-Thrust Belt (BKFTB). Purple lines in (**b**) and black lines in (**d**) show active faults in the Global Earthquake Model (GEM) Active Faults database^178^, which do not include NAF or MDF. **d** East and **e** vertical velocities derived from InSAR and GNSS data in this study. **e** Maroon lines show Paleozoic sutures^12^: NS Northern Tian Shan Suture, YS Yili Suture, TS Terskey Suture, SS Southern Tian Shan Suture. Cyan triangle represents the highest peak, Jengish Chokosu (7439 m).

Bounded between the rigid Tarim Basin in the south and the crystalline Kazakh Platform and Junggar Basin in the north (Fig. 1), the Tian Shan absorbs approximately half^4^ (to nearly two-thirds in western Tian Shan^5^), of the present-day Indo-Eurasia oblique convergence rate. The present deformation in this region, especially in western and central Tian Shan is dominated by the range-perpendicular horizontal shortening and can be approximated as a one-dimensional deformation problem^6^. However, Tian Shan’s complex tectonic history has resulted in significant structural heterogeneity and kinematic variability across the mountain belt^7–9^. Four sub-parallel range-cutting NW-trending suture zones (Fig. 1e) were created by Late Proterozoic island arc accretion, Ordovician-to-Devonian back-arc rifting^10^, and early Permian closure of oceanic basins^11,12^. Early Mesozoic quiescence flattened topography, before late Mesozoic tectonics^13^ and the major Cenozoic orogenic event, from the collision of India in the far south, rejuvenated the Paleozoic structures to accommodate over 200 km of north-south shortening in western Tian Shan (~76.6^∘^E), and about 125 km of shortening in eastern Tian Shan (~85.5^∘^E)^14,15^.

The current active tectonics in the region is thought to be accommodated by a mega-flower structure within the Tian Shan^16^ (Fig. 1c, d). North-south shortening is accommodated on range-front thin-skinned Kalpin (KFTB) and Baicheng-Kuche (BKFTB) fold-and-thrust belts, range-interior thick-skinned basin-bounding faults, and basin interior folds^17,18^. East-west extension occurs through a conjugate pair of strike-slip systems between sinistral shear on the ENE-trending Kemin, Nalati and Maidan Faults within, and parallel to, the Tian Shan range, and dextral shear on the NW-trending Karatau-Talas-Fergana, Dzhalair-Naiman, Aktas, Dzhungarian, Chingiz and Fuyun strike-slip faults that facilitate anticlockwise block rotation in the Kazakh Platform north of the Tian Shan^16^. A total of over 200 km of displacement has been recorded on the NW-trending right-lateral strike-slip Talas Fergana Fault (TFF) since the Permian (Fig. 1d), signifying its important role in accommodating deformation in the Tian Shan. However, different geological^19,20^ and geodetic^5,16^ slip rate estimates on this fault have invoked contrasting models of temporally variable fault kinematics^20,21^ and of block rotation^22,23^ to explain the discrepancy.

To answer the long-standing debates about how deformation is accommodated through the foreland thrust systems, intermontane basin bounding faults, basin interior folds, inherited suture zones, and conjugate strike-slip faults in the Tian Shan^6,24,25^ requires large-scale high-resolution velocity fields. Global Navigation Satellite System (GNSS) velocities, especially in the vertical dimension, are often too sparse and sometimes inconsistent to reveal where and how fast Tian Shan is building its complex topography (Fig. 1b)^26–29^. Previous velocity mapping with Interferometric Synthetic Aperture Radar (InSAR) in Tian Shan tended to be limited in scope and/or suffered from phase decorrelation problems due to snow cover and steep terrain^30–33^.

**Fig. 2:**
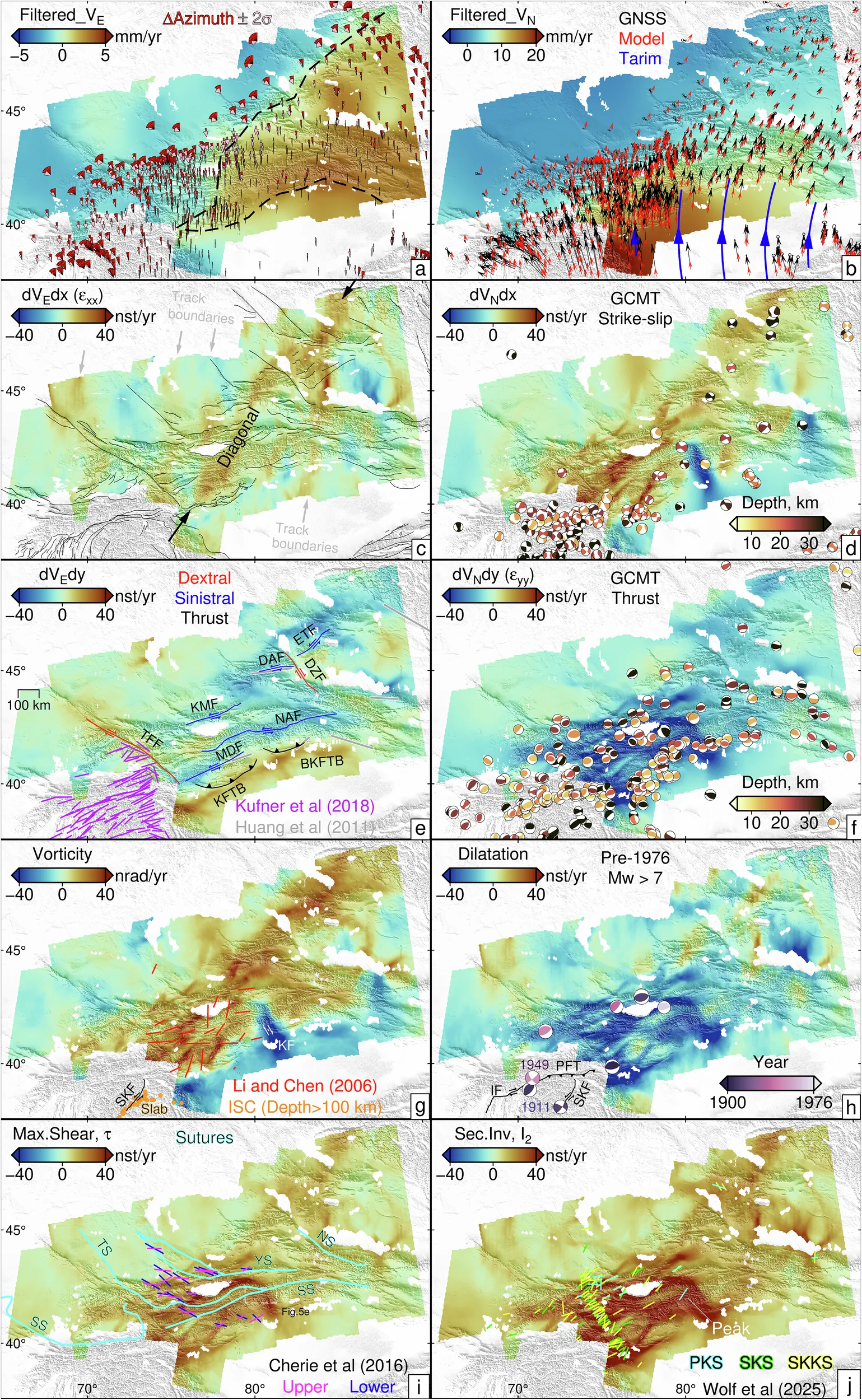
Horizontal velocities, gradients, vorticity, and strain rates with seismicity and seismic anisotropy. **a** East velocities (Fig. 1d) median-filtered with a 200 km window. Wedges represent misfits (red) with 2-sigma uncertainties (pink) between **b** GNSS vector azimuths (black arrows) and predicted azimuths (red arrows) in the sense of Tarim’s rotation (blue arrows) overlying interpolated GNSS north velocities median filtered with a 200 km window. **c**–**f** Horizontal velocity gradients in the east and south directions of the filtered east and north velocities in (**a**–**b**). **g**–**j** Vorticity (positive anti-clockwise), dilatation, maximum shear, and second invariant of strain rates derived from velocity gradients in (**c**–**f**). Fault lines in (**c**) are from GEM^178^. Blue and red lines in (**e**) show faults with sinistral and dextral component slip rates greater than 1 mm/yr from kinematic models^72^ and GNSS constraints^16^. DAF Dzhungarian-Alateau Fault, DZF Dzhungarian Fault, ETF East Tacheng Fault. Other faults are as in Fig. 1. Post-1976 focal mechanisms of (**d**) strike-slip and (**f**) thrust earthquakes from Global Centroid Moment Tensor (GCMT) catalogue^52^ are coloured by focal depths. **h** Focal mechanisms of major pre-1976 historical earthquakes are coloured by year^46–51^. Orange dots in (**g**) show epicentral locations of International Seismological Centre - Global Earthquake Model Catalogue events^179–183^ with focal depths greater than 100 km. **g**–**h** SKF Sarez Karakul Fault, KF Kalayuergun Fault, PFT Pamir Frontal Thrust, IF Ilyak Fault. Coloured lines in (**e**, **g**, **j**) show fast axes of shear-wave splitting measurements scaled by station-averaged time delay^57,62,63,65^. In (**i**), pink and blue lines show shear-wave splitting results for two-layer anisotropy^64^. Cyan lines are Paleozoic sutures^12^ (Fig. 1e).

**Fig. 3:**
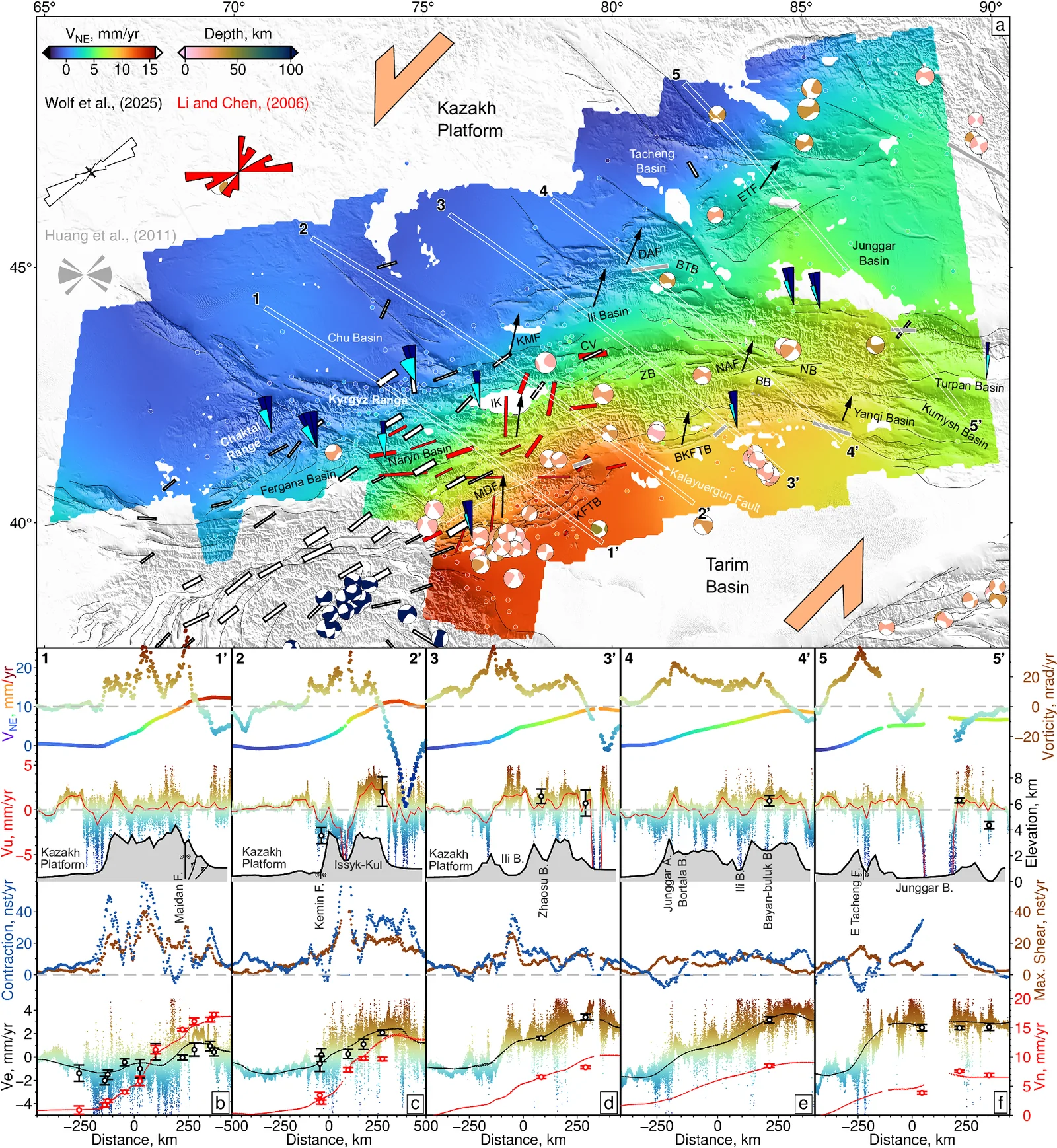
Shear-zone-parallel velocities with paleomagnetic rotation angles, stress vectors, strike-slip seismicity and anisotropy. **a** Northeast velocities combining the filtered east and north velocities (Fig. 2a, b) and at GNSS locations (coloured circles). Anisotropy results, summarised in rose diagrams, are shown in full from Huang et al.^63^ (Fig. 2e) and from Li and Chen^62^ (Fig. 2g), but reduced to 1-arcdegree block mean results weighted by time delay, plotted at mean locations, with width scaled by the number of stations in the block from Wolf et al.^65^ (Fig. 2j). Black arrows scaled by distance to Tarim’s Euler Pole^42,43^) (Fig. 1a) show azimuths in the sense of Tarim’s rotation across the southeast half of the Tian Shan. Wedges in navy blue with uncertainties in cyan show paleomagnetic rotation angles since the Miocene^66–68^. GCMT strike-slip earthquakes are shown in full within the Tian Shan, but reduced to only events with focal depths greater than 100 km in the Pamir (west of 75^∘^E and south of 40^∘^N). White lines add left-lateral strike-slip Maidan Fault (MDF)^184^ (Fig. 2e) and right-lateral strike-slip Kalayuegun Fault^135^ (Fig. 2g) to the active faults from GEM^178^ in black. NB Nalati Basin, ZB Zhaosu Basin, BB Balabuluk Basin, BTB Bortala Basin, IK Issyk-Kul. See other abbreviations in Figs. 1 and 2. **b**–**f** Ten-kilometer-wide profiles of (from top to bottom) northeast velocities (**a**), vorticity (Fig. 2g), vertical velocities (Fig. 1e), SRTM DEM median-filtered every 4 km along profile, maximum shear rates (Fig. 2i), dilatation rates (Fig. 2h), east (Fig. 1d (colour), and Fig. 2a (black)) and north (Fig. 2b) velocities from InSAR and GNSS.

**Fig. 4:**
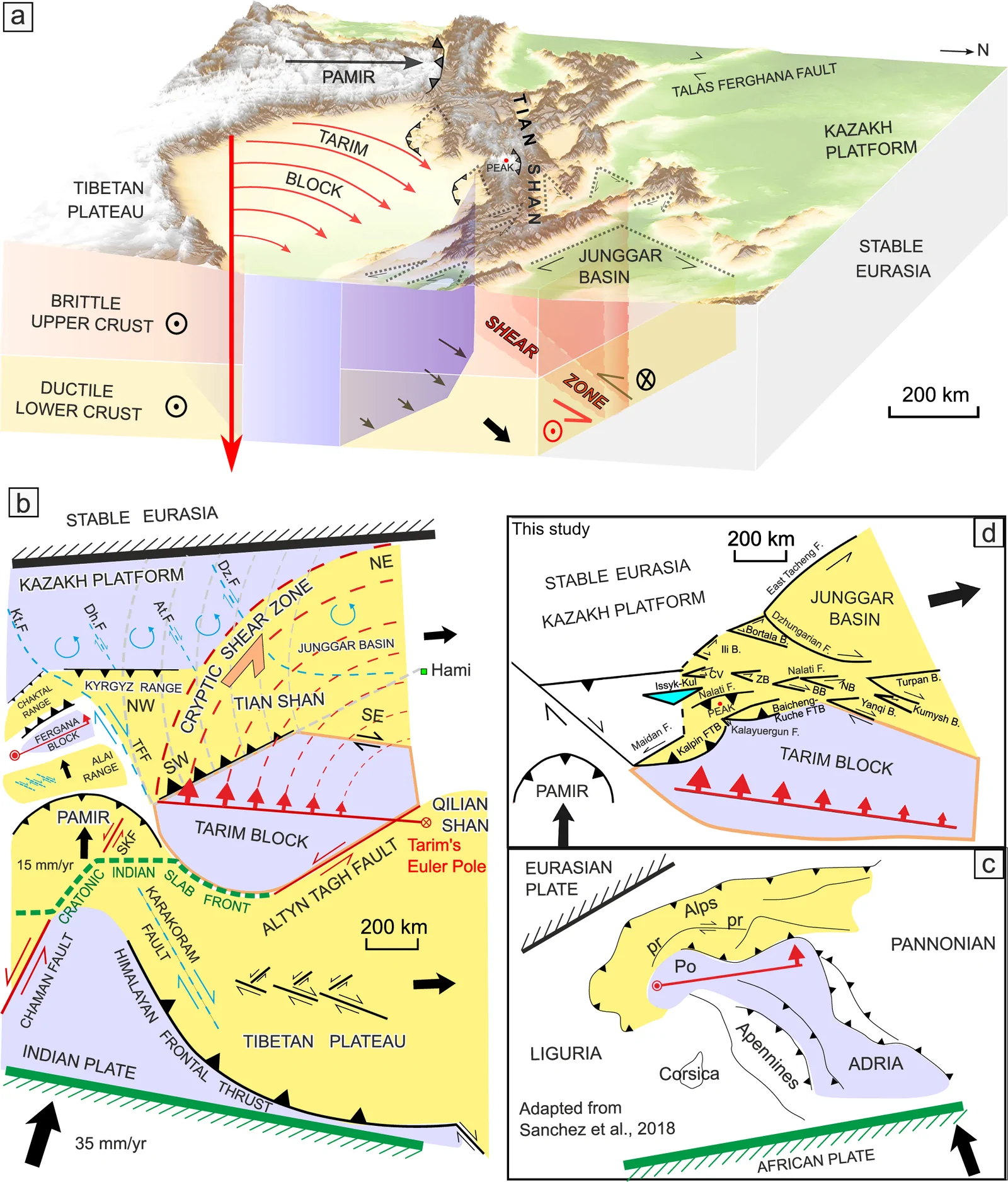
Conceptual models of tectonics of Tian Shan and the Indo-Eurasian Collision Zone. **a** Perspective view of the Tarim Basin bulldozing into the Tian Shan, driving the shear motion along a cryptic shear zone that promotes surface faulting oblique to it. **b** Cartoon showing the stress field change with the southward shift of the Euler Pole from Hami to the Qilian Shan, the tendency of the part of Tian Shan southeast of the shear zone to rotate into a tectonic regime mirroring that of the Western Tibetan Plateau, and the approximate alignment between the Chaman Fault, the northwestern limb of the cratonic Indian Slab Front^98^, Sarez Karakul Fault (SKF) and the nascent shear zone through the Tian Shan. **c** Similar tectonic regime of the Alps, with a shear zone (pr Periadriatic Line) developed through the anti-clockwise rotation of the Adriatic plate driven by the northwestward motion of the African plate (adapted from Le Breton et al.^77^). **d** Our model showing the eastward movement of the crustal material southeast of the cryptic shear zone through a series of east-opening triangular wedges at varying spatial scales. B Basin. Thrust components on the basin-bounding faults are omitted for simplicity. Half-strike-slip symbols are plotted on faults with known strike-slip components from geological and geodetic studies^16,37,99–103,127,185^. See Fig. 3a for labels on the hillshade.

**Fig. 5:**
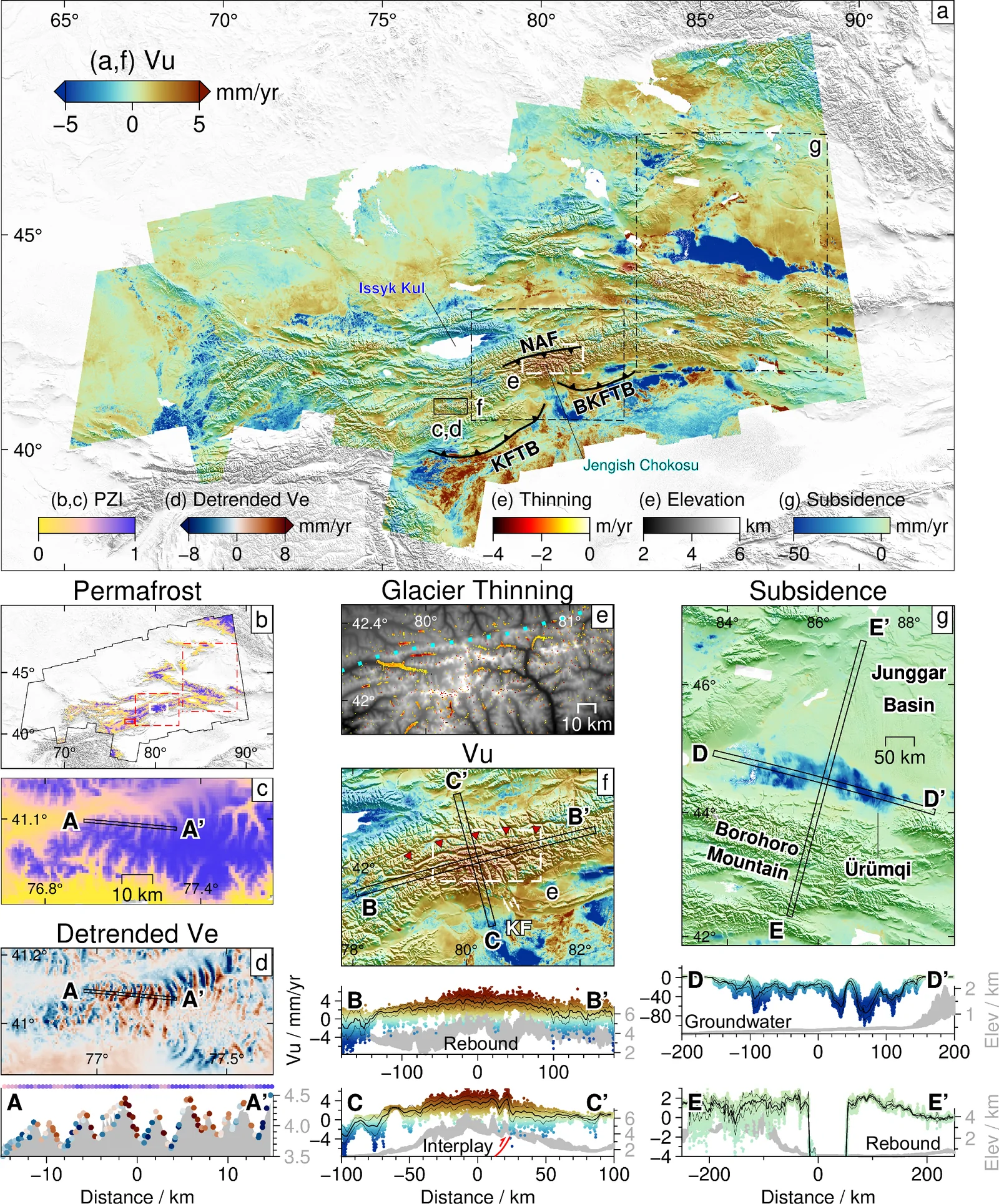
Non-tectonic velocities across the Tian Shan. **a** Vertical velocities with alternating geometries between the Nalati Fault (NAF), Kalpin Fold-and-Thrust Belt (KFTB), and Baicheng-Kuche Fold-and-Thrust Belt (BKFTB). **b** Permafrost zonation index (PZI)^120^ within the InSAR mapping area, with a zoomed-in view in (**c**). A PZI near 1 indicates that permafrost is expected nearly everywhere in a pixel. A PZI near 0 means permafrost presence is only expected under the most favourable conditions. **d** Residual InSAR Ve of the same extent as (**c**), after removing the median-filtered Ve in Fig. 2a from the unfiltered Ve in Fig. 1d. Profile A–A' shows opposite horizontal component motion in opposite down-slope directions with PZI (0.5–1) showing permafrost presence, but not the most stable (PZI = 1)^120^. **e** Glacier elevation change rates^125,186^ on top of SRTM DEM^177^. Cyan dotted line shows the Southern Tian Shan Suture^12^ (Fig. 2i), which marks a drainage divide that aligns with a step change in InSAR vertical velocities in (**f**) indicated by red triangles. KF Kalayuergun Fault. Profiles B–B' and C–C' show long-wavelength uplift centred on the highest peak in Southern Tien Shan, potentially facilitated by a south-dipping thrust fault, where the Nalati Fault was mapped but often shown as north-dipping^16,127^. **g** InSAR vertical velocity showing rapid subsidence in an agricultural field to the north of Urumqi, with profile D–D' showing subsidence reaching 80 mm/yr and long-wavelength uplift perpendicular to the field. Grey shading in profiles B–B' to E–E' show topography profiles from SRTM^177^. Thick and thin black lines on vertical velocity profiles show sliding mean and 1 standard deviation.

In this study, we process >100,000 interferograms using Sentinel-1 data and compile 673 GNSS velocities from 22 published studies to derive 500 m resolution east and vertical velocity fields (Fig. 1d, e) and 5 km-resolution vorticity and strain rate fields (Fig. 2) covering the majority (over 1.6 million km^2^) of the Tian Shan mountain belt (Section “Methods”, Supplementary Text S1–S7, Supplementary Figs. S1–S17). The east velocity gradients and vorticity fields (Fig. 2c, e, g) reveal a range-scale and range-cutting arcuate left-lateral shear zone trending northeast. Based on our velocity, vorticity and strain rate mapping, and from earthquake focal mechanisms (Fig. 2d, f, h) and previously published shear wave splitting (Fig. 2e, g, i, j) and paleomagnetic rotation measurements (Fig. 3a), we explore the possibility that the Tian Shan is in the early stage of developing a range-cutting shear zone centered along a NE-trending diagonal (Figs. 2a, b, 3, and 4). We show that this shear zone is driven by the clockwise rotation of the Tarim Basin, and propose that its nascent nature is due to a southward shift of the Tarim’s Euler Pole following the Indian-Tarim lithospheric contact, which changed the stress field and slowed down the platform-cutting strike-slip faults, such as the prominent Talas Fergana Fault. We further propose a hierarchical triangular wedge model to explain the sense of the strike-slip component motions on individual basin-bounding faults across the southeastern half of the Tian Shan. These conjugate strike-slip component motions across different spatial scales collectively play a more important role in accommodating the shortening and extension of the Tian Shan in this sinistral range-scale shear zone than the dextral slip on the platform-cutting strike-slip faults. By examining our vertical component of the derived surface velocity (Fig. 5), we show how the rates, spatial scales, and mechanisms of uplift are heterogeneous across the mountain belt, with glacial isostatic adjustment potentially directing the localisation of shortening and uplift near the highest peak in southern Tian Shan (Jengish Chokusu, Tomur Feng, or Victory Peak, 7439 m, Fig. 1e).

## Results

### Eastward crustal flow with spatially variable strain

Our derived east velocities show a significant extension of Tian Shan along its strike with an east component rate increasing from south-west-west to north-east-east by ~5 mm/yr (one quarter of the total convergence rate), over a distance of ~2000 km (Fig. 1d). The most significant gradients in eastward velocity occur at the edges of a triangular wedge defined at the mountain belt scale (Fig. 2a), with one edge demarcated by a northeast-trending range-cutting diagonal (Fig. 2c), and the other along the southern range front against the Tarim Basin (Fig. 2e). This north-east trending spatial gradient in velocity runs from the western tip of the sinistral Maidan Fault near the north-western end of the Tarim Basin, through the Lake Issyk-Kul, along the Dzhungarian-Alateau and East Tacheng Faults (Fig. 2e) by the Alatau Range and the Baerluke Mountain (Fig. 1b) at the northwestern boundaries of the Ili, Bortala (BTB) and Junggar Basins (Fig. 3a). At this scale, the Tian Shan seems to be lengthening east-west whilst undergoing north-south shortening, with the material inside this triangular wedge moving eastwards and the rest of the Tian Shan moving slightly to the west (relative to a fixed Eurasia).

About 2 mm/yr of dextral motion can be measured on the Karatau-Talas-Fergana Fault system, but it is distributed across ~200 km on each side (Fig. 1d and 2e), consistent with the multiple branches on eitherside of the fault system (Fig. 2c). The long-wavelength eastward velocity between the Karatau-Talas-Fergana Fault and the Aktas Fault, that partially induces the east velocity gradients on the Karatau-Talas-Fergana Fault (Fig. 2c, e), is not the best constrained due to the lack of GNSS stations in this region. However, the relative eastward motion on the west of the Karatau-Talas-Fergana Fault and westward motion on the east of the Aktas Fault is consistent with anti-clockwise rotation of the block. In the northern Tian Shan, sinistral motion on the Kemin Fault is only perceivable on the east end (Fig. 1d). However, there is a clear opposite convergent motion towards the centre on the Issyk-Kul (Fig. 1d), which, together with the subsidence around the lake (Fig. 1e), supports the lithospheric delamination underneath the Central Tian Shan^26,34^.

Overall, the second invariant of strain rate (Fig. 2j) across the Tian Shan, which is a measure of the current amount of ongoing deformation, is primarily dominated by north-south contraction (Fig. 2f, h), and to a lesser degree accommodated by east-west shear (Fig. 2e, i). Both convergence and shear rates are higher in the western Tian Shan than in the eastern Tian Shan and exhibit significant lateral variation of strain distribution, marked by a sharp transition over Jengish Chokusu, the highest peak in Southern Tian Shan (~42^∘^N, 80^∘^E, Fig. 2j). To the west of Jengish Chokusu (Fig. 3, Profiles 1–2), contractional and maximum shear strain rates share similar spatial patterns. Both contraction and shear are distributed between the northern and southern range fronts and a peak concentration along the Issyk-Kul Lake (Fig. 2h, i). The maximum shear has generally lower magnitudes than contraction throughout the range in western Tian Shan, suggesting most faults here are thrusting obliquely. Exceptions are the sinistral Maidan Fault in southwest Tian Shan, and the sinistral Kemin Fault in northern Tian Shan, where the local shear strain exceeds the local contraction (Fig. 3, Profiles 1–2). To the east of Jengish Chokusu, the strain mostly clusters along the northeast trending diagonal, with increasing maximum shear co-locating with increasing positive dilatation north of the Ili, Bortala and Junggar Basins, (Figs. 2k, l and 3, Profiles 3–5), suggesting a predominantly extensional regime in the northeast Tian Shan, with crustal materials moving northeastward, making space for the elongation of the Tian Shan range.

### Nascent range-cutting shear zone driven by Tarim’s rotation

The extension of the Tian Shan along the range-cutting diagonal cannot be explained by existing kinematic models of deformation. Deformation of the Tian Shan has been thought to partition between north-south crustal shortening within the range on ENE-trending range-parallel thrust faults, anti-clockwise block rotation between NW-trending platform-cutting strike-slip faults, and conjugate strike-slip faulting between the Junggar Basin and Kazahk Platform^16,35–37^ (Fig. 1c). However, these mechanisms do not predict extension along a range-cutting NE-trending diagonal, as documented by Zubovich et al.^5^ and highlighted by our velocity gradients, vorticity and strain rate fields (Fig. 2c–j). Zubovich et al.^5^ hypothesised that, given the lack of normal faulting earthquakes recorded in Tian Shan (Supplementary Fig. S18), any component of extension needs to be accommodated through strike-slip faulting or shear along belt-oblique planes. However, no through-going strike-slip fault system has been identified running through the Tian Shan in a NE-SW orientation. Here we combine our geodetic measurements with independent seismological and geological evidence to argue that there is a mountain-belt-scaled NE-trending shear zone being developed cross-cutting the Tian Shan ranges.

First, apart from the most striking signals in the east velocity gradients (Fig. 2c, e), a NE-trending band of positive (anti-clockwise) rotational rates is the most prominent feature in the vorticity rate field (Fig. 2g), and is pervasive across the entire range. To examine the variation of the shear-zone-parallel velocity across the Tian Shan, we rotate our filtered east and north velocities (Fig. 2c, d) to derive northeast velocities parallel to this NE-diagonal (Fig. 3a), and extract NE velocity profiles perpendicular to the possible shear zone (Fig. 3b–f). The northeast velocity profiles do not exhibit the standard arctangent shapes that are typical of profiles over locked strike-slip faults^38^. Instead, they show almost linear velocity variation over hundreds of kilometers, at the scale of the mountain range, which mimics the collective movement of a zone of parallel strike-slip faults (or parallel oblique thrust faults with strike-slip components) driven by simple shear of ductile lithosphere beneath the brittle crust^39^. Profiles 1–1’ is the most linear through the shear zone as the faults in western Tian Shan are evenly spaced^6^. Profile 4 is also linear because it mostly passes through mountain ranges and only touches the edges of the Bortala, Ili and Bayanbuluk Basins. Profiles 2, 3 and 5 pass through the Issyk-Kul Lake, and the rigid Ili and Junggar Basins^40,41^ with increasing block sizes from west to east, which help localise the strain onto basin-bounding faults and fault zones^16^. The shear zone thus decreases in width from 400 to 600 km in the southwest to ~300 km in the northeast where a characteristic arctangent shape typical of interseismic strain accumulation starts to appear in Profile 5–5’ (Fig. 3b–f).

This overall east-to-west pattern of decreasing length scale of deformation is most likely caused by the clockwise rotation of the rigid Tarim Basin, which has an Euler Pole within the Qilian Shan south-east of the Tarim Basin, according to the latest estimates (96.8–98.1^∘^E, 37.8–38.5^∘^N^42,43^) (Fig. 1a). These updated estimates from regional studies shifted Tarim’s Euler Pole ~1300 km eastward from the previous geodetic estimate within the Tarim Basin in the global model^44^ (Supplementary Fig. S19), meaning that the Tarim Basin is not rotating locally, but moving along an arc centred around the Qilian Shan (Fig. 4a, b). This Euler pole in the Qilian Shan is also ~500 km south of the early tectonic estimate^14^ at the east extremity of the Tian Shan near Hami (Fig. 1b, Supplementary Fig. S19), thus predicting an extra northeastward shear cross-cutting the range instead of only north-south compression perpendicular to range strike. We calculate unit vectors pointing in the tangential directions of this rotation at the locations of the GNSS stations to compare with the azimuths of the GNSS velocity vectors (Fig. 2b). We found remarkable agreement between the GNSS and the tangential azimuths within the triangular wedge outlined in Fig. 2a with an average azimuthal misfit of −4 ± 12^∘^. To the northwest of the cryptic shear zone, the azimuthal misfits become −40 ± 23^∘^. This contrast means that the part of the Tian Shan to the southeast of the shear zone is entirely driven by the clockwise rotation of the Tarim Basin, whereas the part of the Tian Shan to the northwest of this shear zone is not feeling the rotation of the Tarim Basin and is mostly driven by the collective northward push from both the northwestern Tarim and the Pamir^45^.

The direction of Tarim’s push (black arrows in Fig. 3a) is nearly perpendicular to the western tip of the Kalpin Fold-and-Thrust-Belt and oblique to the strike of the Maidan Fault, promoting pure thrust along the southern range-front and sinistral strike-slip fault to dislodge the southwest tip of the triangular wedge from the rest of the Tian Shan for its eastward escape. Where the Maidan Fault and the Kalpin Fold-and-Thrust-Belt meet is the furthest point of contact between the Tian Shan and the Tarim Basin from the Euler Pole, thus experiencing the strongest push. Further along the shear zone, the push is oblique to the range directions south and north of the Issyk-Kul, where the sinistral shear is the most cryptic. In the northeast Tian Shan (Fig. 1e), the far-field push becomes almost parallel to the strike of the NE-striking north bounding faults of the Bortala and Junggar Basins, where sinistral motion on the Dzhungarian-Alateau Fault and the pure strike-slipping East Tacheng Fault becomes apparent again^16,37^ (Fig. 2e). The strike-slip rates on these structures are much slower than the shortening rates on the Kalpin Fold-and-Thrust-Belt because they are closer to the Euler Pole (Fig. 3a) and because a significant amount of stress has been absorbed by shortening within the wedge (Fig. 2b, f, h, Supplementary Fig. S18b).

Next, we analyse the focal mechanisms and epicentres of earthquakes recorded over the past century to identify events with strike-slip components and examine their spatial distribution (Fig. 2d, f, h). All major pre-1976 earthquakes re-analysed using analogue seismograms show thrust focal mechanisms within the western Tian Shan^46–48^, except the left-lateral 1911 Sarez earthquake along the Sarez-Karakul Fault (SKF) in the Pamir^49,50^ and the right-lateral 1949 Khait earthquake in the Tian Shan basement to the north of the Pamir Frontal Thrust (PFT)^51^ (Fig. 2h). For post-1976 earthquakes, we divide the Global Centroid Moment Tensor (GCMT) Catalogue data^52^ into strike-slip (plunge of null axis >45^∘^), thrust (plunge of tension axis >45^∘^), and normal (both plunges of tension and null axes <45^∘^) events. Only three normal events were found in the Tian Shan (Supplementary Fig. S18b). Thrust earthquakes cluster into three range-parallel bands–the northern and southern range fronts, and a band through the Issyk-Kul–coinciding with the three strong negative *ϵ*_*yy*_ and dilatation bands (Fig. 2f), consistent with the distribution of the pre-1976 thrust earthquakes (Fig. 2h). Interestingly, the strike-slip focal mechanisms are almost strictly distributed within the half of Tian Shan southeast of the shear zone (Fig. 3a), and delineate a NE-trending diagonal (Fig. 2d) in line with the range-cutting shear zone that we find. This trend can be extended southwestward, where strike-slip earthquakes of focal depths >100 km cluster due to the subducting slab underneath the Pamir (Figs. 2g and 3a). It is also almost coaxial with the NE-trending sinistral Sarez-Karakul Fault system through the Pamir where the 1911 Mw 7.3 (Fig. 2h), 2015 Mw 7.2 and 2023 Mw 6.8 Sarez earthquakes occurred^49,50,53,54^ and the sinistral Chaman Fault System^55,56^ further southwest in Pakistan and Afghanistan (Figs. 1a and 4b). Even though these three structures are currently offset due to the complex crustal-mantle structure underneath the Pamir^57,58^, their left-lateral shear slip line and anticlockwise vorticity features are remarkably continuous^54,59,60^. As all three deformation zones are driven by the NNE-directed indentation of India relative to Eurasia, we hypothesise that a NE-oriented sinistral shear zone will ultimately develop through the Tian Shan (Fig. 4b), with Tarim relaying India’s push as a secondary rigid indenter^61^, to accommodate the ongoing convergence.

We further compare our vorticity and strain rate fields with reported anisotropy from shear-wave splitting measurements^57,62–65^, which indicate the orientation of the ductile flow beneath the brittle upper crust. In Fig. 2g, we show good alignment between the fast axes of the shear wave splitting measurements from Li and Chen^62^ with the overall NE-oriented anti-clockwise vorticity band. This is consistent with the NE-directed ductile lower crustal flow expected underneath a shear zone driven by the clockwise rotation of the Tarim Basin (Fig. 4a). However, our vorticity and strain rate patterns are at odds with the NW- and NNW-oriented fast axes derived from a two-layer model employed by Cherie et al.^64^. In particular, the NNW-directed lower layer anisotropy, interpreted as the NNW-directed mantle flow driven by the Tarim subduction in Cherie et al.^64^, will not be compatible with our velocity field, unless the ductile portion of the lithosphere and the mantle are flowing almost perpendicular to each other; more likely, the orientation of the lower-layer anisotropy represents frozen fabric between the NW-trending suture zones generated during the Paleozoic closure of oceanic basins (Fig. 2i), rather than current flow direction. Anisotropy from Kufner et al.^57^ highlights the NW-trending Talas-Fergana Fault (Fig. 2e), which is posited as a smaller-scaled, more-matured mirror reflection of the nascent NE-trending shear zone. It has a left-stepping kink against the Fergana Basin, likely due to the anticlockwise rotation of the Fergana Block driven by Pamir that co-produced the 100 km Tertiary displacement along the Talas-Fergana Fault^66^(Figs. 1a and 4b). Fast axes from Huang et al.^63^ outline the southern range front against Tarim, the southern bounding fault of the Junggar Basin, and the Dzhungarian-Alateau Fault bounding the Bortala Basin in the north (Fig. 2e), pinning the edges of the range-scale triangular wedge that we identify (Fig. 2a).

To illustrate how the flow fabric is in line with the NE-trending shear zone, we plot SKS shear-wave splitting measurements from Wolf et al.^65^ (Fig. 2j), reduced by 1-arc-degree block mean, and those from Huang et al.^63^ and Li and Chen^62^ on top of our NE-velocity (Fig. 3a). Stations from Wolf et al.^65^ cover the part of the Tian Shan northwest of the shear zone. Fast axes generally trend 60–70^∘^N, oblique to the local range strike (90–100^∘^N) of the Kyrgyz Range in northwest Tian Shan (Fig. 1b). We interpret this mismatch as arising from either the orientation of the Terskey Suture from the Paleozoic (Fig. 2i) or from the anticlockwise rotational indentation of the Fergana Basin that co-produced the left-stepping kink on the Talas-Fergana Fault (Fig. 4a). However, these 60–70^∘^N fast axes are parallel to those along the Pamir front, consistent with a compressional stress field in the mantle induced by Pamir’s push. Anisotropy results from Li and Chen^62^ can be split into two groups, one trending near east-west, parallel to the Terskey Suture (Fig. 2i), the other zigzagging towards the northeast, following the NE-trending immature shear zone. Collectively, they piece together a possible route connecting the shear zone from Pamir to the Maidan Fault in the southwest through to south of the Issyk-Kul of the Ili, west of the Ili Basin and then north of the Bortala and Junggar Basins. The lack of such observations towards the NE Tian Shan is partly due to data availability issues as shown in the uneven spatial distribution of stations allowing for measurements in Wolf et al.^65^. The more spread-out distribution of the NE-oriented fast axes across the range in western Tian Shan is consistent with our interpretation that the western Tian Shan is deforming as a broad and distributed shear zone (Fig. 4a).

Finally, we compile paleomagnetic vertical-axes rotation angles since the Miocene^66–68^ to interpret the spatial patterns of the Neogene rotation with respect to the position of the NE-trending shear zone (Fig.3a). It is clear that the Chaktal Range and the Fergana Basin to the west of the Talas-Fergana Fault have been rotating anticlockwise, in line with the anticlockwise rotation of the rigid Fergana Basin that generated the left-stepping kink on the Talas-Fergana Fault^69^. To the east of the Talas-Fergana Fault, there is insignificant anticlockwise rotation in the Kyrgyz Range, Naryn Basin and southwest of the Issyk-Kul^66^, all to the northwest of the NE-trending shear zone, consistent with the fact that this NW wedge of the Tian Shan does not feel the push from the clockwise rotation of the Tarim Basin (Fig.4b). Similarly, insignificant clockwise rotation has been reported at the Turpan Basin in southeastern Tian Shan^67^, because it is out of the rotational stress field of the Tarim. Anticlockwise rotation angles have been recorded south of the sinistral Maidan Fault, within the Kuche Depression along the southern range front, and north of the dextral Dzhungarian Fault^68^, seemingly contradicting the expectation from the clockwise rotational push from the Tarim. However, this observation is consistent with our proposal of a hierarchical conjugate strike-slip wedge model for the part of the Tian Shan southeast of the shear zone (Section “A hierarchical triangular wedge model for crustal escape”). These anticlockwise rotations are evidence of local small-block rotations^68,70^ that together enable the eastward escape of the southeastern Tian Shan crustal material from the bulldozing of the Tarim.

Without an inherited through-going suture zone oriented parallel to the shear zone, the NE-oriented shear zone is currently releasing shear strain on small segments of pre-existing faults in sub-optimal orientations, a pattern reminiscent of the nascent stage of the evolution of large-scale strike-slip faults^71^. Both our vorticity field (Fig. 2i) and kinematic modelling with 20 micro-blocks within the Tian Shan^72^ predict fast anti-clockwise rotation along the NE diagonal. An expected outcome of this preferential rotation is that the ENE-trending range-parallel thrust faults will be more optimally aligned to accommodate the NE-oriented shear. This could involve a mechanism through which an extensive strike-slip fault eventually develops in a northeast orientation, operating like the large-scale, left-lateral, strike-slip faults in the Tibetan Plateau. By that time, we would see more of the strike-slip seismicity propagating through the interior of the Tian Shan. Consequently, a plate-scale NE-oriented shear zone going from the existing Chaman Fault zone in the south-west all the way through to northeastern Tian Shan^59,60^ would coalesce, demarcating the western boundary of the India-Eurasia collision zone.

Thus, based on the alignment of vorticity field and anisotropy results with the stress field, the distributed nature of shear strain and strike-slip seismicity, and the lack of optimally-oriented mature faults to accommodate the shear strain, we have possibly captured the early days of the development of a shear zone across the Tian Shan, following the successive northward propagation of the development of large-scale sinistral strike-slip faults across the Tibetan Plateau^73,74^. Over geological time, the stepping short segments of strain concentration along the NE diagonal of the Tian Shan could rotate, join and straighten up through local block rotation and basin closure, in the same manner as how the Haiyuan Fault of Northern Tibet lengthened over time^75^. As a result, the northeastern end of this cryptic shear zone could develop into a through-going left-lateral strike-slip fault pushing against the crystalline Kazakh Platform in a pattern similar to how the Altyn Tagh Fault currently is established against the rigid southern margin of the Tarim Basin^76^. As the deeply rooted rigid Tarim Basin continues to rotate around its Euler pole located to the southeast of itself at a rate of 0.6–0.7^∘^/Myr^42,43^, it will push into the weaker Tian Shan crust. This will leave behind a growing boundary of rheological contrast along the southwest portion of the shear zone between the weak Tian Shan material and the strong Tarim Basin in a similar fashion as how the Chaman Fault is juxaposed against the Indian Plate (Fig. 1a). The two ends of the shear zone might not be perfectly aligned, in which case the broad zone of distributed deformation in Western-Central Tian Shan might first deform into a shape similar to today’s Pamir. However, as long as India maintains its push, it is plausible that a through-going shear zone will eventually cut through the Tian Shan along the north-east diagonal, like how the Sarez-Karakul Fault is shearing apart the Western and Eastern Pamir.

### Shifted Euler Pole from Indian-Tarim collision?

We further explore the possibility of the southward shift of Tarim’s Euler Pole being the cause of the “nascent” nature of the range-cutting sinistral shear zone through the Tian Shan, and compare this tectonic transition in Tian Shan with that in a similar tectonic regime in the European Alps (Fig. 4c). In the Alps, the NNW-headed far-field indenter of the African Plate is driving the anticlockwise rotation of the Adriaic microplate to create a right-lateral shear zone that is the Periadriatic Line through the Alps^77^, mirroring how the NNE-headed India is driving the clockwise rotation of Tarim to create a left-lateral shear zone in Tian Shan. Both Adria and Tarim are promontories of much larger plates driving their motion. Due to irregular geometry and the random position of the secondary indenter relative to the first indenter, the torque generated by their collision could change the Euler Pole of the secondary indenter, hence changing the tectonic regime at its plate boundaries. Adria’s Euler Pole was in Spain in the far west, when it first translated northwestward to indent Europe, causing the westward extrusion of the western Alps and the formation of the dextral Periadriatic Line^77^. After wedging the Adriatic toe in the western Alps, the Euler Pole changed to the Po valley to allow further northwest motion by indenting the Adriatic heel into the eastern Alps, driving present-day right-lateral shear around the heel^78^, in the same way that Tarim is driving the cryptic shear zone in the Tian Shan. It is therefore plausible that Tarim’s Euler Pole was indeed near Hami^14^ (Fig. 1b) at the onset of Tian Shan’s shortening around 16 Ma^79^, facilitating the north-south shortening that increases to the west through a clamping motion^79^. The southward drift of the Euler Pole to the Qilian Shan might be a result of the direct contact between the Indian lithospheric mantle and the western Tarim Basin^80,81^ in the late Miocene (~10 Ma)^82^(Fig. 4b). This change of tectonic regime triggered accelerated exhumation throughout the Tian Shan^13,45,83–87^, and a reversal of the paleomagnetic rotations from clockwise to counterclockwise at ~12 Ma along the southern edge of the Tian Shan^70^.

The direct contact between the Indian and Tarim lithospheric mantle could also explain the slowing down of the right-lateral slip rates on the Talas-Fergana and Karakoram Faults (Fig. 4b). The prominent right-lateral Talas-Fergana fault bounding the western Tian Shan, which had higher geological slip rates of 2.2–16 mm/yr^19,20,23^, is currently geodetically moving at a much slower rate of ~1.5 mm/yr^16^. In our results, the change in east component of velocity across the Talas-Fergana Fault is  ~ 2 mm/yr, which translates to ~2.3 mm/yr of slip rate distributed over a 200 km zone (Fig. 2a, f), playing a less important role in accommodating shortening and lengthening than the NE-directed nascent shear zone identified in this study with over 10 mm/yr of velocity change distributed over 400 km of mountain range (Fig. 3a). Similarly, the Karakorum fault of the Western Tibetan Plateau, (Fig. 4b) which had geological slip rates of 4–8 mm/yr^88–90^, is geodetically inactive in the north and slowly slipping at 1.6–3.2 mm/yr in the south^91^. Cenozoic strike-slip motion on both the Talas-Fergana and Karakoram Faults commenced ~25 Ma, synchronous with the onset of exhumation and dextral strike-slip faulting along the eastern flank of Pamir and western Tarim^92,93^. These right-lateral slips once played an important role in accommodating the Pamir’s northward indentation that separated the Tarim and Tajik Basin^94^ (Fig. 1b). That was when magmatism clustered in southern Tibet due to the delamination of the Tibetan lithospheric mantle, before the cold Indian lithospheric mantle shut off the heat from the asthenosphere at ~10 Ma^81,82^. As the Indian lithospheric mantle came into direct contact with the rigid deep lithospheric root of Tarim, like how the Indian and Asian lithospheric mantle are locking underneath Pamir today^57,58^, Pamir and the Western Tarim effectively became a united collisional front. They move north at similar rates, slowing the right-lateral strike-slip motion on the once-highly-active Talas-Fergana and Karakoram Faults. Hence, very limited strike-slip motion was recorded between Pamir and the Tarim since ~11 Ma^95^.

The roles of the NW-striking platform-cutting dextral faults (KtF, DhF, AkF, DzF,and ChF in Fig. 1b, c) likely also diminished with the changing stress field due to the rotation of the Tarim Basin. It was estimated that the Tarim Basin has rotated clockwise by 7 ± 2.5^∘^ since the late Cenozoic, assuming an Euler Pole near Hami^14,79^ (Figs. 1b and 4b). If the Euler Pole were indeed around Hami near the onset of Tian Shan’s reactivation, the stress field felt by the Kazakh Platform would have been northward compression (Supplementary Fig. S20a, b), which promotes the development of the parallel NW-trending platform-cutting strike-slip faults. These dextral faults likely formed trending ~N30^∘^E^96^ and accommodated shortening by anticlockwise block rotation^22,23,97^, hence rotating themselves to be NW-trending. We do not think Tarim’s Euler Pole has always been in the Qilian Shan, as if so, the platform-cutting dextral faults could not have developed into the magnificent structures they are, and the nascent shear zone that we describe would have had more time to mature (Supplementary Fig. S20c). Hence, it is likely that the southward shift of Tarim’s Euler Pole to the Qilian Shan, due to the India-Tarim lithospheric contact around 10 Ma^80,81,98^(Fig. 4b), rotated the stress field to point northeasterly (Fig.3a). This new stress field bypasses the platform-cutting dextral faults. Hence the present-day strain rate on these structures becomes negligible (Figs. 1d and 2a, c). The Talas-Fergana Fault is still active^23^ because of the differential north-south shortening rates in the Tian Shan that still exist north of Pamir and Tarim, respectively^5^. The Dzhungarian Fault is still active^22,97^ due to its new role in facilitating the eastward translation of the Junggar Basin (Section “A hierarchical triangular wedge model for crustal escape”).

### A hierarchical triangular wedge model for crustal escape

We further generalise the conjugate strike-slip faulting model often used to describe the shortening in the northeast Tian Shan^16,37,72,99–103^ and other convergent zones^104–106^ to propose a hierarchical wedge model that describes the eastward crustal flow within the range-scale triangular wedge bounded between the NE-trending diagonal and the southern range-front fold-and-thrust belts (Fig. 2c). Instead of requiring all basin-bounding faults to be pure strike-slipping, we can consider only the strike-slip components on the basin-bounding faults and describe how they form conjugate pairs to facilitate the range-scale shear and extension in addition to the north-south shortening (Fig. 4d). Depending on the orientation of the triangular wedges under consideration, the two edge faults can be both oblique thrusting, a combination of pure thrusting and strike-slipping, or conjugate strike-slipping, as long as the slip directions of at least one of the two limbs accommodate an east component motion within the wedge relative to Stable Eurasia. This relaxed requirement better explains the distribution of the thrust and strike-slip focal mechanisms (Fig. 2d, f, h), where, despite thrust earthquakes occurring throughout the Tian Shan range, strike-slip earthquakes only occur within the triangular wedge southeast of the shear zone. This hierarchical wedge model characterises the structural geometry and kinematics of many crustal wedges in Tian Shan, facilitating the eastward displacements of the Kalpin thrust system, Baicheng-Kuche thrust system, Yanqi, Kumysh, and Turpan Basin from the west to the east along the southern range front, the Zhaosu, Bayanbuluk and Nalati Basins in the Tian Shan interior, and the Chilik Valley, Ili Basin, Bortala Basin and Junggar Basin in the northeastern boundary (Figs. 3a and 4d). All these structures are smaller-scaled east-opening triangular wedges nested within the largest triangular wedge between the nascent shear zone and the southern range front, allowing continuous lateral extension and eastward displacement at the large scale in response to the north-south crustal shortening.

This hierarchical wedge model, by allowing the small wedges to respond to the stress field in their own way, makes it possible to explain the counterclockwise vertical-axis rotations reported along the southern edges of the Tian Shan and of the Junggar Basin (Fig.3a). Both Huang et al.^70^ and Charreau et al.^68^ believe that these fast anticlockwise rotations represent localised small-block rotation. Indeed, if the entire southeastern half of the Tian Shan were to systematically rotate like a rigid block, the paleomagnetic records across the wedge would all show clockwise rotation like Tarim, for which we found no evidence in the literature. The localised rotations and fault slips likely represent the optimal way through which the southeastern Tian Shan responds to the new stress field, exploiting pre-existing structures that happen to be conjugate to each other. To further test the nascent nature of the NE-trending shear zone, it would be interesting to see evidence of reversals of paleomagnetic rotations^70,107^, or initiation/speed-up of anticlockwise rotation along the NE-trending diagonal of the Tian Shan that indicates a transition of tectonic regime around ~10 Ma. Such evidence is currently lacking from the interior of the Tian Shan.

The kinematic setup we describe across the Tian Shan has early similarities to what is ultimately seen in western Tibet between the more established sinistral Altyn Tagh fault and the Himalayan Frontal Thrust (Fig. 4b). The conjugate strike-slip motion on the basin-bounding faults in Tian Shan mirrors the smaller conjugate strike-slip faults on the Tibetan Plateau that facilitate the relative eastward motion of Tibet^108,109^. The right-lateral fault bounding the Yanqi Basin in the south^110^, between the southeastern Tian Shan and the northeastern Tarim, mimics the right-lateral Red River^111^ and Saigang^112^ Faults southeast of Tibet. The reduction of kinematic roles played by the northwest-trending right-lateral strike-slip faults is likely balanced by the strengthening of alternative mechanisms that facilitate the eastward crustal motion. Such mechanisms could include the shear motion on the Altyn Tagh fault and the nascent shear zone through central Tian Shan, north-south trending normal faulting in southern Tibet, conjugate strike-slipping in central Tibet, and the conjugate oblique strike-slip faulting bounding the east-opening triangular wedges in southeastern Tian Shan^24^. However, since Tian Shan has not reached sufficient gravitational potential energy, shortening and reverse faulting still occur within Tian Shan, whereas normal faulting occurs within the Tibetan Plateau^6^.

The triangular wedge model contrasts with the bookshelf model traditionally employed to describe the parallel dextral faulting in the Kazakh Platform (Fig. 1c). The bookshelf model predicts the strike-slip motion on parallel new faults generated in a pristine and rigid material under a compressional stress governed by Anderson’s theory of faulting^113^. Our hierarchical wedge model predicts the sense of strike-slip component motions on pre-existing faults in response to a changing stress field. Our model does not predict slip rates or the sense of dip-slip component motion on the faults. The bookshelf rotation describes well the platform-cutting NW-striking dextral faults and was likely the dominant lengthening mechanism when Tarim had a Euler Pole near Hami and exerted a northward compressional stress through the Kazakh Platform. After the southward shift of Tarim’s Euler Pole, the stress-field bypassed the Kazakh Platform (Fig. 4b). The eastward crustal escape through the conjugate component strike-slip motions along the various-sized triangular wedges within the southeastern Tian Shan became a more dominant lengthening mechanism. The different styles of tectonic response come from the different rheologies characterising the northwest and southeast halves of the Tian Shan orogeny. To the north of the NE-trending shear zone, the Tian Shan is pushing against the Kazakh Platform where the crystalline basement^98,114,115^ promotes brittle faulting along parallel strike-slip faults^116,117^. To the southeast of the shear zone, except the Junggar Basin, which acts mostly as a rigid block^41^, the weaker Tian Shan material^118^ can deform more continuously, distributing itself to the east where there is no hard boundary^119^. Therefore, our observation and conceptual model are also consistent with the dynamic models that suggest how stress orientation and rheological structures together control the deformation fields^76^.

### Tectonic, climatic, and anthropogenic interplay

Apart from the tectonic process that are explained by the bulk of our long-wavelength observed east velocity and strain rate fields, our velocities also reveal signals from myriad other processes at different spatial scales (Fig. 5). One signal is the rapid eastward and westward motions observed on opposite slopes of north-south-trending ridges (Fig. 5a–d). These horizontal movements can be up to 8 mm/yr on each side, positive in the down-slope directions (Fig. 5A–A’). Such movements are observed where the ridges are predicted to have permafrost under most conditions (Permafrost Zonation Index, PZI > 0.5) and the valleys are predicted with permafrost only in favourable conditions (PZI < 0.5)^120^. These signals are typical of the down-slope solifluction movement due to permafrost’s freeze-and-thaw cycles which are also observed in the Qilian Shan^121,122^ and across Iceland^123^, and can be modelled as a combination of seasonal frost creep and basal soil shearing due to the thawing of the ice-rich layers^124^. Similar motion is expected on the north-south slope of east-west trending ridges affected by the same process, although Sentinel-1’s observation geometry limits our vision in the north-south direction. These signals are kilometre-scaled and highly correlated with slope aspects, making them uniquely identifiable (e.g., through high-pass filtering) without obscuring larger-scale tectonic interpretations. They also have the potential to improve our understanding of permafrost dynamics with associated changes in water resources, sediment transport, and landslide risk.

From our vertical component of velocity, the greatest rate of uplift is observed over the highest peak in the southern Tian Shan. This region is highly glaciated with glaciers of kilometre widths that thin at rates up to 4 m/yr^125^. With Sentinel-1, we measured up to 4 mm/yr of uplift over a 250 km wavelength. This large-scale signal is likely a result of glacial isostatic adjustment due to the ice unloading^126^. However, unlike the dome-shaped uplift observed in the east-west range-parallel profile (Fig. 5, Profile B–B’), the range-perpendicular profile north-south reveals a sharp step in velocity of ~2.4 mm/yr (Fig. 5, Profile C–C’). The 2.4 mm/yr vertical velocity step is matched by a stark elevation drop and a drainage divide, which also coincides spatially (Fig. 2i) with the Southern Tian Shan Suture^12^. This co-location of geomorphic, structural and kinematic features suggests the presence of a fast-moving south-dipping thrust fault, controlled by a Paleozoic suture, actively absorbing shortening from the range interior. This is likely the location of the Nalati Thrust Fault (marked by red triangles in Fig. 5e) which has not been mapped in detail due to perennial snow cover in this region. Unlike the previous mapping of this fault being north-dipping^16,127^, like the southern frontal fault-and-thrust belts (Fig. 4b, d), our velocity step suggests that this structure is most likely south-dipping and outcrops to the north of the highest peak. The sharpness of the velocity step suggests that this fault is almost creeping to the surface, possibly facilitated by the lubricated surface of the suture zone^128^. The fast thrust motion could potentially result from climatic-tectonic interplay where the glacial isostatic adjustment (GIA) causes a buoyancy that promotes slip on the fault^129–131^; in this case, creep on the fault because of the suture zone. To our knowledge, no such case has been reported or modelled before. Characterising this interplay might require coupling a finite-element GIA model with a tectonic stress field with faults^132^ with finite-element rate-and-state modelling with a velocity-strengthening suture zone^133^, which is likely implementable through the Pylith package^134^.

It is worth noting that, to the south of the highest peak, lies the only extent in longitude along the southern range front without a fully-fledged fold-and-thrust belt (Fig. 5a). This structural gap thus separates the Kalpin thin-skinned nappe to the west from the Baicheng-Kuche thick-skinned fold-and-thrust belts to the east^9^. The fact that we only observe rapid thrust rates over a short segment of the Nalati Thrust Fault might explain this separation. The preferential accommodation of shortening and uplift to the north of the highest peak may locally reduce the need to develop an extensive nappe system immediately to the south of the peak. The step change in where the shortening is being accommodated could then explain the presence of a NNW-trending right-lateral strike-slip Kalayuergun Fault connecting the highest peak and the Kuche fold-and-thrust-belt (Figs. 3a, 4d, and 5f)^135^, which collocates with the negative vorticity (clockwise rotation) we observe (Fig. 2g). This, together with the Tarim rotation with its Euler Pole to the southeast, explains why the overall shortening rate is drastically reduced from the western Tian Shan to the eastern Tian Shan, delineated by the highest peak (Fig. 2f, h, j).

We observe another long-wavelength uplift signal perpendicular to the elongated agricultural region north of Urumqi (Fig. 5g). Here, intensive groundwater extraction over an area of over 21,000 km^2^ is driving rapid subsidence over the cropland at rates up to 80 mm/yr (Fig. 5, Profile D–D’). The water loss from the aquifer is fast enough to induce 2 mm/yr of uplift over a 500 km wavelength (Fig. 5, Profile E–E’). The uplift signal is clearer into the Junggar Basin to the north than into the Borohoro Mountain in the south. Further work is needed to model the uplift relative to the rate of groundwater extraction^136^. Assuming symmetric rebound to the north and south due to the groundwater extraction, we resolve an additional 1–2 mm/yr of uplift in the Borohoro Mountain, likely due to the crustal shortening in Eastern Tian Shan.

Profiles B–B’ and E–E’ of vertical velocity are similar in that they both show uplift in response to a local removal of mass, one of the glacial ice from the surface, and the other of groundwater from the shallow crust. Both uplift signals are across a wavelength that is about two orders of magnitude longer than the widths of the removed mass. Both uplift signals result from a combination of tectonic and non-tectonic processes, requiring models coupling multiple mechanisms to explain and investigate further^137,138^.

## Discussion

Our high-resolution, large-scale east and vertical velocities derived from the combination of Sentinel-1 InSAR and GNSS data allow us to investigate the current kinematics and complex deformation mechanisms across the entire mountain belt of the Tian Shan in unprecedented detail, revealing processes that induce deformation at different spatial scales.

At the mountain range scale, horizontal gradients of the east velocities revealed the location of a potential through-going shear zone developing in the lower crust of the entire Tian Shan, in response to the indentation and rotation of the Tarim Basin about its Euler Pole to its southeast. This shear zone, together with the southern range front, outlined a triangular wedge of east-moving materials in the southeast half of the Tian Shan. The distributed and continuous deformation and seismicity observed within the triangular wedge invoked a kinematic model that resembles a hierarchical pattern of crustal wedges bounded by major-to-minor faulting at different spatial scales. This hierarchical wedge model characterising the deformation southeast of the shear zone contrasts with the pre-existing bookshelf rotation model used to describe deformation in the Kazakh Platform because of the different rheological controls exerted by the north-bounding crystalline Kazakh Platform versus the more deformable crustal materials of the Tian Shan and the Altay to the east. The bookshelf-style deformation in the Kazakh Platform was likely more active when Tarim’s Euler Pole was near Hami, if Tarim’s Euler Pole was indeed centered relatively further north at an earlier stage of the Indo-Eurasian Collision. What is demonstrated in this study is that the wedge-style deformation in the southeast half of the Tian Shan is a more dominant mechanism accommodating Tian Shan’s lateral extension at the present day, with Tarim’s Euler Pole in the Qilian Shan.

At the kilometre scale, our detrended east velocities reveal rapid down-slope creep motions that have a horizontal component up to 8 mm/yr on both sides of north-south-trending ridges in the permafrost zone. The rate of movement is slower than that of typical slow landslides^139^. The horizontal components of the motion also differentiate the mechanism from simple gravitational compaction of unconsolidated sediments^140,141^. What we observed is most likely solifluction and/or frost creep^121^, possibly facilitated by basal sliding^124^, highlighting the rapid rate of permafrost degradation under a changing climate. Potentially similar rates of motion also occur on the north and south slopes of east-west-trending ridges, which we are currently missing due to the near-polar orbit and the two observation geometries of Sentinel-1 satellites alone.

At between kilometre to hundreds-of-kilometre scales, vertical velocities exhibit long-wavelength uplift signals in response to rapid deglaciation and groundwater extraction that might be overprinting signals of, or in the case across the highest peak interacting with, the rate and style of mountain building and faulting activities, providing an opportunity to study the interplay between tectonic, climatic and anthropogenic activities^126,129,136,142^.

Our results highlight the value of high-resolution kinematic data consistently acquired with mountain range to continental scale coverage that can be provided by InSAR, despite currently only imaging with two lines-of-sight and offering only the derivation of east and vertical velocity measurements. Further improvements will be possible now the NISAR satellite joins Sentinel-1 in orbit, together providing observations in four lines-of-sight to enable full-vector high-resolution continental-scale measurements^143^. However, high-quality GNSS velocities will still be essential for mosaicking InSAR velocities over multiple tracks. It is important that we have a good distribution of more than 4 3-dimensional GNSS data at locations we expect InSAR to be coherent to anchor the planar ramp of each InSAR frame. Imperfect referencing due to the lack of GNSS constraints, for example in non-deforming regions around the Karatau-Talas-Fergana Fault and within the Tarim Basin (Fig. 1b, Supplementary Fig. S12), led to strain artefacts along InSAR track boundaries (Fig. 2c). Therefore, it would be desirable for the InSAR and GNSS communities to collaborate strategically, in view of the footprint of radar satellites, to improve the spatial distribution of GNSS data acquisitions. This step is crucial to referencing the next-generation continental-scale high-resolution velocity maps^60^.

Our study shows that large-scale InSAR velocities can be used to infer deeper processes in addition to localised strain on single faults. Whilst ideally it is best to model deformation across single faults from the unfiltered velocities, the robustness of the northeast-trending diagonal despite strong median filtering clearly delineates a boundary in the regional stress field. Our integration of mountain-belt-scale velocity, vorticity and strain rate maps with focal mechanisms, shear wave splitting results, paleomagnetic data and tectonic history further opens opportunities to interpret present-day kinematics as a snapshot of tectonic evolution from the geological deep time into the future. We infer that the cryptic nature of the nascent shear zone is due to a southward shift of Tarim’s Euler Pole due to the collision between the Indian lithospheric mantle and Tarim’s lithospheric root southwest of the Tarim Basin at about 10 Ma, which likely shut off the right-lateral translation along the Talas-Fergana and Karakoram Faults. Testing this hypothesis requires new paleomagnetic vertical axes rotation time series data along the cryptic shear zone. If the above hypothesis were true, we suggest that there is a developing extension on the western boundary of the India Eurasia collision zone propagating through the mountain range of the Tian Shan. Incorporating these observations into future dynamical models of the whole collision zone will improve our understanding of the plate scale boundary conditions and driving forces of this orogeny.

## Methods

### Sentinel-1 data processing

We processed 8.5 years of Sentinel-1 InSAR data acquired between October 2014 to March 2023 to yield line-of-sight (LOS) velocities over 90 frames as defined in the Looking into Continent from Space (LiCS) system^144^ (Fig. 1). The 12 ascending and 12 descending tracks cover a total area of 2.2 million km^2^ and overlap in an area of 1.6 million km^2^ over the entire Tian Shan. Our initial networks for each frame have 1239 ± 434 interferograms connecting 183 ± 45 epochs spanning 7.4 ± 0.7 years and with average temporal baselines of 170 ± 165 days (Supplementary Fig. S1). The inclusion of the long-temporal-baseline interferograms ensures that any phase bias could only minimally play any role in the vertical component.

These ~100,000 interferograms were generated using the LiCSAR processing chain^144,145^. To improve interferogram coherence and network connectivity, we used the Copernicus GLO-30 Digital Elevation Model (10.5270/ESA-c5d3d65) for topographic correction and coregistration, and used the MOD10A1.061 Terra Snow Cover Daily Global 500 m MODIS data^146^ to guide our choice of reference epoch and long-period interferogram generation (Supplementary Text S1, Supplementary Figs. S2–S4). All interferograms were unwrapped using the SNAPHU phase unwrapping algorithm^147,148^ incorporating modified Goldstein filtering^149^. Tropospheric delay was corrected through using the Generic Atmospheric Correction Online Service (GACOS)^150–152^.

### Time series analysis

For time series analysis, we improved methods for coregistration error detection, reference selection, unwrapping error correction, network optimisation, and weighted velocity inversion into the LiCSBAS workflow^153^.

To remove interferograms with coregistration errors that manifest as steep along-azimuth gradients in the unwrapped interferograms with discontinuities between subswaths, we combined detection thresholds based on both gradient and linearity of the azimuthal phase profile and achieved 98% true positive and 5.5% false negative rates (Supplementary Text S2, Supplementary Fig. S5).

To choose the reference window for each frame, we combined four proxies to select a 5 × 5 km^2^ window with the highest pixel coverage, highest average coherence, lowest terrain ruggedness, and in the largest connected component obtained through the SNAPHU phase unwrapping algorithm^147,148^ (Supplementary Text S3, Supplementary Fig. S6).

To handle unwrapping errors, we designed an algorithm to automatically discard, mask, and/or correct interferograms based on the residuals from preliminary time series inversion and facilitated by the connected components (Supplementary Text S4, Supplementary Fig. S7). All interferograms were visually checked to ensure no unwrapping or coregistration mistakes.

We used interferograms with all temporal baselines for the preliminary time series inversion to obtain better-constrained residuals, but removed 6-day interferograms from the final networks to mitigate phase bias that could be induced by short baseline interferograms^122,154–157^.

To ensure connectivity of the final networks, we applied graph theory to select the largest connected component network with >1 edge (interferogram) and node (epoch) connections (Supplementary Text S5, Supplementary Fig. S8). These criteria ensured that the resultant time series was not biased by noise of a single interferogram.

Our final networks have 722 ± 242 interferograms connecting 136 ± 38 epochs spanning 6.8 ± 1.0 years and with average temporal baselines of 178 ± 139 days (Supplementary Fig. S1). The 90 frames achieved an average root-mean-squares residual of 1.5 mm from time series inversion for average velocity (Supplementary Fig. S9).

For velocity inversion from the time series, we drew inspiration from Daout et al.^158^ and Lemrabet et al.^159^ to weight the displacement time series by the range and azimuth ramp coefficients as well as the standard deviation of the deramped cumulative displacement (Supplementary Text S6, Supplementary Fig. S10). This weighting step improved the spatial structure of the velocity uncertainties (Supplementary Fig. S11) and the coherence of the final decomposed east and vertical velocities (Figs. 1d and 5a). After correcting for the reference effect^122^, the 90 frames have average line-of-sight velocity standard deviations of 0.3–1.6 mm/yr (0.7 ± 0.3 mm/yr) (Supplementary Fig. S11).

### Velocity mosaicking, decomposition

For referencing the InSAR line-of-sight velocities, we compiled 673 Global Navigation Satellite System (GNSS) velocities from 22 publications (Supplementary Text S7)^5,26–29,42,43,59,160–173^. All horizontal GNSS velocities were transformed into the Fixed-Eurasia reference frame as defined under the IRTF2014 plate motion model^174^. We cleaned the dataset by uncertainty threshold (0.2 mm/yr) and occupation period and times, grouped them by common station names, and averaged velocities within 2 km radius. To improve InSAR referencing over areas with inadequate GNSS control, we created dummy GNSS points by sampling the interpolated GNSS horizontal velocities in the north and calculating the expected horizontal velocities from Tarim’s rotation in the south (Supplementary Text S7, Supplementary Fig. S12).

After removing the semi-planar ramps in the line-of-sight due to plate motion^175^ (Supplementary Figs. S13–S14), we mosaicked the InSAR line-of-sight velocities track-by-track by tying them to the GNSS line-of-sight velocities through planar ramps^122^ (Supplementary Fig. S15). We then substracted the line-of-sight component from interpolated GNSS north velocities, and decomposed the line-of-sight velocities into east and vertical velocities with weights provided by the line-of-sight velocity uncertainties with reference effects corrected^122^(Supplementary Fig. S11).

### Uncertainty estimation

We estimate the uncertainties of our line-of-sight and decomposed east and vertical velocities exploiting redundancies at track boundaries^60,176^ (Supplementary Fig. S16). The uncertainties of east and vertical velocities decomposed from one ascending and one descending tracks are 2.0 mm/yr and 1.6 mm/yr, respectively, estimated at diamond shapes overlapped by two ascending and two descending tracks. Propagating these numbers, and a conservative uncertainty of 3 mm/yr for north velocities, through the unit vector of the average line-of-sight yields an uncertainty of 1.8 mm/yr for line-of-sight velocities predicted from the east and vertical velocities. At stripes overlapped by three tracks (2 ascending + 1 descending, or 1 ascending + 2 descending), the observed line-of-sight velocities, and those predicted from the east and vertical velocities decomposed from the other two independent ascending and descending tracks, have root-mean-squared differences of 2.6 mm/yr for ascending overlaps and 2.2 mm/yr for descending overlaps. Removing the 1.8 mm/yr of uncertainties of the predicted line-of-sight velocities from these differences yields 1.9 mm/yr and 1.2 mm/yr of uncertainties for observed ascending and descending line-of-sight velocities. Propagating these two line-of-sight velocity uncertainties through the velocity decomposition gives uncertainties of 1.6 ± 0.2 mm/yr and 1.3 ± 0.2 mm/yr for our full east and vertical velocity fields, which compare well with the 2.1 mm/yr and 2.5 mm/yr of RMS differences between InSAR and GNSS east and vertical velocities, respectively (Supplementary Fig. S17).

### Vorticity and strain rate calculation

To calculate the vorticity and strain rate fields, we median-filtered the decomposed east velocity and the north GNSS velocities, first interpolated through *gmt surface*, using a 200 km filter window to suppress noise in the horizontal velocity gradients (Fig. 2c–f). Then, we combined the velocity gradients to calculate the vorticity rate as *dV*_*N*_/*dx* − *dV*_*E*_/*dy* (Fig. 2g), and strain rate fields of the horizontal second invariant, maximum shear, and dilatation in the same way as Ou et al.^122^(Fig. 2h–j).

## Supplementary information

Supplementary Information

Transparent Peer Review file

## Data Availability

We thank the European Space Agency (ESA) for the open data policy on the Sentinel-1 mission. The InSAR data are copyrighted by ESA and are provided through the Copernicus Open Access Hub (https://scihub.copernicus.eu/). The Sentinel-1 InSAR data are also freely distributed by the Alaska Satellite Facility (https://asf.alaska.edu/). The GNSS velocities compiled and the InSAR velocities, uncertainties, vorticity and strain rate fields generated in this study have been deposited on Zenodo https://zenodo.org/records/20293600.

## Code availability

The InSAR data were processed using LiCSAR System, time series inversion was performed using modified LiCSBAS (10.5281/zenodo.20316308). Scripts for frame mosaiking, decomposition and error analysis are archived at 10.5281/zenodo.20316459.
