# Supplementary material for: Extension of Tian Shan along a nascent shear zone
Source: Nat Commun. 2026 Jul 22;17:8943. doi: 10.1038/s41467-026-74965-2 (PMC13503760; doi:10.1038/s41467-026-74965-2)
Supplement: Supplementary file 1 [file 41467_2026_74965_MOESM1_ESM.pdf]

Supporting information for "Extension of Tian Shan along a nascent shear zone"

Qi Ou[1,2]*, John R. Elliott[1], Yasser Maghsoudi[1,3], Chris Rollins[4], Milan Lazecky[1], Tim J. Wright[1]

[1*]COMET, School of Earth, Environment and Sustainability, University of Leeds, Woodhouse Lane, Leeds, LS2 9JT, United Kingdom.

[2]COMET, School of GeoSciences, University of Edinburgh, Drummond Street, Edinburgh, EH8 9XP, United Kingdom.

[3]COMET, Department of Earth and Environmental Sciences, University of Exeter, Penryn Campus, Penryn, TR10 9FE, United Kingdom.

[4]Earth Sciences New Zealand, 1 Fairway Drive, Lower Hutt, 5011, New Zealand.

*Corresponding author(s). E-mail(s): qi.ou@ed.ac.uk;

Contributing authors: j.elliott@leeds.ac.uk; Y.Maghsoudi-Mehrani@exeter.ac.uk; chris.rollins@earthsciences.nz; m.lazecky@leeds.ac.uk; t.j.wright@leeds.ac.uk;

Content:

Text S1-S7

Figure S1-S20

Text S1. Interferogram processing

(1) InSAR mapping over the Tian Shan region suffers from low coherence issues due to seasonal snow cover (Figure S2-S3). To improve coregistration of Single Look Complex data and coherence of all interferograms, we registered all epochs to a primary epoch in August 2020 in the middle of the time series and the middle of the no snow period (Figure S4). The no snow period was constrained using MOD10A1.061 Terra Snow Cover Daily Global 500m MODIS data (Hall and Riggs, 2021). We used this MODIS data spanning 2010-2020 to define the mean first day of the year with no snow cover for each 500 m pixel, and similarly the first day of the year with snow cover for each pixel. The result shows that most snow cover was gone by May and most snow cover arrives from November, hence the choice of August for the primary epoch.

(2) To increase redundancy in the initial networks, we make 3, 6, 9, and 12-month-long interferograms between epochs between May and November, in addition to between each epoch and its five forward nearest epochs. These long-temporal-baseline interferograms are important for mitigating the phase bias induced by short-temporal-baselines (De Zan et al, 2015; Ansari et al, 2020; Maghsoudi et al, 2022; Purcell et al, 2022; Ou et al, 2022) and for ensuring network connectivity of the final networks after removing bad interferograms through time series analysis.

(3) For phase unwrapping, we used the Statistical-cost, Network-flow Algorithm for Phase Unwrapping (SNAPHU) (Chen and Zebker, 2001, 2002) to perform the core phase unwrapping procedure in a processing chain incorporating modified Goldstein filtering (Goldstein and Werner, 1998). To facilitate unwrapping, we combined this step with atmospheric correction and removed atmospheric phase delays given by Generic Atmospheric Correction Online Service (GACOS) (Yu et al., 2017, 2018a, 2018b) before unwrapping, if it reduces the standard deviation of phases in the original interferogram (Lazecky et al., 2022). We retained the connected components by-products of the SNAPHU phase

unwrapping to facilitate reference window selection (Text S3) and unwrapping mistake correction (Text S4) after preliminary time series analysis.

Text S2. Coregistration error identification – improved LiCSBAS11_check_unw.py

Some interferograms suffer from a type of coregistration error that manifest itself as a strong slope in azimuth in unwrapped interferograms (Figure S5a,b). We developed a simple strategy to automatically identify and discard such interferograms by calculating the absolute value of slope (in mm/degree latitude) and linearity (R^2) from the middle column of the interferogram (Figure S5c,d). To fine-tune the default detection threshold, we first divided all interferograms into four categories (|slope|>=20; |slope|=15-20 & R^2 >= 0.9; |slope|=15-20 & R^2 < 0.9; |slope| < 15) based on the distributions of these two values across all Tian Shan frames (Figure S5e,f). After visually checking automatically categorised interferograms and labelling them as real error or not, we find that the threshold of |slope|>=30 & R^2 >= 0.95 gives the best classification result. We also find that such error tends to appear in cluster with association with a common epoch. Most of the Therefore, we implemented |slope|>=30 & R^2 >= 0.95 as the default threshold for detecting this type of error in step 11 of the latest version LiCSBAS workflow (LiCSBAS11_check_unw.py with the -S flag). In this script, we also identify any duplicated epochs in the shortlisted interferograms, and automatically classify as error other interferograms associated with these duplicated epochs if they have |slope|>20. This approach achieved an overall 98% true positive and 5.5% false negative rates, and detected in total 474 interferograms in 29 frames with coregistration error out of over 100,000 interferograms processed over 90 frames. Amongst the 29 frames, 23 frames developed such error in 2014 and 2015, hinting at the association of this error with strong ionospheric effect due to the intense sunspot activities.

Text S3. Multi-criteria Reference Point Selection – LiCSBAS120_choose_reference.py

The original version of LiCSBAS selects reference points on the fly in step 12, step 13, and step 16 (Morishita et al., 2020). This resulted in biases in the masking step 15 based on quality indices derived from previous steps, because the spatial pattern of quality indices, such as vstd, is dependent on the location of the reference point (Ou et al., 2022).

In this study, we developed a robust approach to determine the reference window (instead of a single point), based on four normalised statistics computed per 5 km x 5 km window from all interferograms in the frame (Figure S6). The final chosen reference window has high pixel coverage, high coherence, flat terrain, and tends to fall in the largest connected component given by SNAPHU (Text S1(3)).

Text S4. Automatic unwrapping mistake correction – LiCSBAS131 and LiCSBAS132

This section pertains to two new scripts currently implemented in LiCSBAS: LiCSBAS131_residual_threshold.py, and LiCSBAS132_3D_correction.py.

In an effort to retain more interferograms in the network rather than discarding whole interferograms based on triplet loop closure, as in the original LiCSBAS12_loop_closure.py step, we implemented new approaches to correct unwrapping mistakes and mask noise on individual interferograms. This improves the quality of the time series by eliminating bad pixels and correcting all automatically correctable unwrapping mistakes. We visually quality checked all the corrected interferograms.

The decision on whether and how to correct and/or to mask the interferograms is based on the residual map of each interferogram obtained from a preliminary time series inversion using all interferograms retained after LiCSBAS11. By bypassing the original LiCSBAS12_loop_closure.py, we

give all interferograms a chance to be corrected. The automatic decision tree is implemented in LiCSBAS132_3D_correction.py and illustrated with examples in Figure S7. The four possible decisions are:

- Good interferogram – mask pixels with absolute value of residual/2pi above 0.2
- Bad interferogram – discard
- Integer correction – correct by the nearest integer of residual/2pi, mask ambivalent pixels
- Mode correction – correct each connected component by the mode of the nearest integer of residual/2pi per component, mask ambivalent pixels

The categorisation of interferograms for the four possible treatments is governed by a threshold, which can be provided as an input argument to LiCSBAS132_3D_correction.py (0.15 used in this study), or read from the output of LiCSBAS131_residual_threshold.py.

LiCSBAS131_residual_threshold.py plots a histogram of root-mean-square residuals of interferograms from a frame, and outputs the mean, median, mode and/or a given percentile of the RMS residuals.

Text S5. Network analysis based on graph theory

We borrow ideas from the graph theory, such as connected components, degree of connectivity, edge cuts and node cuts, to the assessment of integrity of our interferogram networks (Figure S8). No matter how robust the interferogram network was at the start, there will be chances where the integrity of the network is compromised after the removal of bad interferograms (Text S4 and Figure S7). Not all types of network weaknesses can be detected by the identification of temporal gaps in the network, as implemented in the previous version of the LiCSBAS_plot_network.py. We improved the script to isolate the largest connected component from the network, identify weak links that represent interferograms in the smaller connected component, interferograms associated with epochs not used in other interferograms (degree of connectivity of 1), and interferograms associated with an edge cut or a node cut. An edge cut or a node cut are an interferogram or an epoch, removing which will increase the number of connected components in the network. When weak link removal will cause too much reduction to a network, we densify the network by generating new interferograms using a new script called LiCSBAS_densify_ifgs.py. This script takes in a list of existing interferograms, or a list of primary epochs and a list of secondary epochs, and creates a list of additional interferograms within a specified range of temporal baselines. The output list is subsampled to the desired number of total additional interferograms required and evenly distributed across the network (Figure S8f). Our final networks have average on average 7-15 interferograms connected to each epoch (degree of connectivity of 11 ± 4), and have no edge cuts or node cuts.

Text S6. Line-of-sight velocity inversion weighted by ramp parameters and residual signal oscillations

Daout et al., (2019) underscored the importance of the weighting the velocity inversion from time series using the residuals from time series inversion and the standard deviation of signal in the deramped cumulative displacement time series. Lemrabet et al., (2023) highlighted the periodicity in the range and azimuth ramp coefficients in the cumulative displacement time series. In our analysis, we noted that occasional significant ramps in the time series can have a major impact on the ramp in the resultant line-of-sight velocities, depending on when in the time series that ramp appeared. This

prevents us from approaching the ultimate goal of reducing reliance on the GNSS velocities for referencing the InSAR velocities.

In the new script LiCSBAS_cum2vel_weighted.py, we borrowed and further developed ideas from Daout et al., (2019) and Lemrabet et al., (2023) to improve the weighting strategy in our velocity inversion (Figure S10). For each temporal step of the cumulative displacement map, we can separate the data into a best-fit planar ramp and the deramped cumulative displacement. The size of the signal in the deramped displacement can be characterised by its standard deviation. This standard deviation will increase over time if a process, such as groundwater extraction causing subsidence, is persistent and accumulates displacement over time (solid green line in the bottom panel of Figure S10). Therefore, we should not downweigh the displacement towards the end of the time series when it comes to weighting the velocity inversion with the standard deviation information. Instead, we used the detrended standard deviation (dashed green line in the bottom panel of Figure S10) as a component of the weight instead.

The planar ramp component of the cumulative displacement map indeed has its range and azimuth ramp coefficients that exhibit linear and seasonal trend over time (orange and blue lines in the top panel of Figure S10). After removing the linear and seasonal trends from the ramp coefficient time series, we have the detrended ramp coefficients time series (middle panel of Figure S10) that are dominated by the solid earth tide 14-day signals and occasional larger deviations (such as the blue spike in early 2022) that could impact the ramp of our velocities, if not taken care of.

Therefore, we combined the two detrended ramp coefficients time series in a root-sum-square manner to produce the red line in the bottom panel of Figure S10, and then combined the red line with the dashed green line to produce the purple line, which we used as the final standard deviation in weighting the time series inversion. The resultant line-of-sight velocities had more consistent trends along track with just a constant offset applied (Figure S13). These trends are similar to that predicted from the plate motion in terms of the along-range tilt (Figure S14), but the gradient along azimuth is different, and that could result in artificial subsidence of the entire southern Tian Shan when decomposed into vertical and east velocities. Therefore, we still needed to use GNSS velocities to reference the InSAR line-of-sight velocities (Text S7).

Text S7. Dummy GNSS data

There are few GNSS data into the Kazakh Platform or into the Tarim Basin. Therefore, the spatial distribution of the GNSS data is inadequate to properly reference the InSAR line-of-sight velocities in the north and in the south. To solve this problem, we adopted two different strategies for improving the GNSS control in the north and in the south.

For the Kazakh Platform in the north, we interpolated the horizontal GNSS velocities from far field using a Kriging algorithm (Ou et al., 2022), and sampled the interpolated velocities and interpolated uncertainties at dummy locations which will improve the geometric control of the planar ramps for both the ascending and descending frames and avoiding InSAR velocities of large uncertainties (Figure S12). This worked well in the north where the Kazakh Platform is largely representing the Stable Eurasia, with no significant strain localisation either expected from literature or observed from our InSAR line-of-sight velocities. Our preliminary tests suggested that referencing the north with interpolated velocities produced better results than assigning zero velocities to dummy GNSS points. This could be because our InSAR coverage has not reached far enough north to cover the most stable region with no velocities in the Stable Eurasia Reference Frame. For all dummy GNSS in the north, we

assigned 0 mm/yr vertical velocities with 1 mm/yr of uncertainties, in the same way we approached 2D GNSS data compiled from literature.

For the southern front, we need to capture the clockwise rotation from the Tarim Basin. This is best done through forward calculation from the rotation rate and Euler pole relative to the Stable Eurasia. For this purpose, we used the rotation rate and Euler pole published by Wang et al., 2020 for it is a study that focused specifically at solving for these two values for Tarim, and indeed the line-of-sight velocities referenced using dummy GNSS calculated from these values produced the most continuous east and vertical velocities after decomposition. As the Tarim Basin is close to the fold-and-thrust belt along the southern range front and itself is experience some uplift within, we set the vertical velocities of the dummy GNSS here to be 0 mm/yr with 2 mm/yr uncertainties.

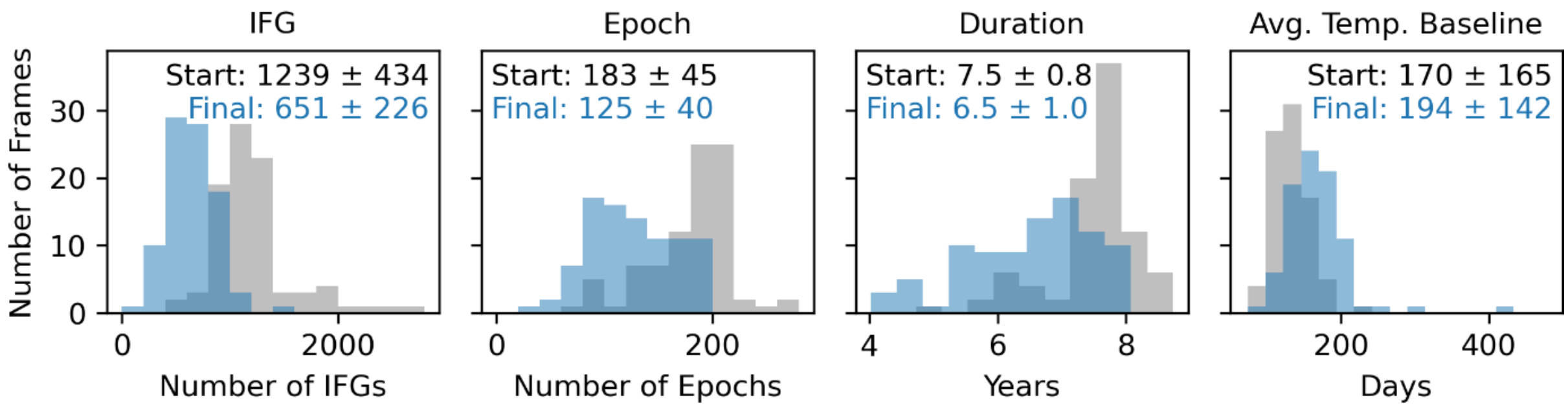

Figure S1: The per-frame statistics of number of interferograms, epochs, time span and average temporal baselines of interferograms before and after the quality control, through the improved LiCSBAS11 (Text S2) and newly developed LiCSBAS132 (Text S4) steps during the time series analysis.

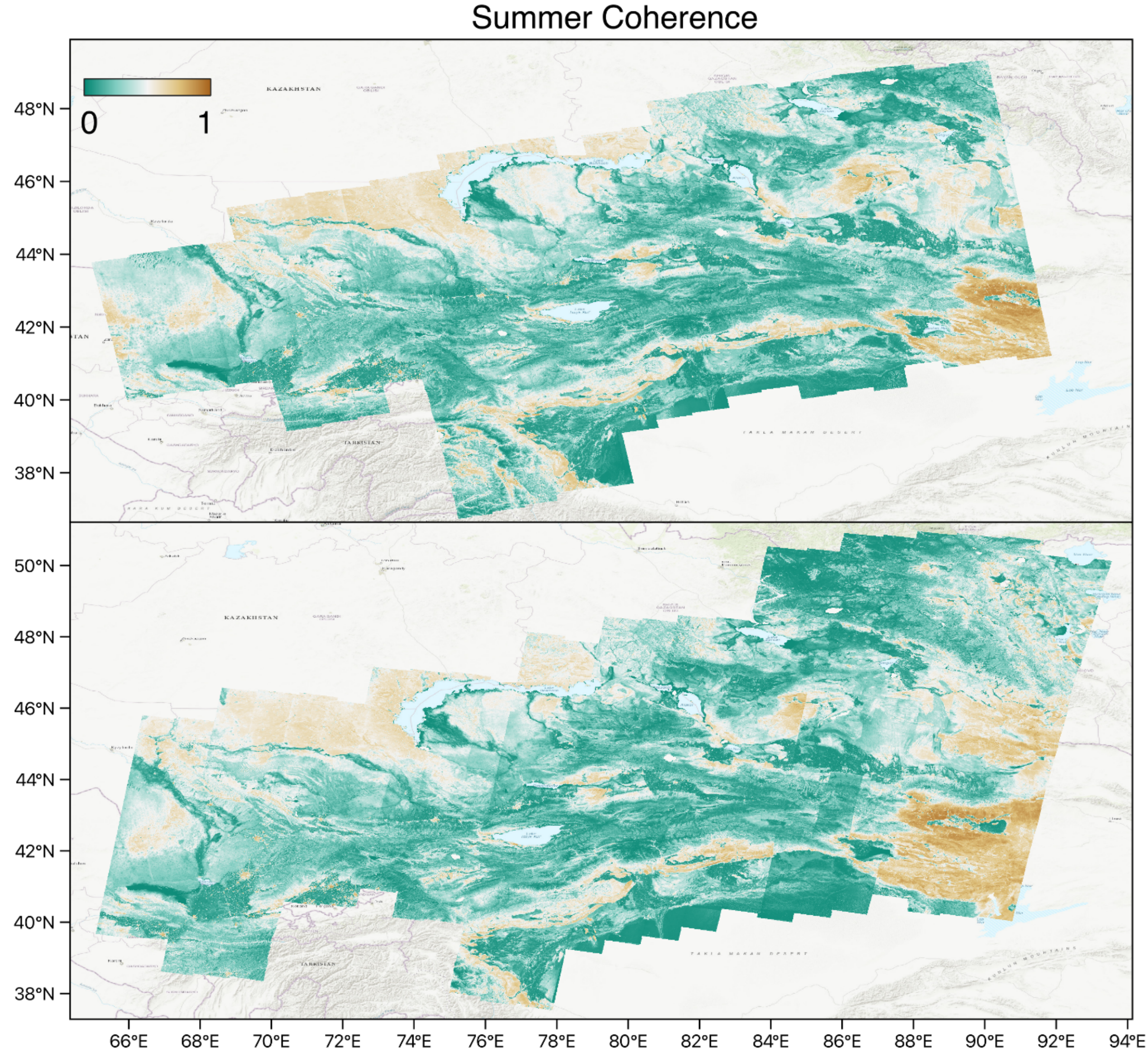

Figure S2: Average coherence of all interferograms with both epochs between the summer months from May to October.

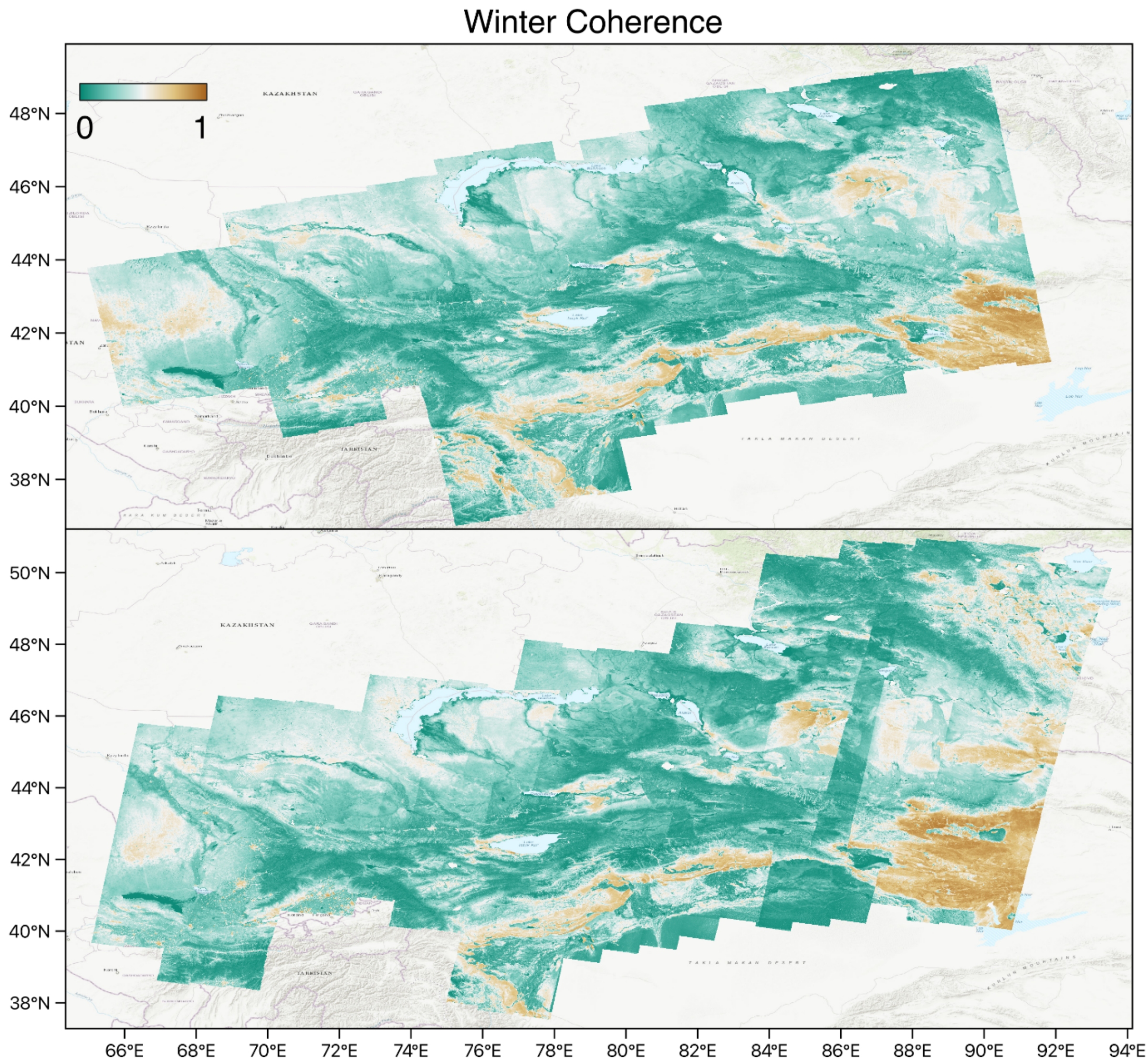

Figure S3: Average coherence of all interferograms with either epoch between the winter months from November to April.

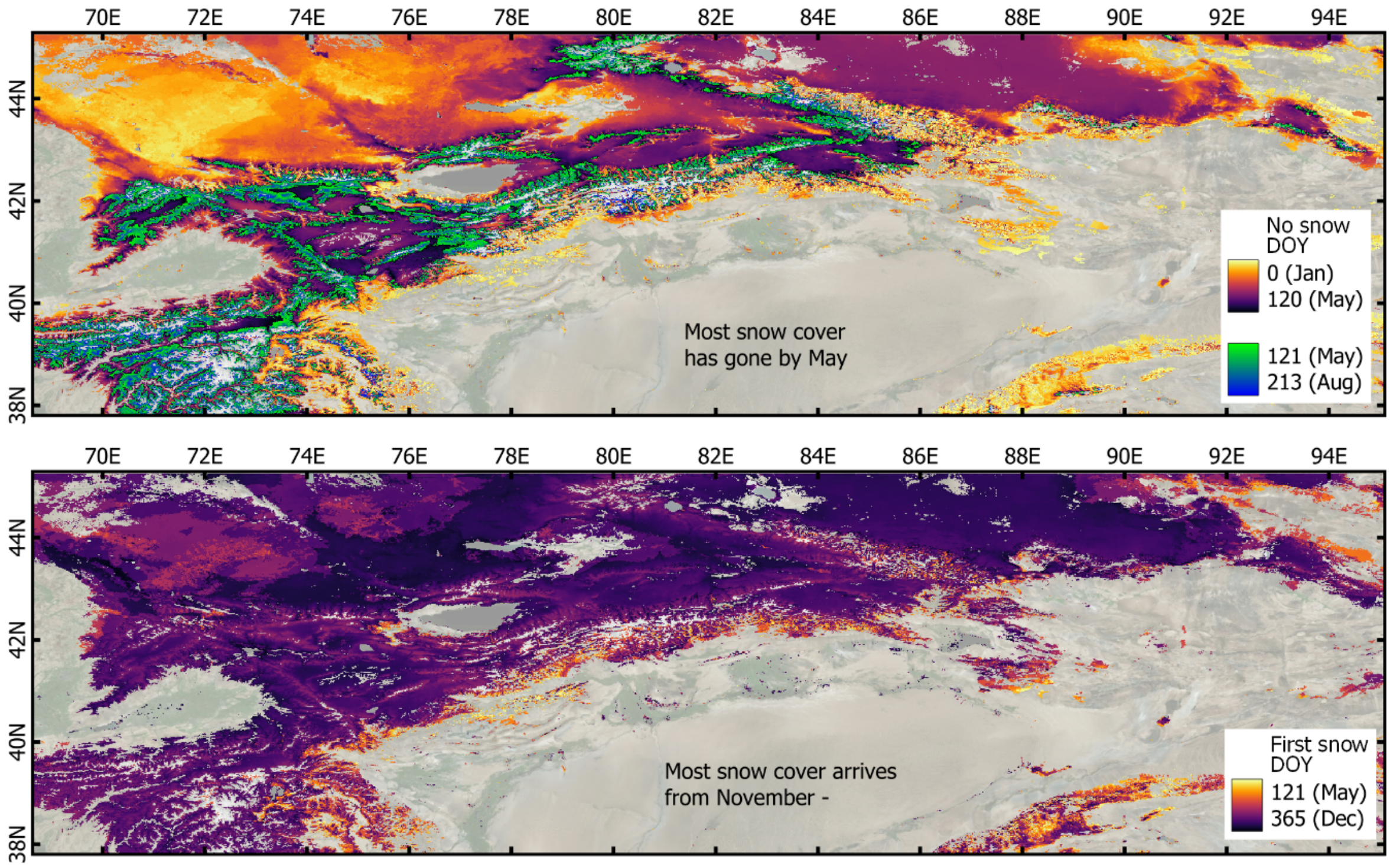

Figure S4: MODIS data used to guide the choice of reference epochs. Mean first day of year of no snow and first day of year of snow cover per pixel in the Tian Shan, based on MOD10A1.061 Terra Snow Cover Daily Global 500 m MODIS data between 2010-2020 (Hall and Riggs, 2021)

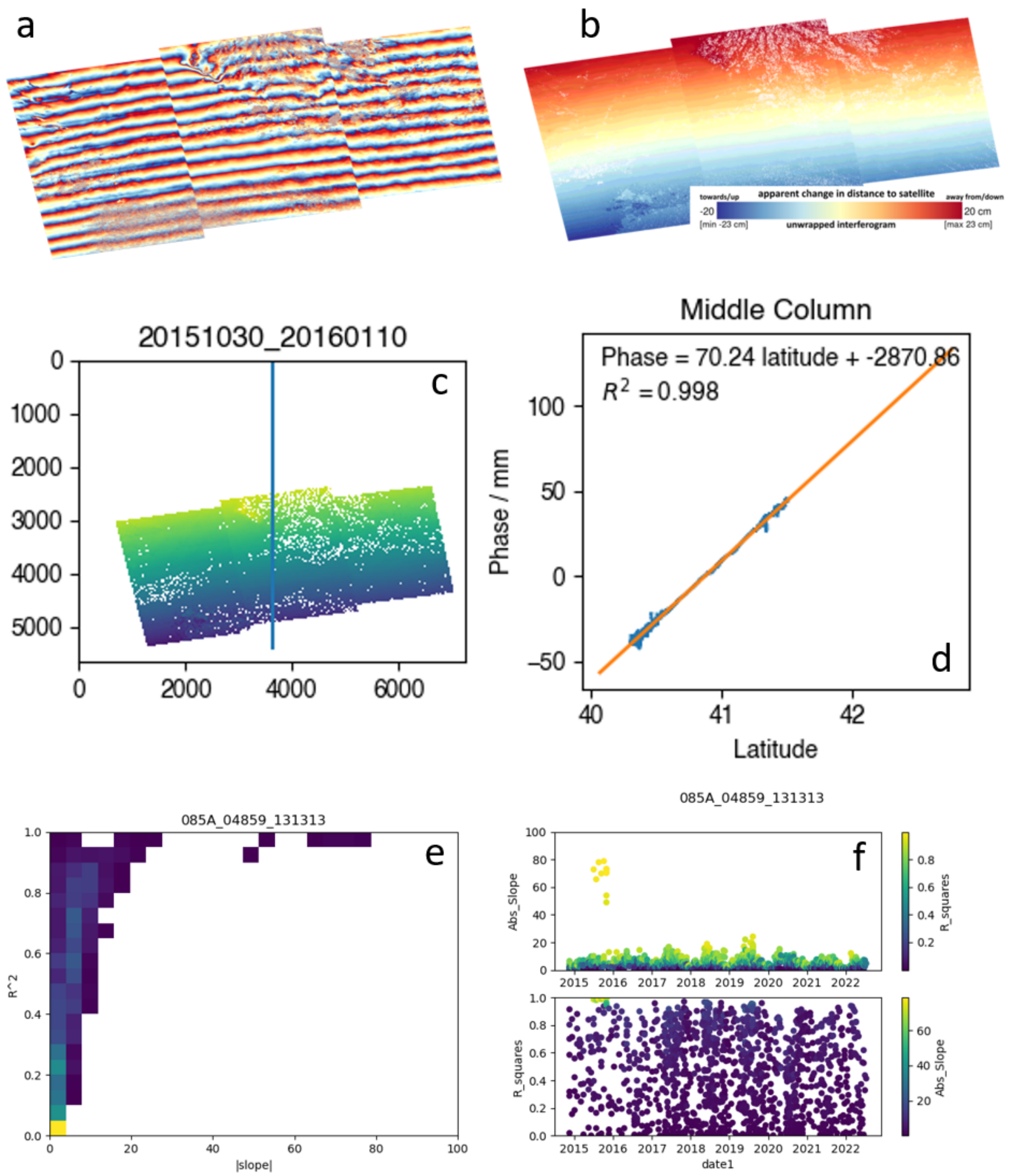

Figure S5: Illustrations of how the -s flag of the new LiCSBAS11_check_unw.py removes interferograms with coregistration error. (a) An example of a wrapped interferogram with coregistration error; (b) An example of an unwrapped interferogram with coregistration error; (c-d) A strategy of detecting this type of error by calculating the slope in mm/degree latitude and the R^2 of linear fitting from the middle column of the unwrapped interferogram. (e) Normalised 2D histogram between the absolute value of slope and the R^2 obtained from all interferograms in one frame. (f) The distribution of absolute slope and R^2 with time (the first epoch of the interferograms).

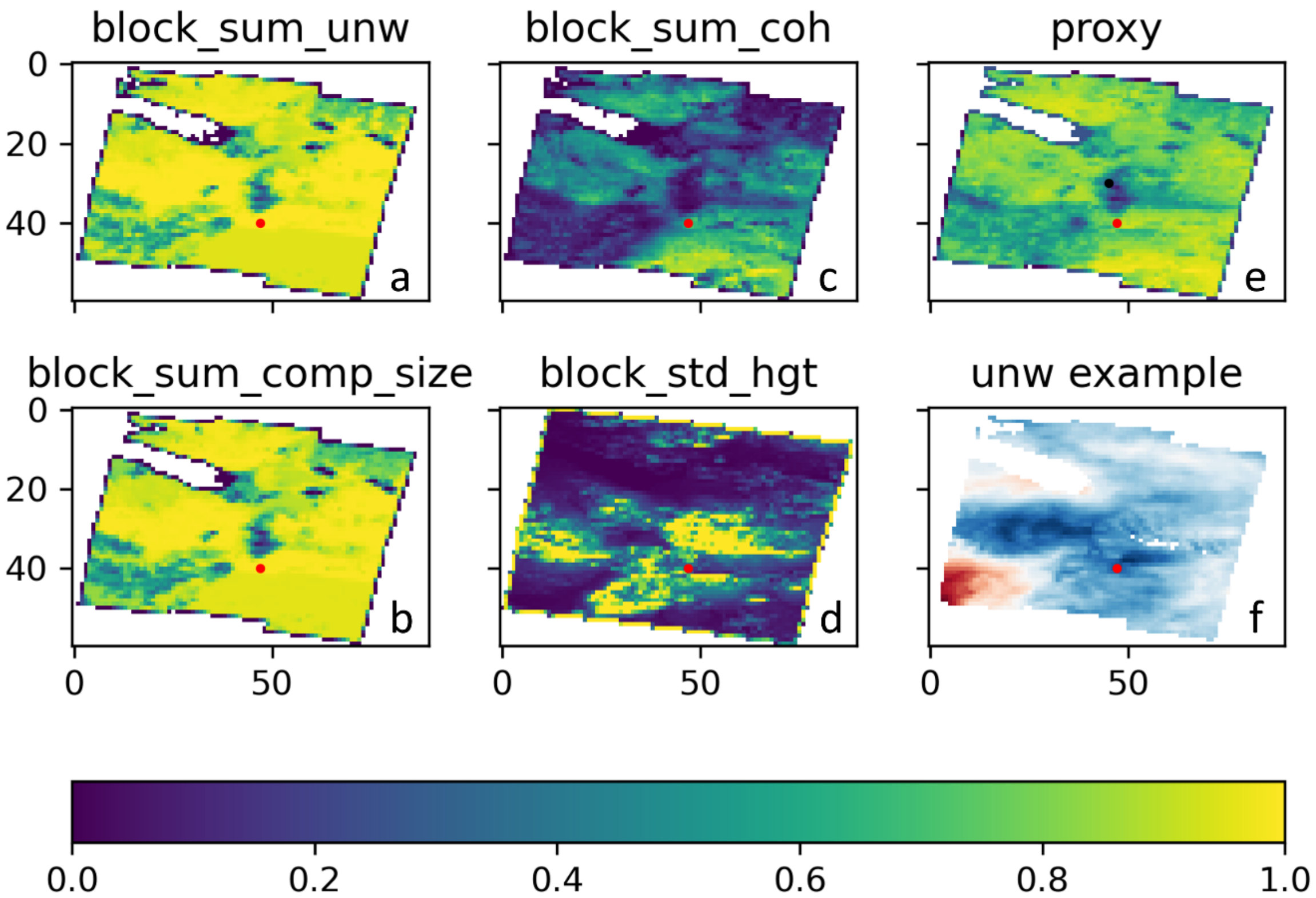

Figure S6: Reference choice based on four normalised statistical indices calculated from all interferograms in a frame. (a) Sum over interferograms of number of pixels with value in a 10 pixel x 10 pixel window (block). (b) Sum over interferograms and over the block of sizes of connected components that pixels belong to. (c) Sum over interferograms of average coherence in a block. (d) Standard deviation of elevation in a block. (e) Proxy derived from (a) + (b) + (c) – (d), normalised. (f) An example of the interferograms in the frame. Red dots show the chosen reference, which is pixel with proxy>0.9 that is the closest to the desired location in the frame, in this case, the centre of the frame as indicated by the black dot in (e).

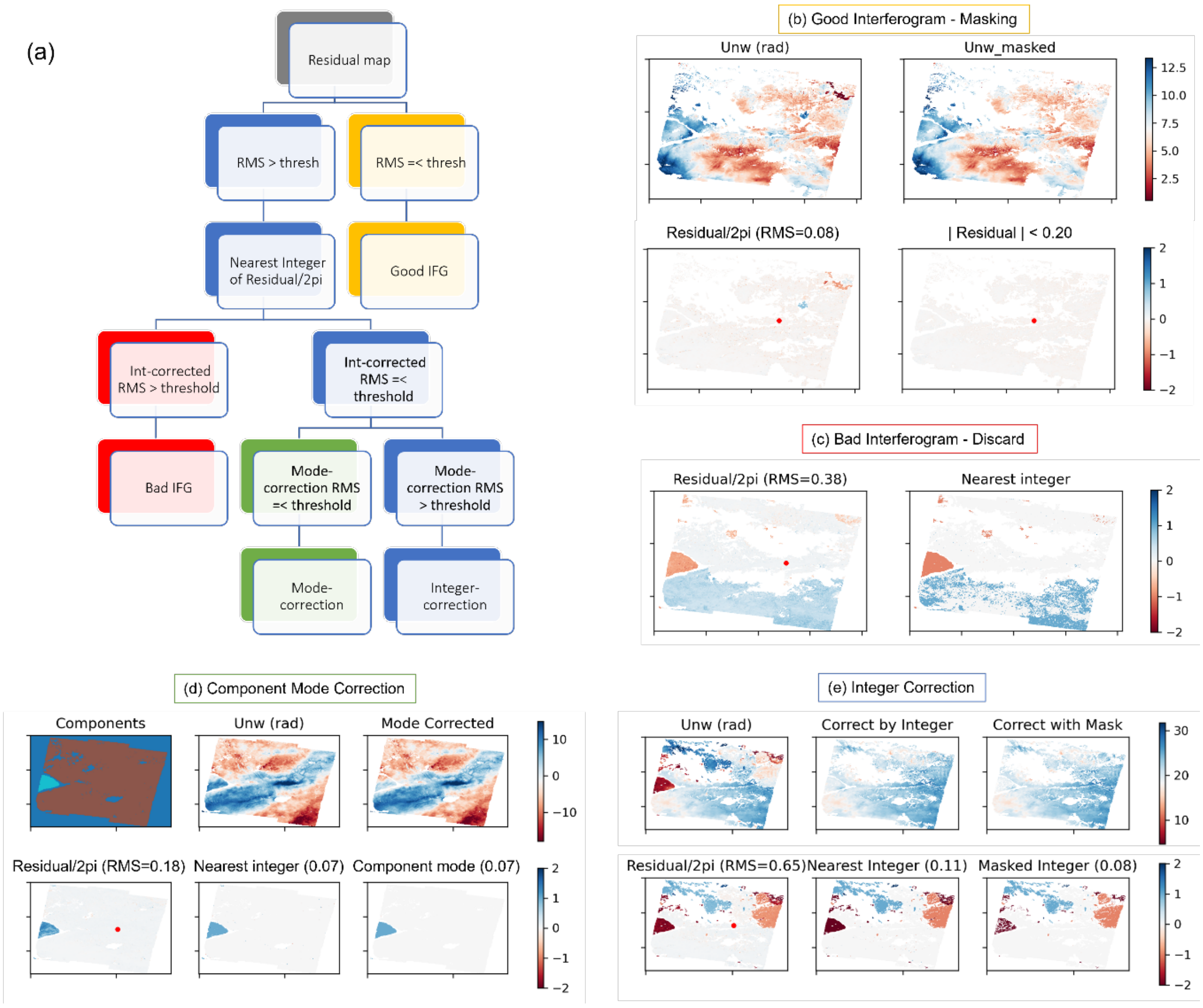

Figure S7: Workflow and examples from the new LiCSBAS132_3D_correcton.py script. (a) Decision tree implemented in LiCSBAS132_3D_correcton.py for automatic categorisation and treatment of interferograms based on the residual map from preliminary time series inversion. (b-e) show examples of four possible treatments given a threshold of 0.15 of a 2pi cycle. The numbers in brackets on the panels are root-mean-square (RMS) of the residuals divided by 2pi, or the resultant RMS residual/2pi if corrected accordingly. (b) A case of a good interferogram where the RMS residual is less than 0.15 of a 2pi, thus only pixels with absolute residual above 0.2 (hard-coded) of 2pi were masked. (c) A case of bad interferogram being discarded because subtracting the residual/2pi map by its nearest integer does not reduce the resultant RMS residual to below 0.15 of a 2pi. (d) A case where the connected components from SNAPHU helped correct the unwrapping mistake by offsetting an entire component by 2pi times the mode of the nearest integers of residual/2pi in that component. This avoids accidental pixel-level correction mistake within a connected component. (e) A case where the nearest integer map is used to correct unwrapping mistakes where the residual/2pi value is within 0.2 from a nearest integer, and pixels where the residual/2pi is over 0.2 from a nearest integer are masked instead to reduce chances of correction mistakes.

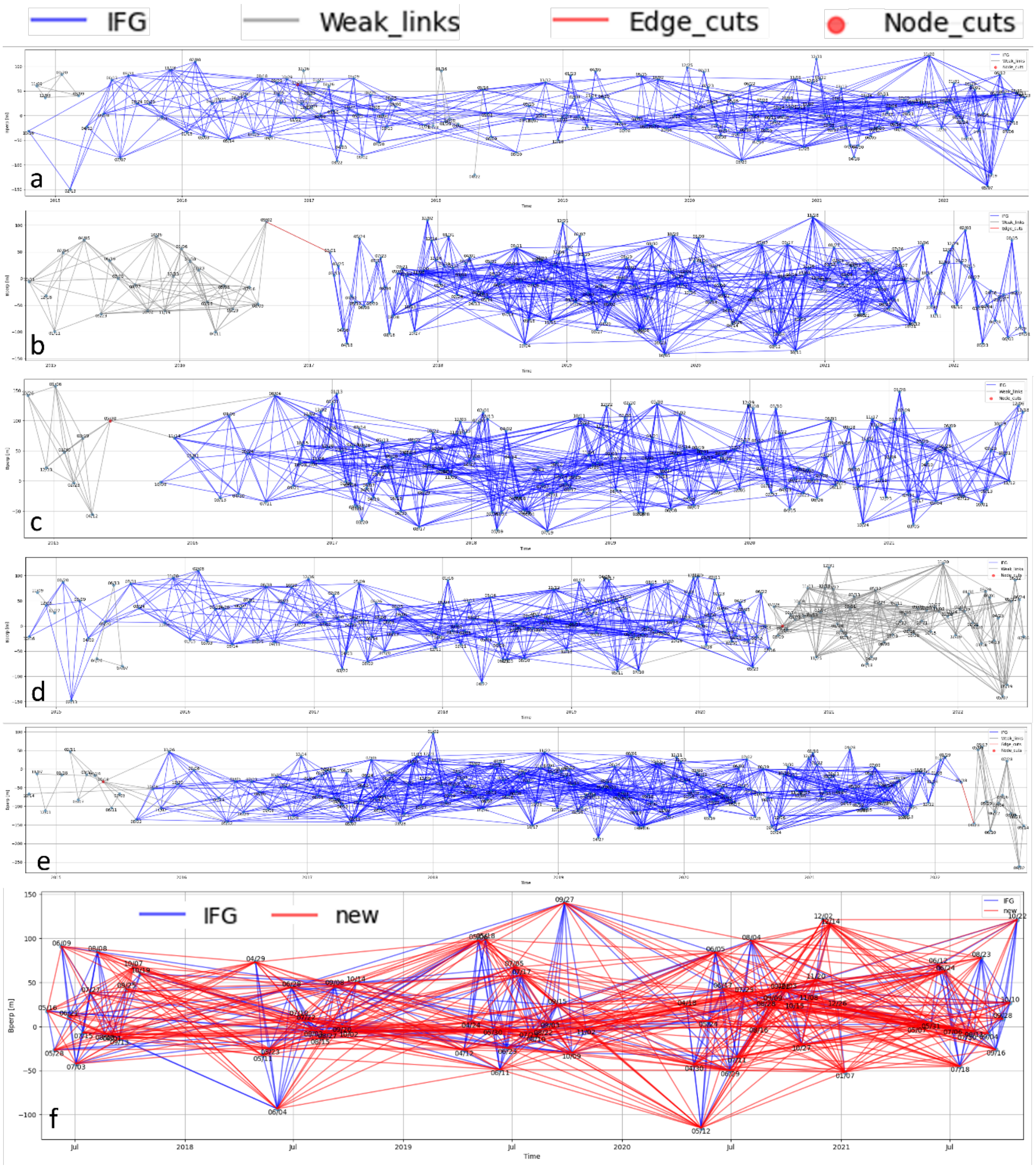

Figure S8: New features of the LiCSBAS_plot_network.py to analyse the interferogram network based on graph theory. (a) shows the separation of connected components, highlighting separate components that overlap in time, hence undetectable by temporal gap identification in the original LiCSBAS_plot_network.py, but not connected, hence not constrained, by interferograms. (b) shows an example of edge cut, which is a single interferogram, breaking which will increase the number of connected components in the network. (c) shows an example of a node cut, which is an epoch, removing which will increase the number of connected components in the network. (d-e) show examples of edge cuts and node cuts and separate components which are not obvious by visual examination. (f) show the feature of a new script called LiCSBAS_densify_ifgs.py, which generates a list of interferograms that could densify a network based on the existing list, with constraints of ranges of temporal baseline and the number of additional interferograms desired.

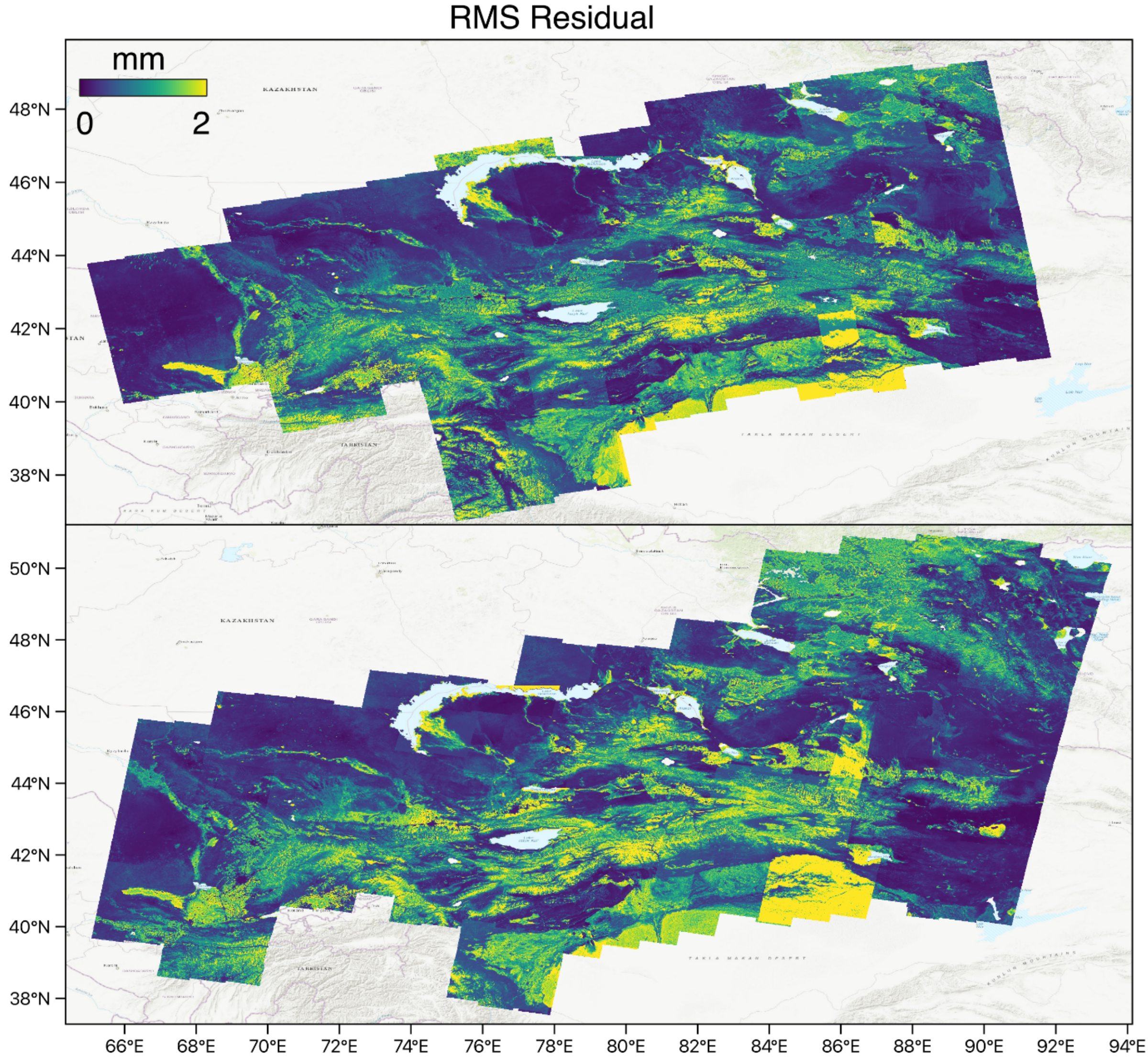

Figure S9: RMS residuals from time series inversion.

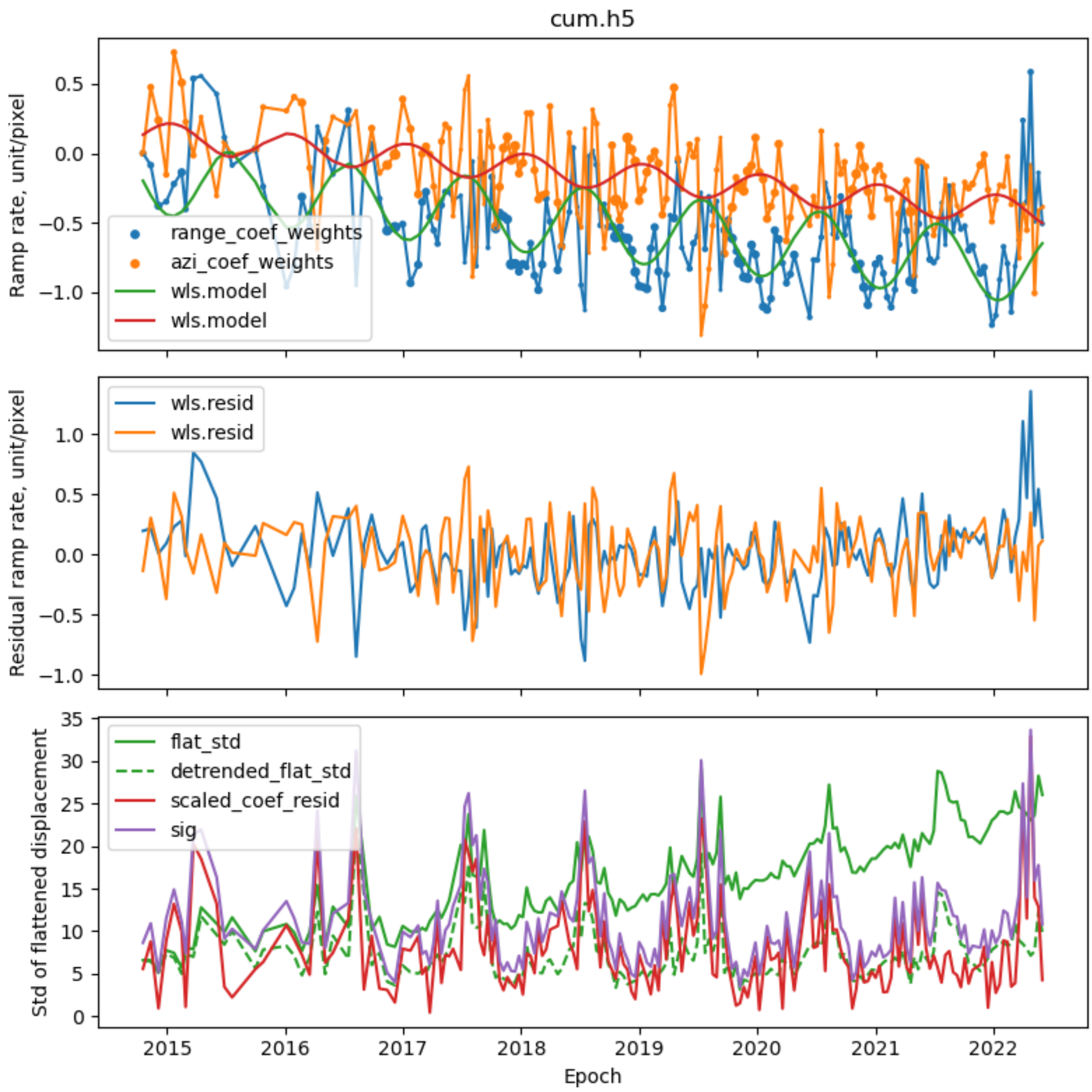

Figure S10: The improved weighting strategy for velocity inversion as implemented in the new script LiCSBAS_cum2vel_weighted.py. The sigma uncertainty used to weight the velocity inversion from the displacement time series is the purple line, which is the root-sum-square of the green dashed line and the red line (bottom panel). The green dashed line is the detrended version of the green solid line (bottom panel), which is the standard deviation of the deramped cumulative displacement map at each epoch. The red line (bottom panel) is the root-sum-square of the two curves in the middle panel, which are the range and azimuth coefficients of the ramps in the cumulative displacement map at each epoch after removing a linear and seasonal model (top panel) fitted through least squares inversion weighted by the green dashed line in the bottom panel.

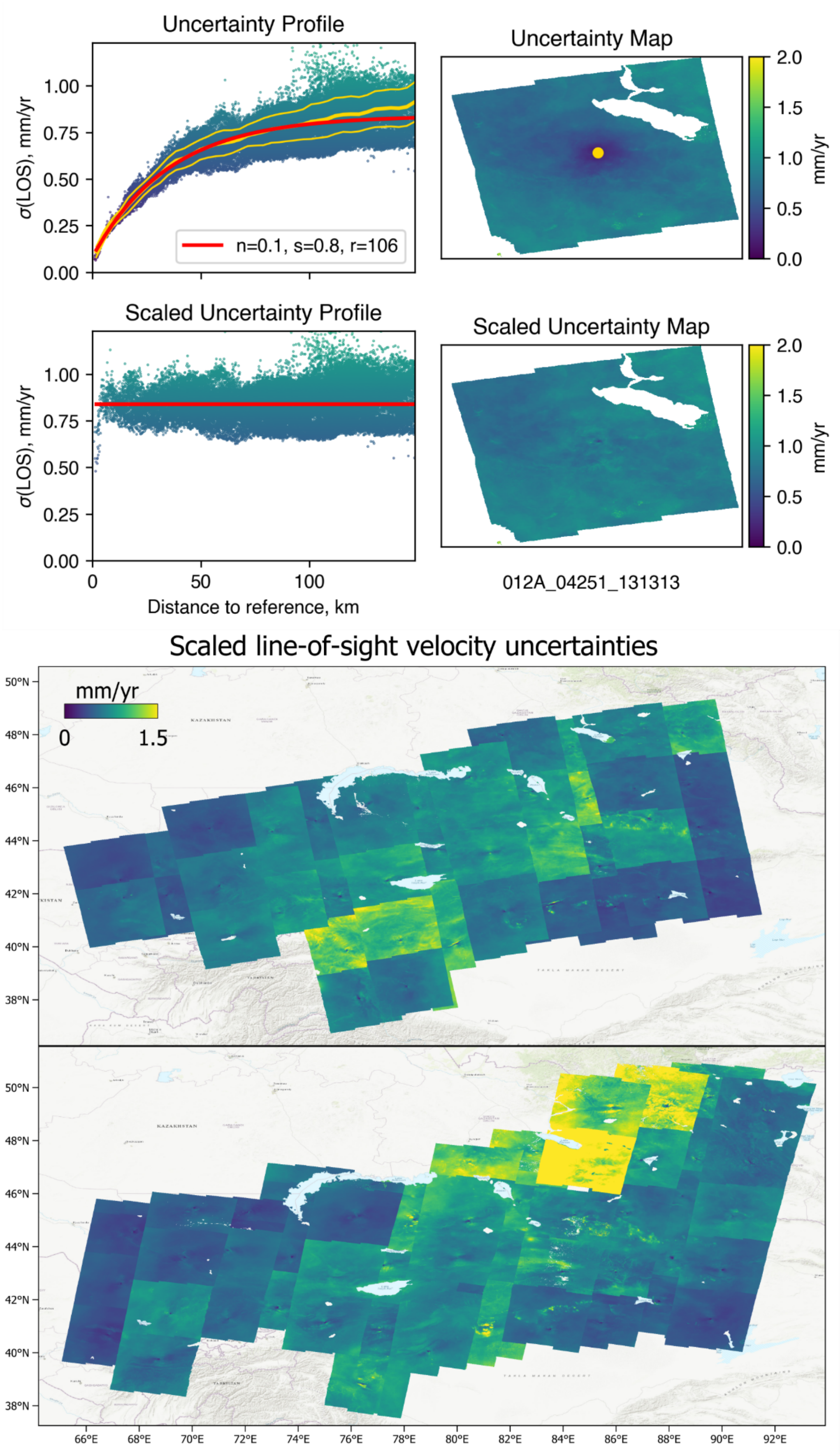

Figure S11: Uncertainties of line-of-sight velocities derived from the weighted velocity inversion, and after a correction from the reference effect (Ou et al., 2022).

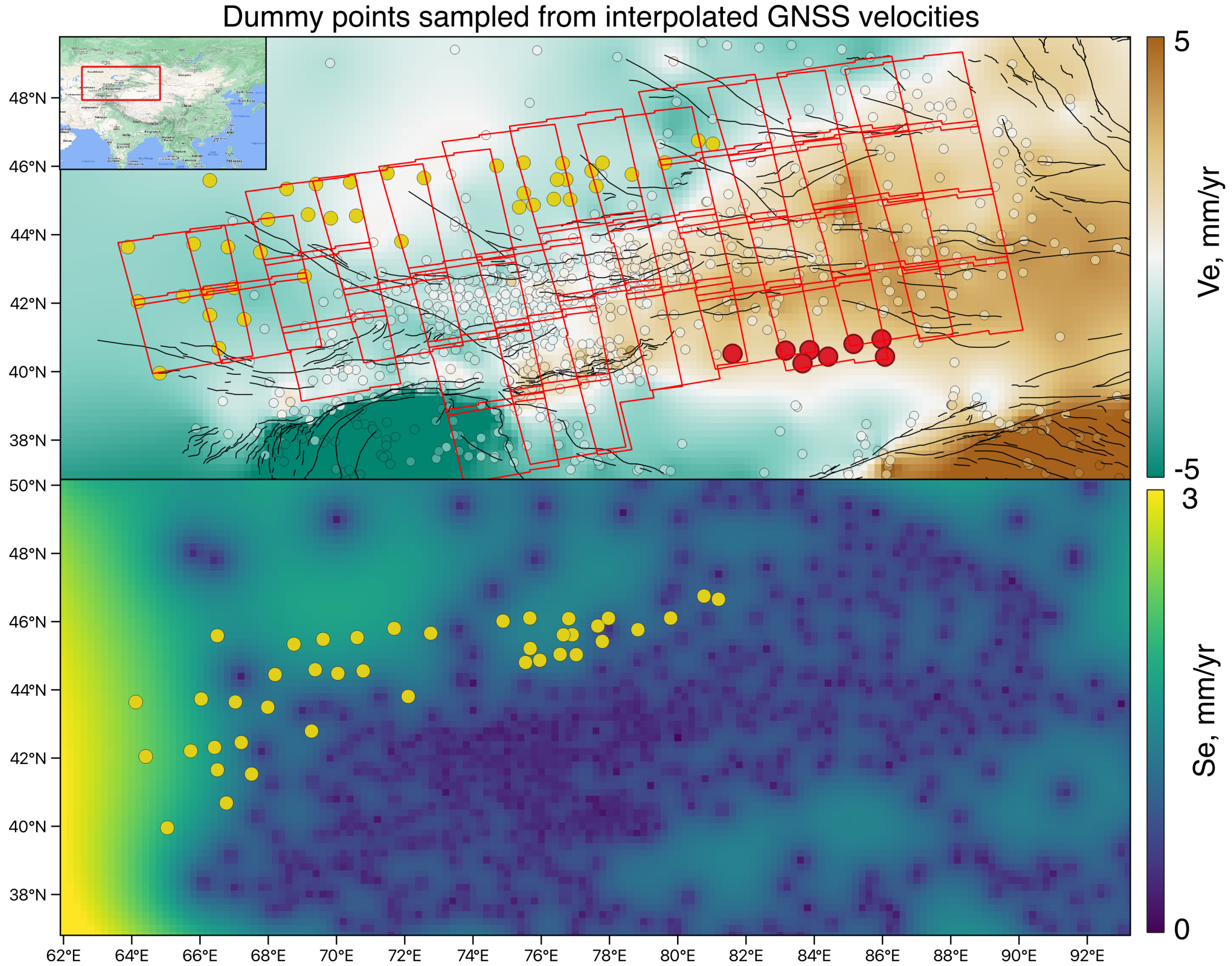

Figure S12: Complementing the compiled published GNSS velocities in small coloured circles using dummy GNSS in the north sampled from interpolated GNSS velocities and uncertainties (yellow dots), and in the south calculated from the block rotation of the Tarim Basin (red dots).

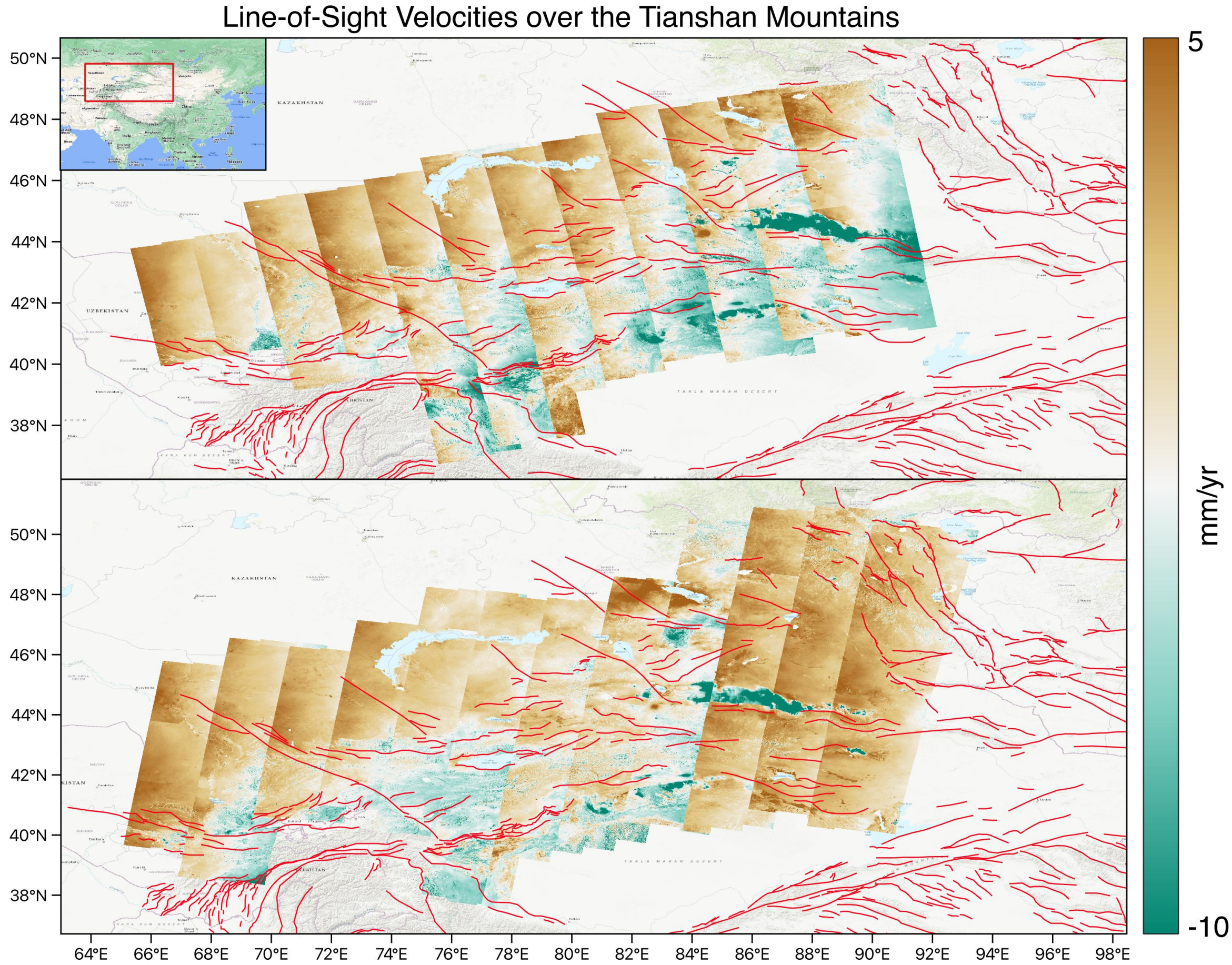

*Figure S13: Line-of-sight velocities derived from velocity inversion weighted using improved strategy (Text S6 and Figure S10), and concatenated along track using constant offsets based on frame overlaps. However, the general trend along track is still different from that of the plate motion (Figure S14**Error! Reference source not found.**), possibly due to long-wavelength s ignals from the ionospheric effect (Stephenson et al., 2022).*

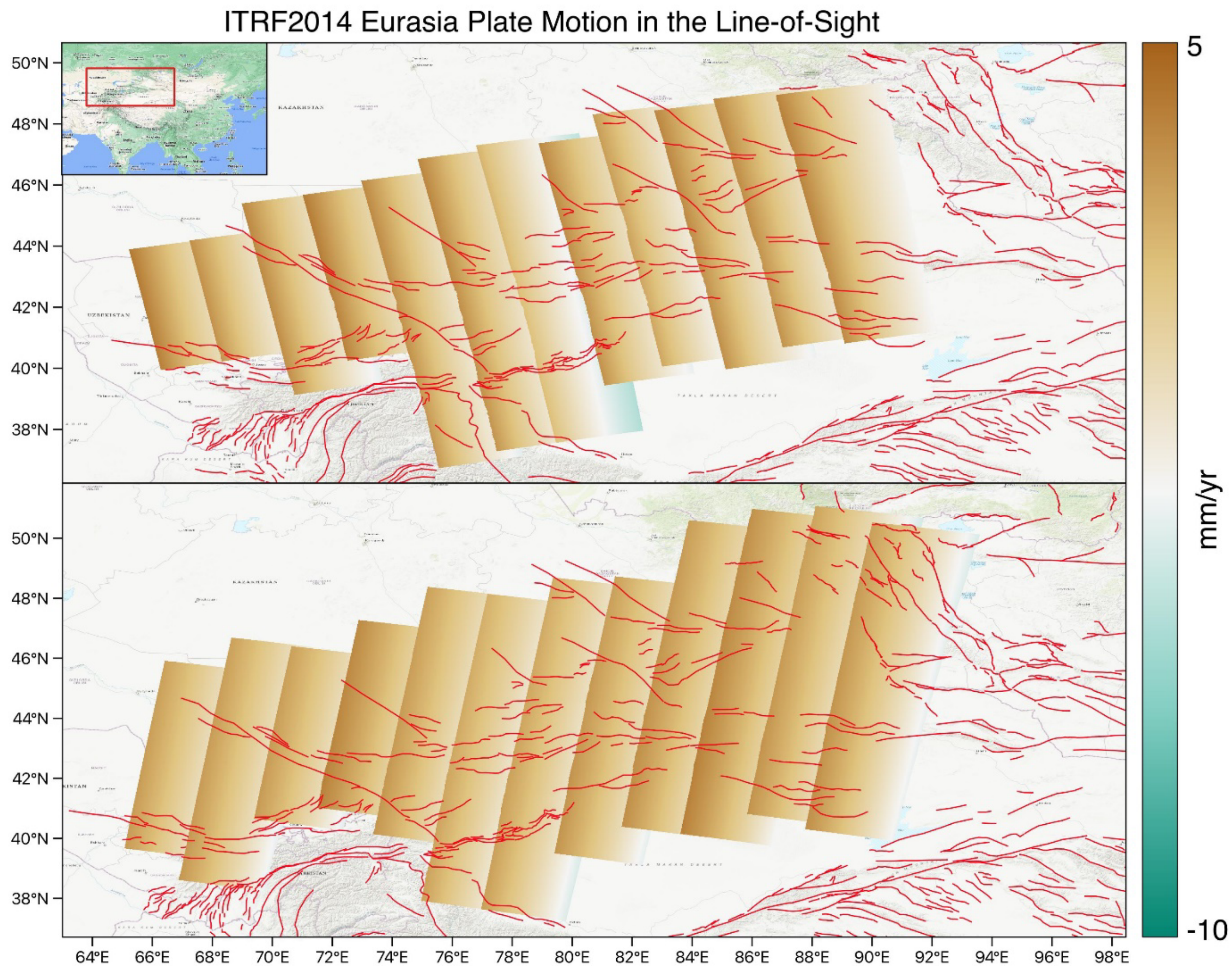

Figure S14: Line-of-sight velocity from plate motion.

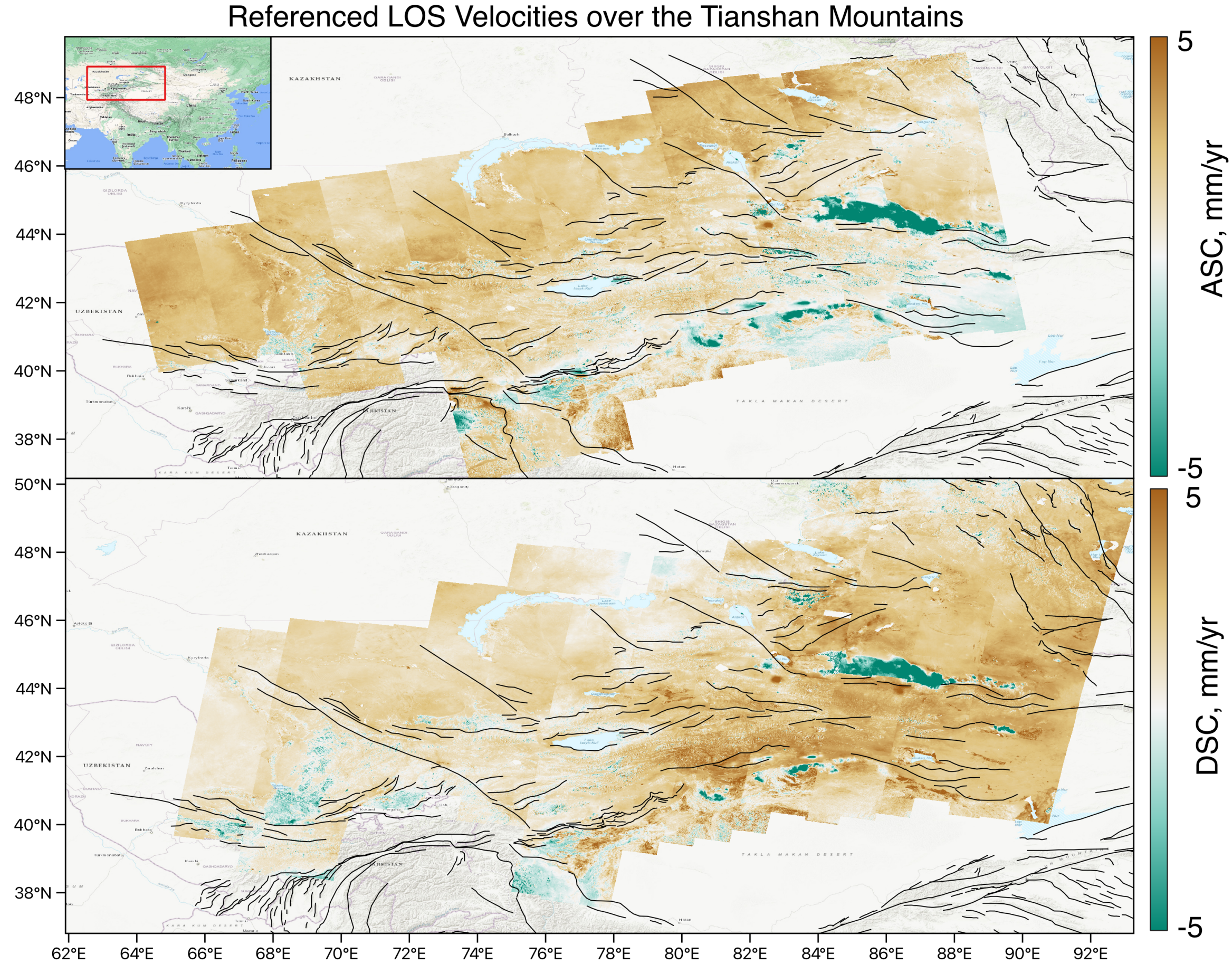

Figure S15: Line-of-sight velocities after referencing to the GNSS velocities including the dummy controlled velocities in Figure S12.

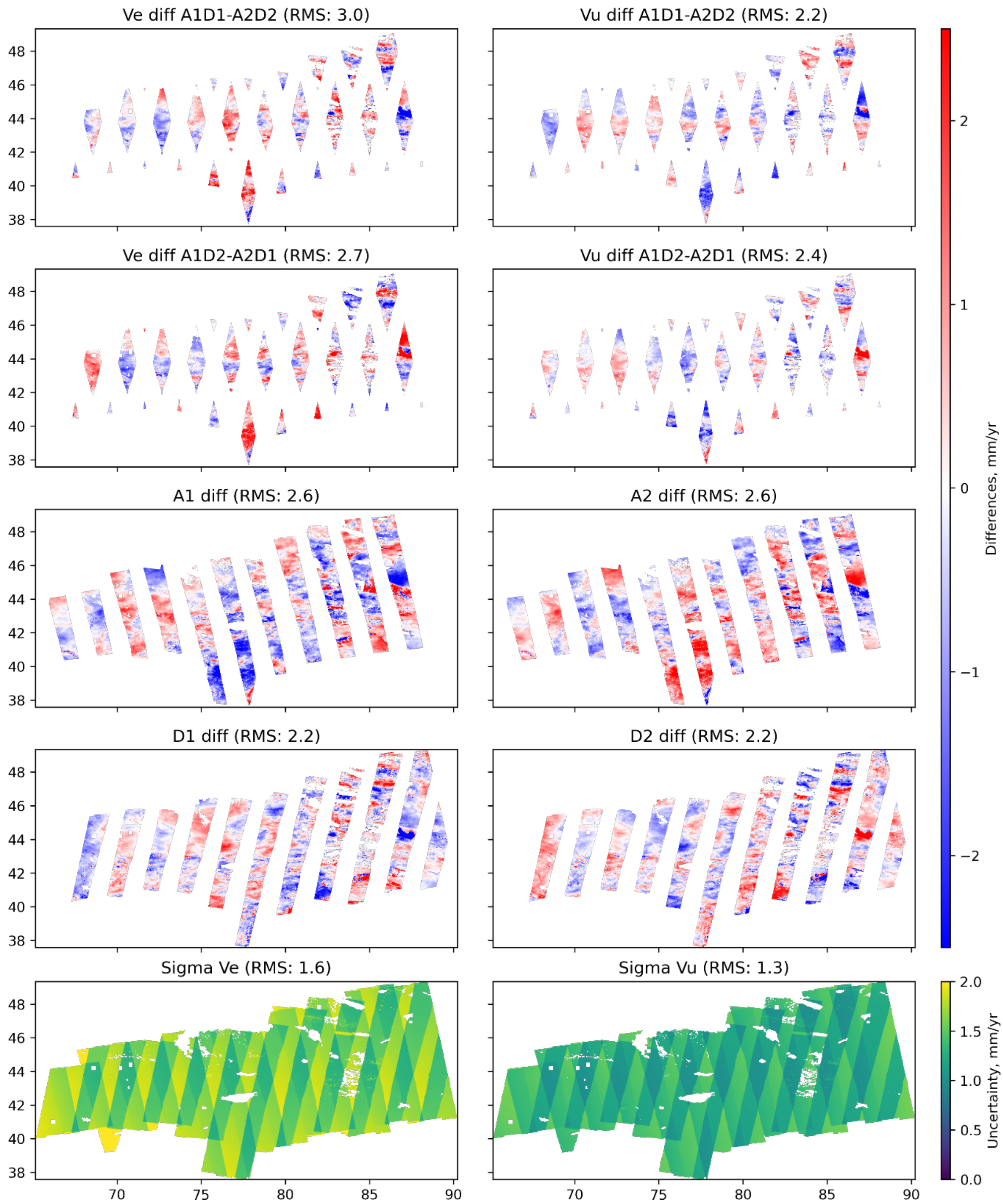

Figure S16: Uncertainty estimations. (top 4 panels) differences between two independent sets of east and vertical velocities decomposed from two combinations of independent ascending and descending line-of-sight velocities at the diamond shapes overlapped by 4 tracks. (middle 4 panels) differences between observed line-of-sight velocities (in Figure S15) and line-of-sight velocities predicted from east and vertical velocities decomposed from independent ascending and descending line-of-sight velocities at stripes overlapped by at least 3 tracks. These RMS differences yield 1.9 mm/yr and 1.2 mm/yr as average uncertainties of observed ascending and descending velocities. Propagating them through velocity decomposition gives pixel-wise east and vertical velocity uncertainties (bottom two panels).

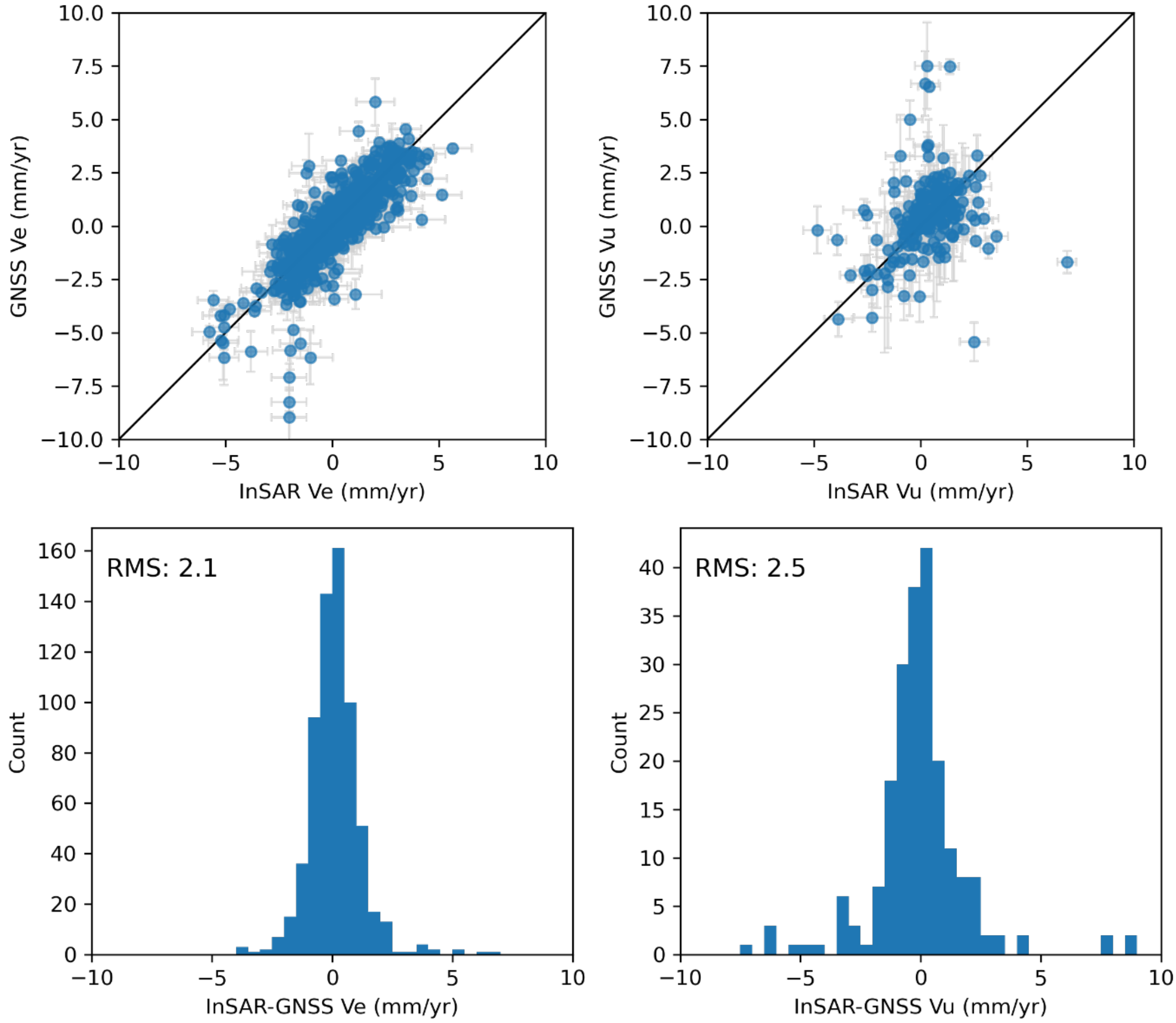

Figure S17: Comparison between InSAR and GNSS east and vertical velocities.

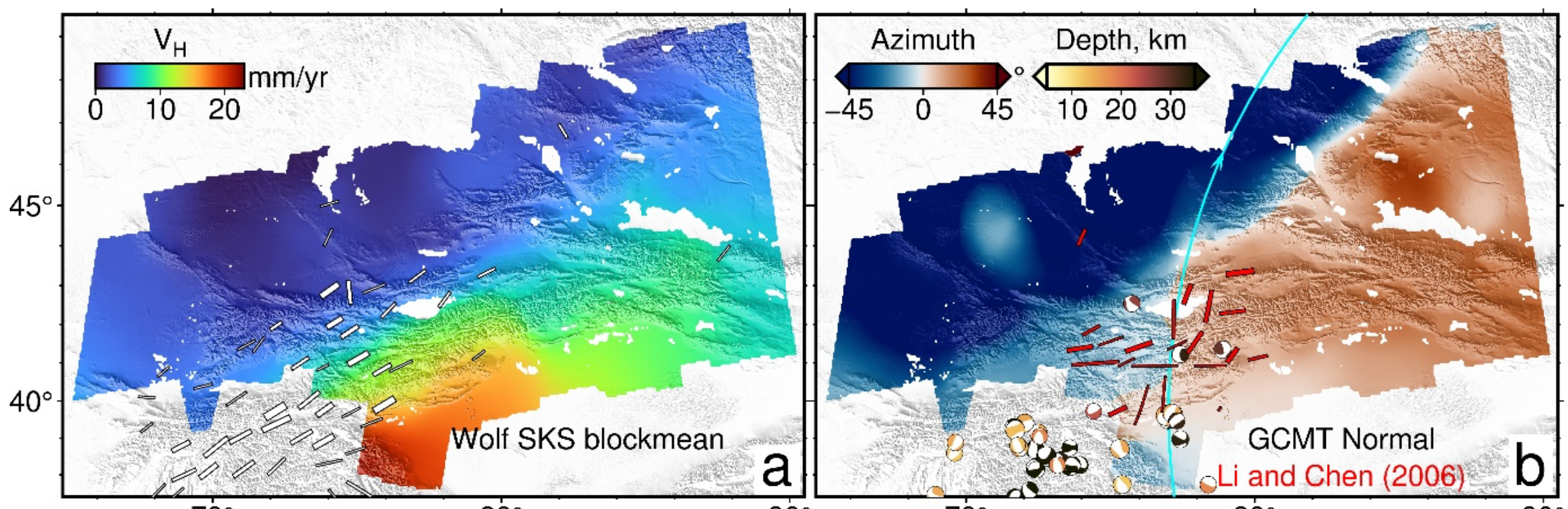

Figure S18: Magnitudes and azimuths of horizontal velocities. (a) Horizontal velocity and (b) azimuth of the horizontal velocity derived from filtered east and north velocities in Figure 2a,b. (a) reproduces the block mean SKS fast axes from Wolf et al., (2025). (b) contains shear wave splitting results from Li and Chen, (2006), GCMT normal faulting earthquakes (both tension and null axe have plunges <45 degrees) (Ekström et al., 2012), and arc (cyan) in the sense of Tarim's rotation (Wang et al., 2020). The gap between the cyan arc and the wedge with positive azimuths in the northeast corner is a result of the effective shortening and eastward escape of Tian Shan crustal material through a hierarchical system of conjugate strike slip motion on the basin-bounding faults that form east-opening wedges.

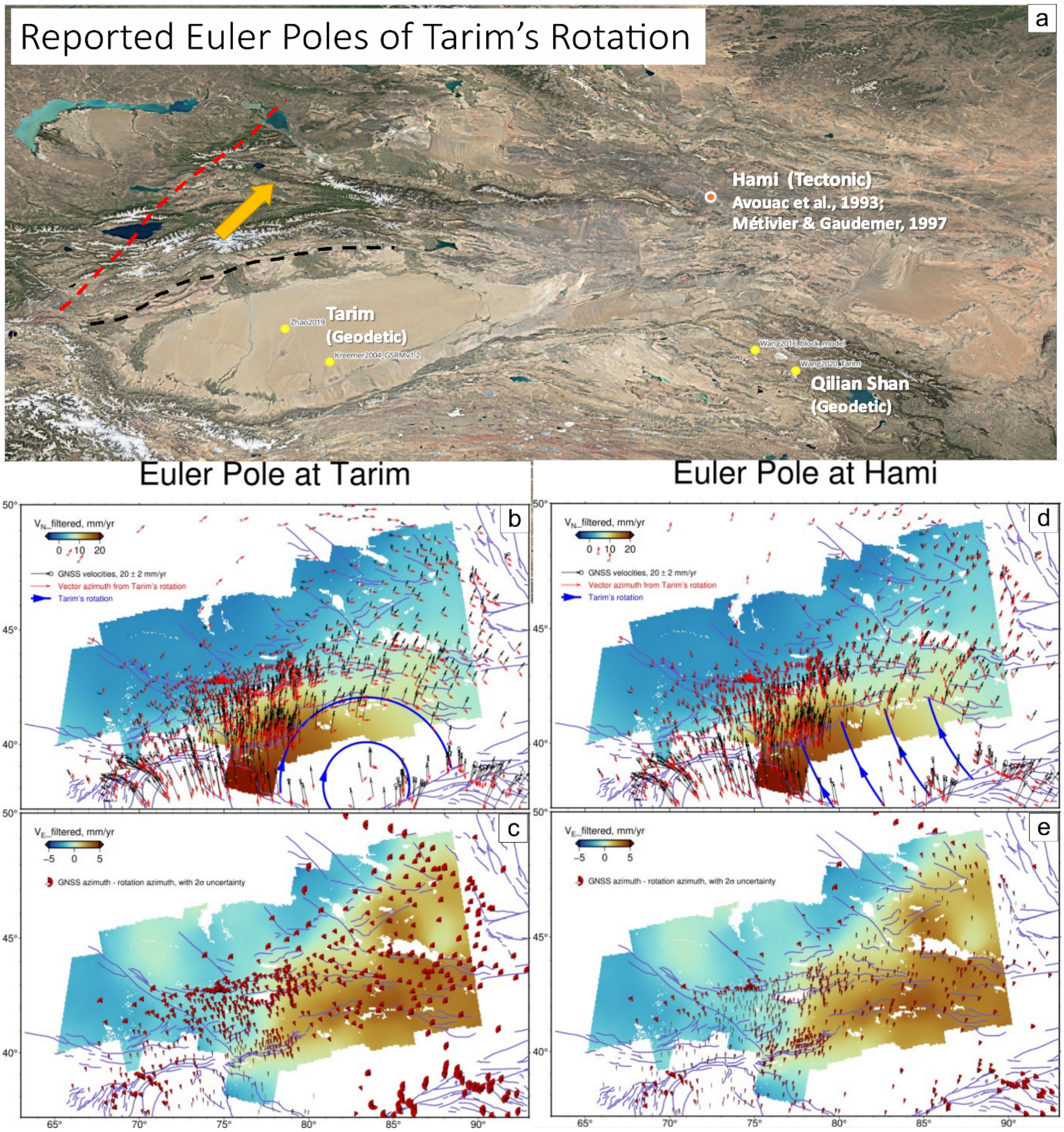

Figure S19: Reported Euler Poles of Tarim’s rotation and the fits to GNSS vectors. (a) All Euler Poles found in literature. (b-c) Azimuth comparison between GNSS velocities and unit vectors given Tarim’s Euler Pole within Tarim. (d-e) Azimuth comparison between GNSS velocities and unit vectors given Tarim’s Euler Pole at Hami. The uncertainty wedges for the azimuth misfits only take into account the uncertainties of GNSS velocities. The contribution from uncertainties in Euler Pole locations (between two estimates within the Tarim, or between two estimates within the Qilian Shan) are negligible (6 ± 10 degrees).

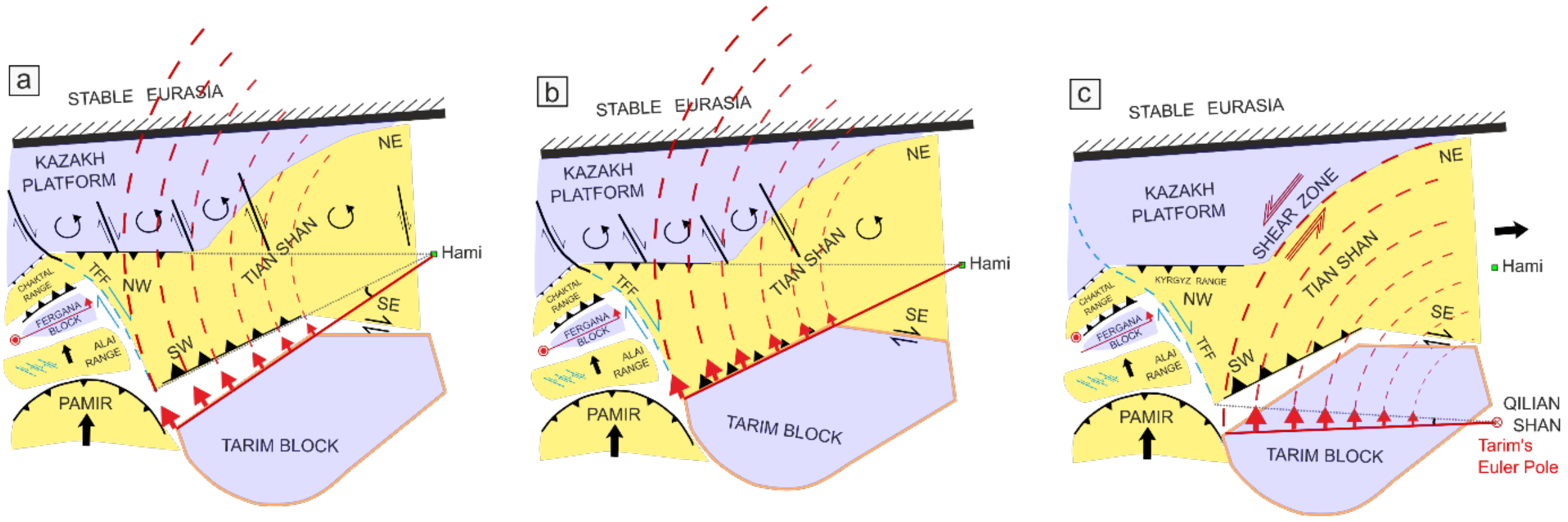

Figure S20: Cartoons models of reconstructed stress field given Tarim's rotation. (a) stress field before Tarim's 7 degrees of clockwise rotation with an Euler Pole around Hami, which promotes the parallel strike-slip faulting in the Kazakh Platform; (b) stress field today if the Euler Pole is still around Hami, but with the NW-trending strike-slip faults rotated anticlockwise relative to (a), about to be out of optimal orientation; (c) stress field at the onset of Tarim's rotation if the Euler Pole had always been in the Qilian Shan, in which case the NW-trending platform-cutting strike-slip faults might not have formed, and the NE-trending shear zone after rotation could have been more mature than it is today.
