# Supplementary material for: Extension of Tian Shan along a nascent shear zone
Source: Nat Commun. 2026 Jul 22;17:8943. doi: 10.1038/s41467-026-74965-2 (PMC13503760; doi:10.1038/s41467-026-74965-2)
Supplement: Supplementary file 2 [file 41467_2026_74965_MOESM2_ESM.pdf]

nature portfolio

Peer Review File

Extension of Tian Shan along a nascent shear zone

Corresponding Author: Dr Qi Ou

Parts of this Peer Review File have been redacted as indicated to remove third-party material.

This file contains all reviewer reports in order by version, followed by all author rebuttals in order by version.

Attachments originally included by the reviewers as part of their assessment can be found at the end of this file.

Version 0:

Reviewer comments:

Reviewer #1

(Remarks to the Author)

(Remarks on code availability)
I have used this code before, but have not checked the improvements described in the supporting material in detail. The improvements, however, sound very helpful and reasonable.
The older version of the software is easy to install and use, comes with test cases, manuals, READMEs etc. The code is open and can be modified to one's own liking.
I would appreciate if the authors make an effort to also provide an updated manual and check whether the LiCSAR data base contains all necessary metafiles to run the code without error (I, for example, often run into problems with the baseline information for some reason).

Reviewer #2

(Remarks to the Author)
This manuscript presents a compelling study of the active deformation mechanisms within the Tianshan orogenic belt. Based on dataset of Sentinel-1 InSAR and GNSS velocities, this study revealed a previously unrecognized, through-going northeast-trending shear zone in the entire Tianshan in response to the rotation of the Tarim basin. The key finding provides a novel framework for understanding the intracontinental evolution of this orogen. The manuscript is well-written, and the conclusions are well-supported by the data. However, several points require clarification to strengthen the arguments and enhance the manuscript's clarity and impact before consideration of publication.

(1) The term "nascent shear zone" is central to the manuscript. While the geodetic and seismological evidence for distributed shear is convincing, direct evidence for its incipient nature is somewhat circumstantial. The authors should strengthen this argument by explicitly discussing what geological or geomorphological evidence would be expected for a newly developing shear zone. Besides, further elaborating on the comparison to other nascent fault systems is beneficial to clarifying if the current strain distribution and fault segmentation patterns of the research area are diagnostic of an early evolutionary stage.

(2) The manuscript convincingly links the deformation to the rotation of the Tarim Basin around a Euler pole to its southeast. However, the underlying driving forces for this specific rotational kinematics could be discussed in greater depth. A more detailed discussion on how this rotation fits into the broader context of the India-Eurasia collision, potentially relating to the indentation of the Pamir/India or large-scale mantle flow, would provide a more complete dynamical picture.

(3) The proposed "hierarchical wedge model" is interesting, but its advantages and limitations could be more explicitly contrasted with existing models (e.g., the "bookshelf model"). Please more clearly state which specific observations the new model explains better than previous ones. In addition, a brief discussion on the model's limitations would strengthen the discussion.

(4) The identification of non-tectonic signals (permafrost creep, glacial isostatic adjustment, groundwater extraction) is a

strength. However, the methodology for quantitatively separating these signals from the tectonic background is not fully detailed.

(5) For the Introduction section, although it effectively sets the scene but could be more explicitly state the key knowledge gap and the specific contribution of this study in the final paragraph.

(Remarks on code availability)

Reviewer #3

(Remarks to the Author)
This paper presents a comprehensive and high-resolution analysis of crustal deformation across the Tian Shan orogenic belt, combining an extensive dataset of Sentinel-1 InSAR interferograms. The study makes several key contributions: 1) High-resolution deformation maps: the authors derive the east-west and vertical velocity fields, and strain-rate fields covering over 2 million km² of the Tian Shan. This represents a significant advancement in spatial resolution and coverage compared to previous studies. 2) Based on large-scale Earth observations from space, this paper proposes a novel explanation: a NE-trending, range-cutting shear zone driven by the rotation of the Tarim Basin. 3) The authors propose a kinematic model involving east-opening triangular wedges at multiple scales to explain the eastward crustal flow and extensional deformation in the south-eastern Tian Shan. This model offers a fresh perspective on how deformation is accommodated in the region.

The InSAR big-data processing is supported by the Looking into Continent from Space (LiCS) system of the COMET team. The data-processing strategy is robust, and I believe the results are solid. The article's logic is clear and its structure is sound.

Minor Suggestions for Revision:

1) While the velocity gradients, vorticity and strain-rate fields strongly suggest the presence of a shear zone, the authors should more explicitly discuss the limitations or alternative interpretations of this feature. For example, could the signal be influenced by deeper crustal flow without a clear surface expression?

2) The InSAR velocity results are sensitive to errors such as atmospheric delay, especially for wide-area processing. A brief description of how these were mitigated, and an estimate of the remaining uncertainties, would strengthen the interpretation of vertical motions.

3) The Hierarchical Wedge Model is innovative but could benefit from a more detailed comparison with existing models (e.g., bookshelf faulting) to better highlight its advantages and limitations.

(Remarks on code availability)
LiCSBAS is a time-series InSAR analysis tool widely used by the InSAR community. My team uses it in their research, so I can confirm its usability.

Version 1:

Reviewer comments:

Reviewer #1

(Remarks to the Author)
Dear author team,
I want to thank and congratulate you for this careful review. I have nothing to add to my previous comments and look forward to see this work published.
With warm regards,
Sabrina Metzger

(Remarks on code availability)
All three software links provided in the data availability section are correct and useful. The link to the data (rate and strain maps in high resolution) is missing. Please make sure to provide your valuable maps to the public.

Reviewer #2

(Remarks to the Author)
This manuscript presents a comprehensive geodetic analysis of deformation across the Tian Shan, integrating large-scale Sentinel-1 InSAR and GNSS data. The identification of a NE-trending, range-scale distributed shear zone and its linkage to the rotation of the Tarim Basin represents a potentially important contribution to understanding intracontinental deformation. The authors have responded carefully to the previous comments, and the manuscript has been significantly improved in clarity, structure, and geodynamic context. I believe it is now suitable for publication after minor revision.

A few points could still be clarified to further strengthen the manuscript. First, the interpretation of a “nascent shear zone” is now better justified, but remains somewhat inferential. It would be helpful to more explicitly state what observational criteria uniquely support an incipient shear zone (as opposed to long-lived distributed deformation).

Second, the discussion of the driving mechanism related to Tarim rotation and Euler pole migration is much improved; however, it would benefit from a clearer statement of the level of constraint (inferred vs. demonstrated) and a brief acknowledgment of uncertainties in the proposed mechanism within the broader India–Eurasia collision framework.

Third, the manuscript would benefit from a brief clarification of how these signals are distinguished from tectonic deformation in practice (e.g., spatial scales or filtering considerations), to reassure readers of the robustness of the tectonic interpretations.

These are relatively minor clarifications and do not require additional analyses.

(Remarks on code availability)

Open Access This Peer Review File is licensed under a Creative Commons Attribution 4.0 International License, which permits use, sharing, adaptation, distribution and reproduction in any medium or format, as long as you give appropriate credit to the original author(s) and the source, provide a link to the Creative Commons license, and indicate if changes were made.
In cases where reviewers are anonymous, credit should be given to 'Anonymous Referee' and the source.
The images or other third party material in this Peer Review File are included in the article’s Creative Commons license, unless indicated otherwise in a credit line to the material. If material is not included in the article’s Creative Commons license and your intended use is not permitted by statutory regulation or exceeds the permitted use, you will need to obtain permission directly from the copyright holder.
To view a copy of this license, visit https://creativecommons.org/licenses/by/4.0/

Thank you to both you and the reviewers, for handling our manuscript and for offering us the chance to revise it following the reviewers' constructive comments.

We are grateful to the three reviewers for their detailed comments. The first reviewer, in particular, had several great suggestions both in improving the presentation, and also how we addressed the science and implications of our result. Cumulatively, these have all led to a much better testing of the ideas.

We have highlighted below our responses in italics to each reviewer comment line-by-line and with the new line numbers in the updated manuscript. We have also provided a track changes document highlighting the differences (red strikeout removal of text, cyan blue new insertion).

I hope that the revised manuscript is much improved in communicating the results of our research and thank you for considering these changes.

Yours sincerely,

Qi Ou
On behalf of all the co-authors.

Reviewer #1 (Remarks to the Author):

Summary

Ou et al. present the first InSAR-derived surface deformation maps of the full Tian Shan mountain range. Using a dense GNSS velocity field compiled from various sources the authors projected 90 individual InSAR rate maps into a European-fixed reference frame. They found a broad shear zone along a northeastern axis, suggesting that the Tian Shan is extending along the strike of that shear zone, which they explain with the northward indentation of the Tarim basin. These findings are backed up with published seismic observations from shear wave splitting and earthquake focal mechanisms. In addition they show examples of localized fault activity, as well as climatic and anthropogenic processes related to ice loss, respectively, water pumping as it has already been observed elsewhere using the same methods.
The author group unites high experience in creating large-scale InSAR products from other orogens and they are constantly refining their strategy of InSAR data processing and time-series analysis to improve the signal-to-noise ratio and produce highly informative surface deformation rate maps, so I have nothing to criticize on the methods. The conclusions drawn from their observations are reasonable. The language is clear and concise, the figures are informative, if somewhat dense or small.
I therefore have only minor questions and suggestions to improve the clarity of the manuscript, see below. Not all my suggestions must be considered, but I think the **first two key points in particular** would increase the impact of the work for tectonic experts if acknowledged, as they will help to better understand (1) the tectonic evolution of the Tian Shan over time and (2) how large strike-slip systems worldwide might form.
I hope the authors find my comments useful.
Sabrina Metzger, 23.10.2025

Thank you for your very detailed review, your recognition of our improved InSAR methodology, as well as your valuable and extension number of suggestions for increasing the impact of our manuscript.
We have found your comments particularly useful and have taken on board your key points, paying particular attention to the first two as you ask, in improving the communication of the tectonic implications.

Key points

• **The role of Talas Fergana fault (and others)** You introduce the (role and presence of) NW-trending large fault systems at the northwestern margin of the Tian Shan (Talas Fergana fault, AkF, DzF, ChF) very late in the text and I did not understand how you explain their unfavorable geometry w.r.t. the kinematics of the large shear zone. Along those lines I suggest to better introduce and later discuss the temporal evolution of the Tian Shan. Are there paleomagnetic records or other geologic data explaining how/when the Tian Shan subranges and dextral large faults systems have been built?

Thank you for your suggestions. We have now dedicated the second half of the third paragraph of the Introduction (Lines 105-121) to properly introduce the NW-trending large fault systems at the northwestern margin of the Tian Shan. Follow your suggestion, we proposed a model of the temporal evolution of the Tian Shan based on geological evidence about the timings of reactivation of the Tian Shan, indentation of the Pamir and the Indian-Tarim collision at around 10 Ma. We now suggest that the Indian-Tarim collision might have caused a southward shift of Tarim's Euler Pole from near Hami to the interior of the Qilian Shan (Section 2.3, Lines 796-926). Under the previous stress field, the northward compression in the Kazakh Platform promoted the bookshelf rotation between the NW-trending large fault system. The current stress field from Tarim's rotation almost bypasses the NW-trending faults, instead favours the generation of a new NE-trended shear zone and the eastward crustal escape in the southeast Tian Shan. Paleomagnetic records are only found on the edges of the Tian Shan range (Fig.3a, reproduced below), and were interpreted as recording local rotations, which favours our new hierarchical wedge model. Due to the complex tectonic history in this region, the Tian Shan subranges and the dextral large fault systems were built since Paleozoic. We introduce the Paleozoic and Mesozoic tectonic events in the second paragraph of the Introduction and present the 4 Paleozoic suture zones in Figure 2i. We found the suture zones important for explaining Cherie et al (2016)'s 2-layer shear wave splitting data, and the rapid uplift we observe in the southern Tian Shan (Fig.5e, Lines 1120-1153).

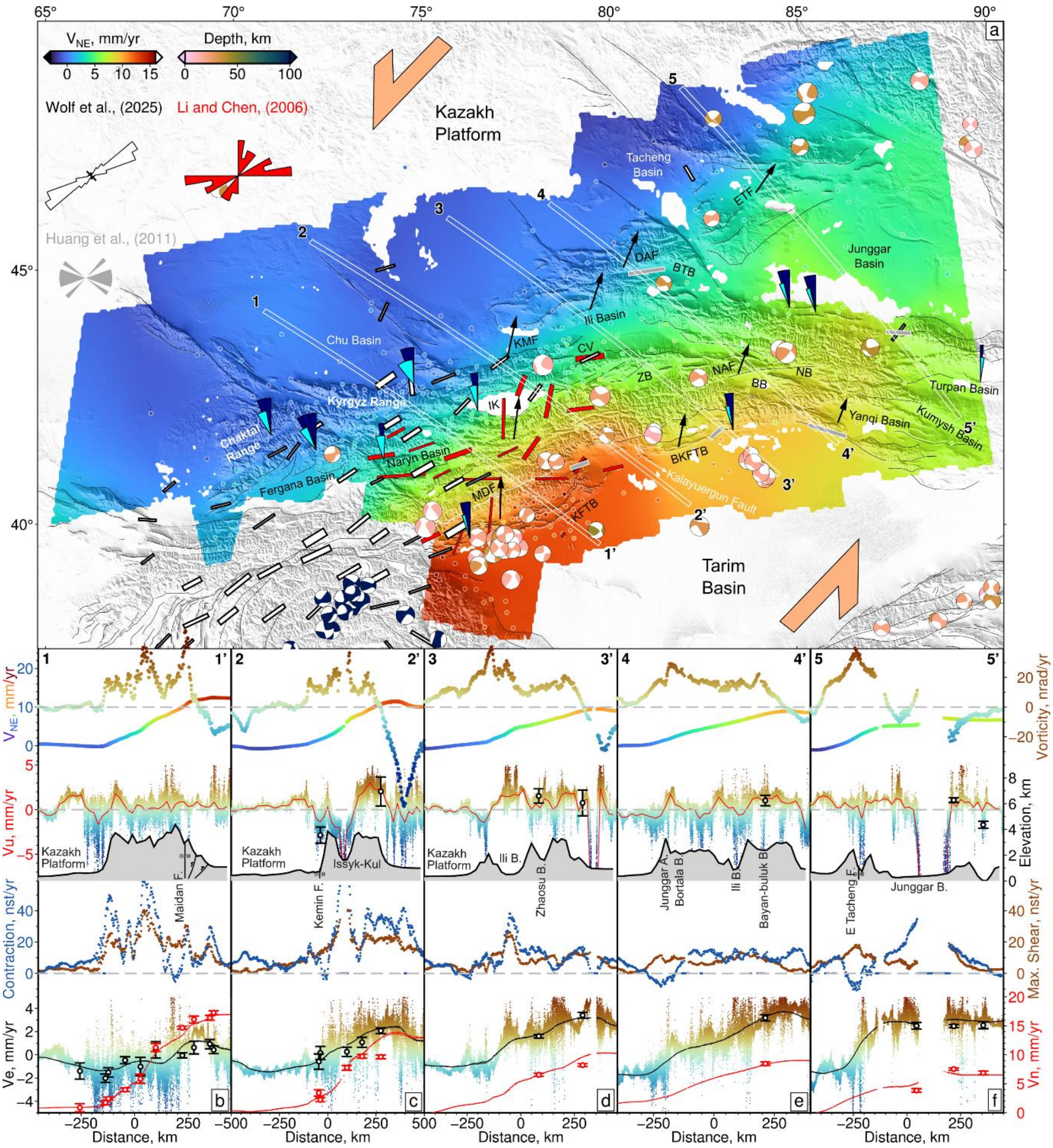

• **Nascent shear zone** You state that we observe here the "birth" of a shear zone. If true, do we find similarities (in topography) in well-developed other shear zone world wide or also in lab experiments? Do we see remnants of (rotated) ranges on both sides of a large strikeslip fault system, e.g. North Anatolian fault (that grew westward), East Anatolian fault, Altyn Tagh, or maybe Southern Alpine fault, San Andreas fault etc. . Also, is there evidence in the Tian Shan that the wedge-shaped faults maybe rotate over time to (in the geologic future) then more align with the strike of the broad shear zone?

Thank you for your question. We cite the Alps as a similar tectonic regime where the dextral strike-slip Peri-Adriatic Line developing through the Alps as the African plate pushes the Adriac plate to indent into the Alps through a clockwise rotation (Fig.4c, Lines 798-829). We also note the mismatch between the general trends of the Kyrgyz range and the fast axes of the shear wave splitting measurements underneath as evidence of rotation, not because of the rotation of the Tarim Basin,

but because of the clockwise rotation of the Fergana Basin, which probably co-created the left-stepping kink on the Talas-Fergana Fault that you drew our attention to (Lines 673-681). We also cite a newly published paper (Xie et al., 2026, https://doi.org/10.1016/j.epsl.2026.119997*) showing the Paleomagnetic vertical-axes rotation records along the Altyn Fault and through the Qilian Shan, which recorded a shift of tectonic regime at ~12.4 Ma (Lines 995-1002). We tried to look for similar evidence across the Tian Shan, but only found reported paleomagnetic records on the edges rather than interior of the Tian Shan. These data were also interpreted as showing local rotations only by their authors (Lines 704-740 and 979-995). Hence we encourage the acquisition of new paleomagnetic measurements in the interior Tian Shan in our revised manuscript, and used their conclusion of the local rotations to support our hierarchical wedge model which allows individual triangular wedges southeast half of the Tian Shan to rotate and translate in line with the stress field, rather than following a systematic sense of rotation like a rigid body rotation.*

• **Chaman-Karakul-Tien Shan shear zone** I my opinion it is a simplification to state that Chaman-Sarez-Karakul-Tian-Shan form one single shear zone, as they are clearly offset, kinematically and structurally, the reason of which could be discussed. My explanation would be the geometry of the Indian plate, but maybe there is another reason?

Thank you for your question. We agree that these structures are kinematically and structurally offset, and agree on the importance of the Indian plate. Following your suggestion, we read more about the literature on seismic tomography under Pamir, Tarim and the Tian Shan, and models of the interlocking between the Tian Shan and Indian lithosphere. In the process, we also found literature suggesting the contact between Indian and Tarim lithosphere with a proposed timing of 10Ma. This timing matches well evidence of accelerated uplift, exhumation and sedimentation across the Tian Shan. Hence, we now propose that this collision between Indian and Tarim lithosphere is the cause of the rapid uplift of the Tian Shan, and might have caused a southward shift of Tarim's Euler Pole that rotated the stress field (Fig.4b). This Euler Pole shift would explain the development of the nascent NE-trending shear zone, and the slowing down of the NW-striking dextral faults in the Kazakh Platform (Section 2.3, Lines 796-926).

Under this model, we synthesise all the evidence and propose that the shear strain, vorticity and slip lines are continuous on a grand scale because of the Chaman-Karakul-Tien Shan shear zone are all driven by the NNE-directed push from the Indian plate. They are offset because the shear zone through Tian Shan is driven through the Tarim Basin as a second indenter (Neil and Houseman, 1997) after being collided in the southwest by the Indian lithosphere (Fig.4b, Lines 599-624, Huangfu et al., 2021). The different degrees of maturity come from the temporal sequence of events through the different tectonic regimes. Below we show three figures from Wang et al., Kreemer et al., and Elliott et al., to show the continuity of the slip line, shear strain, vorticity features and their alignment from Chaman-Karakul to the Tien Shan shear zone.

[REDACTED]

[REDACTED]

Wang, L., Barbot, S.: Three-dimensional kinematics of the India–Eurasia collision. Communications Earth & Environment 2023 4:1 4, 164 (2023) https://doi.org/10.1038/s43247-023-00815-4

Kreemer, C., Blewitt, G., Klein, E.C.: A geodetic plate motion and Global Strain Rate Model. Geochemistry, Geophysics, Geosystems 15, 3849–3889 (2014) https://doi.org/10.1002/2014GC005407

Elliott, J.R., Fang, J., Lazeck´y, M., Maghsoudi, Y., Ou, Q., Payne, J.A., Rollins, C., Wang, D., Hooper, A., Wright, T.J.: Deformation, strains and velocities for the Alpine Himalayan Belt from trans-continental Sentinel-1 InSAR & GNSS. Remote Sensing of Environment 338, 115320 (2026) https://doi.org/10.1016/j.rse.2026.115320

Neil, E.A., Houseman, G.A.: Geodynamics of the Tarim Basin and the Tian Shan in central Asia. Tectonics 16, 571–584 (1997) https://doi.org/10.1029/97TC01413

Huangfu, P., Li, Z.H., Zhang, K.J., Fan, W., Zhao, J., Shi, Y.: India-Tarim Lithospheric Mantle Collision Beneath Western Tibet Controls the Cenozoic Building of Tian Shan. Geophysical Research Letters 48, 2021–094561 (2021) https://doi.org/10.1029/2021GL094561

• **GNSS/InSAR data independence** I very much enjoy large-scale InSAR rate maps and how they present surface deformation. But the text should clearly state that the longwavelength InSAR rates are mostly (purely?) constrained by the excellent GNSS velocity field of the region. This also touches the discussion whether the community should invest on cheap GNSS coverage or InSAR. It would be great to learn more about your experiences on that matter.

Thank you for your question. We have now stated clearly the importance of GNSS for mosaicking InSAR velocities, and cited examples from our velocity field to show problems arising from imperfect referencing. We suggest that the InSAR and GNSS experts collaborate strategically to ensure good distribution of GNSS stations in view of Sentinel-1 and NISAR track footprints.

Lines 220-323
"About 2 mm/yr of dextral motion can be measured on the Karatau-Talas-Fergana Fault system, but it is distributed across ~200km on each side (Fig. 1d, 2e), consistent with the multiple branches on

either side of the fault system (Fig. 2c). The long-wavelength eastward velocity between the Karatau-Talas-Fergana Fault and the Aktas Fault, that partially induces the east velocity gradients on the Karatau-Talas-Fergana Fault (Fig. 2c,e), is not the best constrained due to the lack of GNSS stations in this region"

Lines 1327-1345:
"However, high-quality GNSS velocities will still be essential for mosaicking InSAR velocities over multiple tracks. It is important that we have a good distribution of more than 4 3-dimensional GNSS data at locations we expect InSAR to be coherent to anchor the planar ramp of each InSAR frame. Imperfect referencing due to the lack of GNSS constraints, for example in non-deforming regions around the Karatau-Talas-Fergana Fault and within the Tarim Basin (Fig. 1b, Fig. S12), led to strain artefacts along InSAR track boundaries (Fig. 2c). Therefore, it would be desirable for the InSAR and GNSS communities to collaborate strategically, in view of the footprint of radar satellites, to improve the spatial distribution of GNSS data acquisitions. This step is crucial to referencing the next-generation continental-scale high-resolution velocity maps [87]."

• **More references** If the format allows I would include more references in the discussion. Your data also shows mass loss signal and permafrost melt but you do not go much into detail, for example, guiding the reader to further works that explain how these processes could be modeled.

Thank you for your suggestion. We have now added the following references to mass loss signals and permafrost freeze-and-thaw signals to guide the readers to further works modelling those signals (Section 2.5, Lines 1072-1246).

Watine, H., Daout, S., Lav´e, J., Doin, M.P.: Downslope solifluction movements and permafrost degradation in the northeastern Qinghai-Tibetan Plateau revealed by InSAR. Remote Sensing of Environment 329, 114926 (2025) https://doi.org/10.1016/J.RSE.2025.114926

Liu, S., J´onsson, S.: Glacial Isostatic Uplift in the Tibetan Plateau and Surroundings. Geophysical Research Letters 52, 2025–114899 (2025) https://doi.org/10.1029/2025GL114899

Huang, W.J., Fitrianto, M.T., Chen, C.C., Chang, P.Y., Yen, I.C., B´eon, M.L., Lu, S.T.: Complexity of near-surface deformation and subsurface structure of the Chihshang creeping fault-line scarp, eastern Taiwan: insights from integration of geological and geophysical data. Earth, Planets and Space 2025 77:1 77, 10 (2025) https://doi.org/10.1186/s40623-025-02135-7

Wu, P., Steffen, R., Steffen, H., Lund, B.: In: Steffen, H., Olesen, O., Sutinen, R. (eds.) Glacial Isostatic Adjustment Models for Earthquake Triggering, pp. 383–401. Cambridge University Press, Cambridge (2021)

Steffen, R., Steffen, H., Wu, P., Eaton, D.W.: Stress and fault parameters affecting

fault slip magnitude and activation time during a glacial cycle. Tectonics 33, 1461–1476 (2014) https://doi.org/10.1002/2013TC003450

Steffen, R., Wu, P., Steffen, H., Eaton, D.W.: The effect of earth rheology and ice-sheet size on fault slip and magnitude of postglacial earthquakes. Earth and Planetary Science Letters 388, 71–80 (2014) https://doi.org/10.1016/j.epsl.2013.11.058

Steffen, R., Wu, P., Steffen, H., Eaton, D.W.: On the implementation of faults in finite-element glacial isostatic adjustment models. Computers & Geosciences 62, 150–159 (2014) https://doi.org/10.1016/j.cageo.2013.06.012

Chang, S.H., Wang, W.H., Lee, J.C.: Modelling temporal variation of surface creep on the Chihshang fault in eastern Taiwan with velocity-strengthening friction. Geophysical Journal International 176, 601–613 (2009) https://doi.org/10.1111/j.1365-246X.2008.03995.x

Aagaard, B.T., Knepley, M.G., Williams, C.A.: A domain decomposition approach to implementing fault slip in finite-element models of quasi-static and dynamic crustal deformation. Journal of Geophysical Research: Solid Earth 118, 3059–3079 (2013) https://doi.org/10.1002/jgrb.50217

Aagaard, B., Knepley, M., Williams, C.: geodynamics/pylith v4.2.0. https://doi.org/10.5281/ZENODO.14635926

Carlson, G., Werth, S., Shirzaei, M.: A novel hybrid GNSS, GRACE, and InSAR joint inversion approach to constrain water loss during a record-setting drought in California. Remote Sensing of Environment 311, 114303 (2024) https://doi.org/10.1016/j.rse.2024.114303

Amos, C.B., Audet, P., Hammond, W.C., B¨urgmann, R., Johanson, I.A., Blewitt, G.: Uplift and seismicity driven by groundwater depletion in central California. Nature 2014 509:7501 509, 483–486 (2014) https://doi.org/10.1038/nature13275

Dietrich, R., Ivins, E.R., Casassa, G., Lange, H., Wendt, J., Fritsche, M.: Rapid crustal uplift in Patagonia due to enhanced ice loss. Earth and Planetary Science Letters 289, 22–29 (2010) https://doi.org/10.1016/j.epsl.2009.10.021

Teshebaeva, K., Echtler, H., Bookhagen, B., Strecker, M.: Deep-seated gravitational slope deformation (DSGSD) and slow-moving landslides in the southern

Tien Shan Mountains: new insights from InSAR, tectonic and geomorphic analysis. Earth Surface Processes and Landforms 44, 2333–2348 (2019) https://doi.org/10.1002/esp.4648

Brain, M.J.: Past, Present and Future Perspectives of Sediment Compaction as a Driver of Relative Sea Level and Coastal Change. Current Climate Change Reports 2, 75–85 (2016) https://doi.org/10.1007/s40641-016-0038-6

Payne, J.A., Watson, A.R., Maghsoudi, Y., Ebmeier, S.K., Rigby, R., Lazeck´y, M., Thomas, M., Elliott, J.R.: Widespread Extent of Irrecoverable Aquifer Depletion Revealed by Country-Wide Analysis of Land Surface Subsidence Hazard in Iran. Journal of Geophysical Research: Solid Earth 130, 2024–030367 (2025) https://doi.org/10.1029/2024JB030367

Pintori, F., Serpelloni, E., Gualandi, A.: Common-mode signals and vertical velocities in the greater Alpine area from GNSS data. Solid Earth 13, 1541–1567 (2022) https://doi.org/10.5194/se-13-1541-2022

Suggestions for Figures

Figure 1 Suggestions for better distinguishing features in this dense plot: In a) reduce the contrast in the background (using weak colors or transparency). You could also thin out the high number of GNSS and do not show the rate uncertainties to make them easier to identify and be able to enlarge their size somewhat. In b) you could remove the gray track lines and make the arrows a bit thinner. Instead of using shaded background it might be lighter (but as informative) if you use a minimized topographic color map (1000+m). Subfigures c-f could be moved to the supporting material.

Thank you for your detailed suggestions.

(a) *We have reduced the contrast in the background by applying 50% transparency. We now plot 1/3 of the GNSS stations and removed the rate uncertainties to improve clarity. We have doubled the size of the arrows to make them easier to identify.*
(b) *We have removed the gray track lines and made the arrows 20% thinner. We have replaced the shaded background with the same colour as in panel a, and added a colorbar for elevation.*

(c-f) We have moved these subfigures to the supporting material.

(c-new) We have moved the prior conceptual model of Wu et al., 2023 from the original Figure 4d to the the new Figure 1c and labelled the fault names for better introduction of the tectonic overview.

(d-e) We have moved the unfiltered Ve and Vu from the original Figure 2a,b to the new Figure 1d,e to reduce clutter in Figure 2, and added NW, SW, SE and NE labels for better geographical referencing.

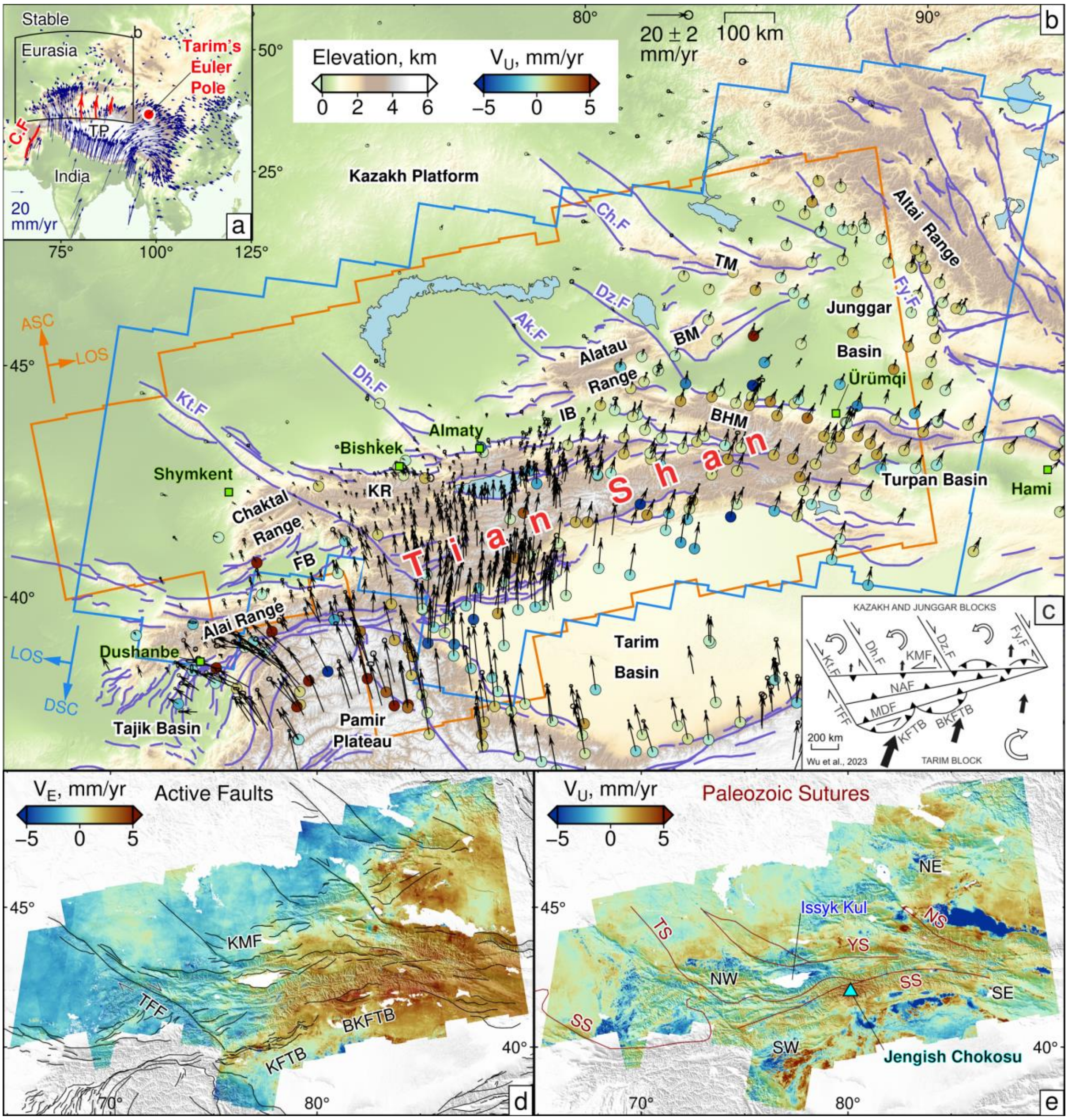

Figure 2 The line width of the colorbar is unnecessarily thick. Check whether all subfigures are needed or if their number could be reduced and instead increase the size of individual subfigures for readability.

Thank you for the suggestion. We have reduced the line width of the colourbars. We moved the unfiltered Ve and Vu to Figure 1b and 1c so that we have 10 panels in Figure 2 instead of the original 12 panels. We have added new analysis between the azimuth misfit between the GNSS vectors and unit vectors in the sense of Tarim's rotation calculated at the GNSS locations. We plotted the vectors and misfit wedges on top of the filtered Ve and Vn to illustrate the impact of Tarim's rotation in the triangular wedge showing eastward motion (Fig.2a,b). We have also added the shear-wave splitting results from Kufner et al., 2018 to Figure 2e, together with the fault segments with strike-slip components greater than 1 mm/yr as identified by GNSS profiles (Wu et al., 2023) and kinematic models (Li et al., 2021). In addition, we have added deep seismicity from the ISC-GEM catalogue to panel g to discuss the geometry of the Indian plate. In panel (i), we have added newly digitised Paleozoic suture zones to show the potential connection between the NW-trending fast axes with the suture structures.

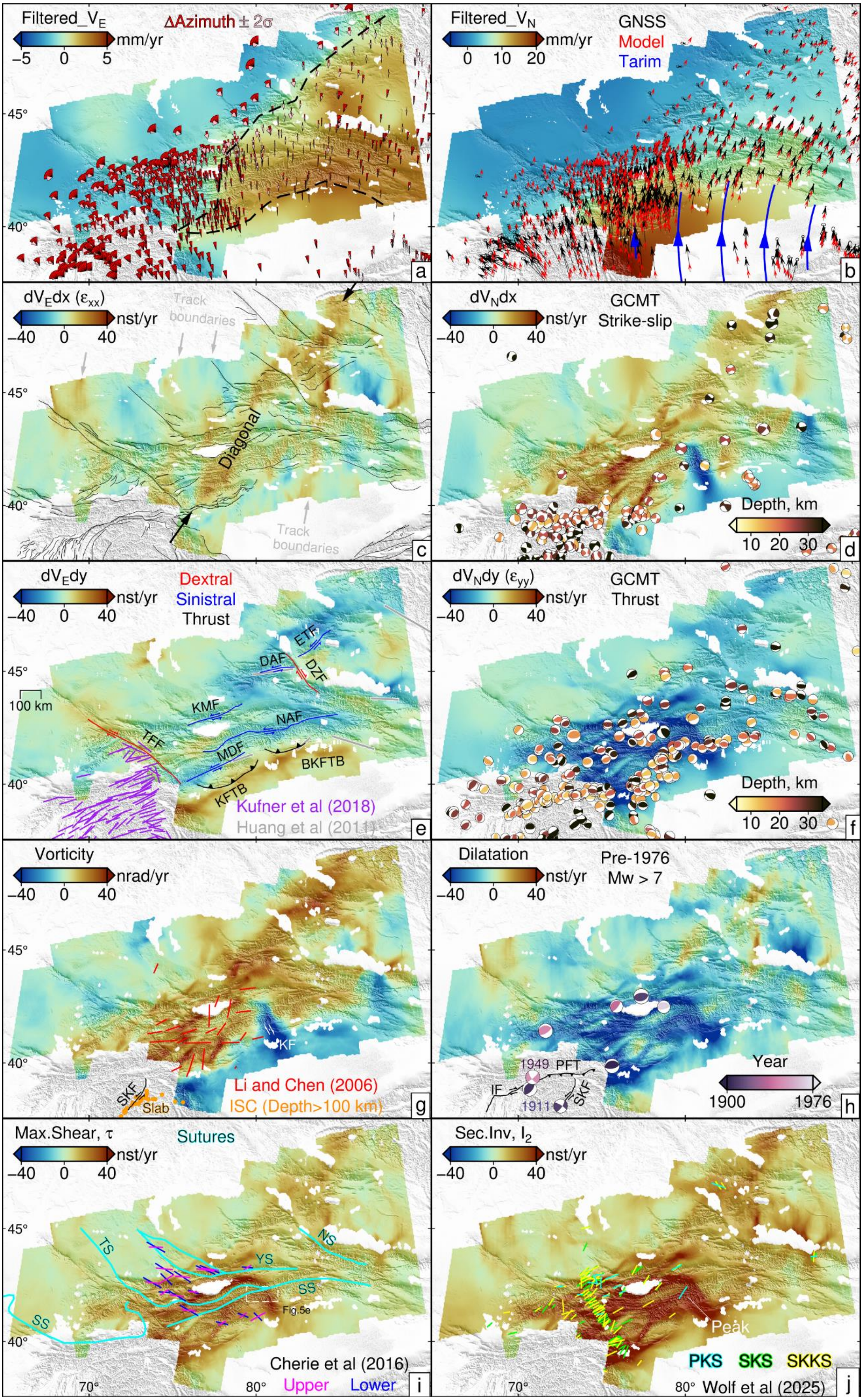

Filtered_V$_E$
ΔAzimuth ± 2σ
mm/yr
–5 0 5
45°
40°
a
Filtered_V$_N$
GNSS
Model
Tarim
mm/yr
0 10 20
b
dV$_E$dx (ε$_{xx}$)
Track boundaries
nst/yr
–40 0 40
Diagonal
c
dV$_N$dx
GCMT
Strike-slip
nst/yr
Depth, km
10 20 30
d
dV$_E$dy
Dextral
Sinistral
Thrust
100 km
ETF
DAF
DZF
KMF
NAF
TFF
MDF
BKFTB
KFTB
Kufner et al (2018)
Huang et al (2011)
e
dV$_N$dy (ε$_{yy}$)
GCMT
Thrust
f
Vorticity
nrad/yr
KF
SKF
Slab
Li and Chen (2006)
ISC (Depth>100 km)
g
Dilatation
Pre-1976
Mw > 7
1949
PFT
IF
1911
Year
1900 1976
h
Max.Shear, τ
Sutures
TS
YS
NS
SS
Fig.5e
Cherie et al (2016)
Upper
Lower
70°
80°
i
Sec.Inv, I$_2$
Peak
PKS
SKS
SKKS
Wolf et al (2025)
j

Figure 4 In the text the subfigures are not discussed in order. For clarification it would be helpful to use the same color coding in d) as in b) and c) , e. g., color the Tarim block lilac and use the same name (Tarim Basin, or actually block might be better?). Also it is unclear in b) what you mean by the Tian Shan "mirroring" the Western Tibetan Plateau (L355), maybe you could work with colors as well? I also find the Euler pole in b) confusing as it should point into the plane I would say and you could use curved red arrows in b) to d) as you do in a) to represent the block rotation of Tarim. Would it be possible to simplify b) somewhat?

Thank you for your detailed suggestions. We have adopted the same colour coding for all panels, with lilac for rigid blocks and yellow for weak mountain ranges. We have changed the representation of the Euler Poles in both panels a and b so that the arrow points into the surface. We have simplified panel b by removing the normal faulting symbols in the Tibetan Plateau and added the curved red lines to the arrows like those in panel a (and added grey curved lines to show the old stress field before the Euler Pole shift). In panel b, we have improved the drawing and figure caption to explain how the Tian Shan to the southeast of the cryptic shear zone has a tendency to develop into a similar tectonic regime as that of the Western Tibetan Plateau. The cryptic shear zone (red curve) could become the equivalent of the Altyn Tagh Fault separating the Tian Shan from the Kazakh Platform. The fold-and-thrust belts between the Tarim block and Western Tian Shan (black sawtooth) will rotate to strike EW and then NW, just like the Himalyan Frontal Thrust. In fact, the Fergana Basin pushing northwest into the Chaktal range whilst rotating anticlockwise and causing displacement on the arcuate Talas-Fergana Fault is also a mirror image of the Tarim and central-eastern Tian Shan on the west of the Talas Fergana Fault.

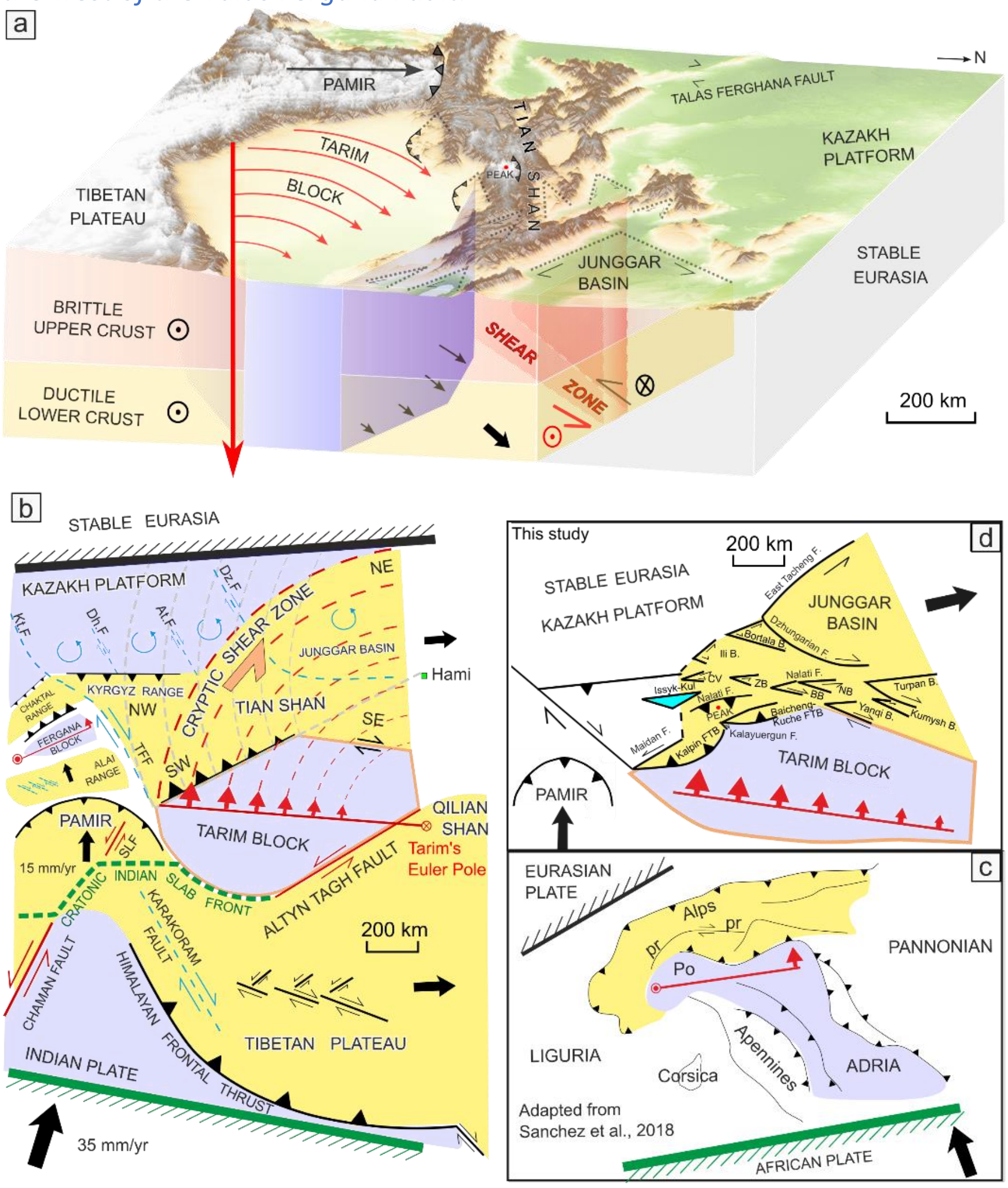

Figure 5 You mention some triangles but I cannot find them (subfigure e and f, I believe).

Thank you for spotting the error. We have now added the triangles to subfigure f and added dotted cyan line to represent the South Tian Shan Suture which coincides with the drainage divide on subfigure e.

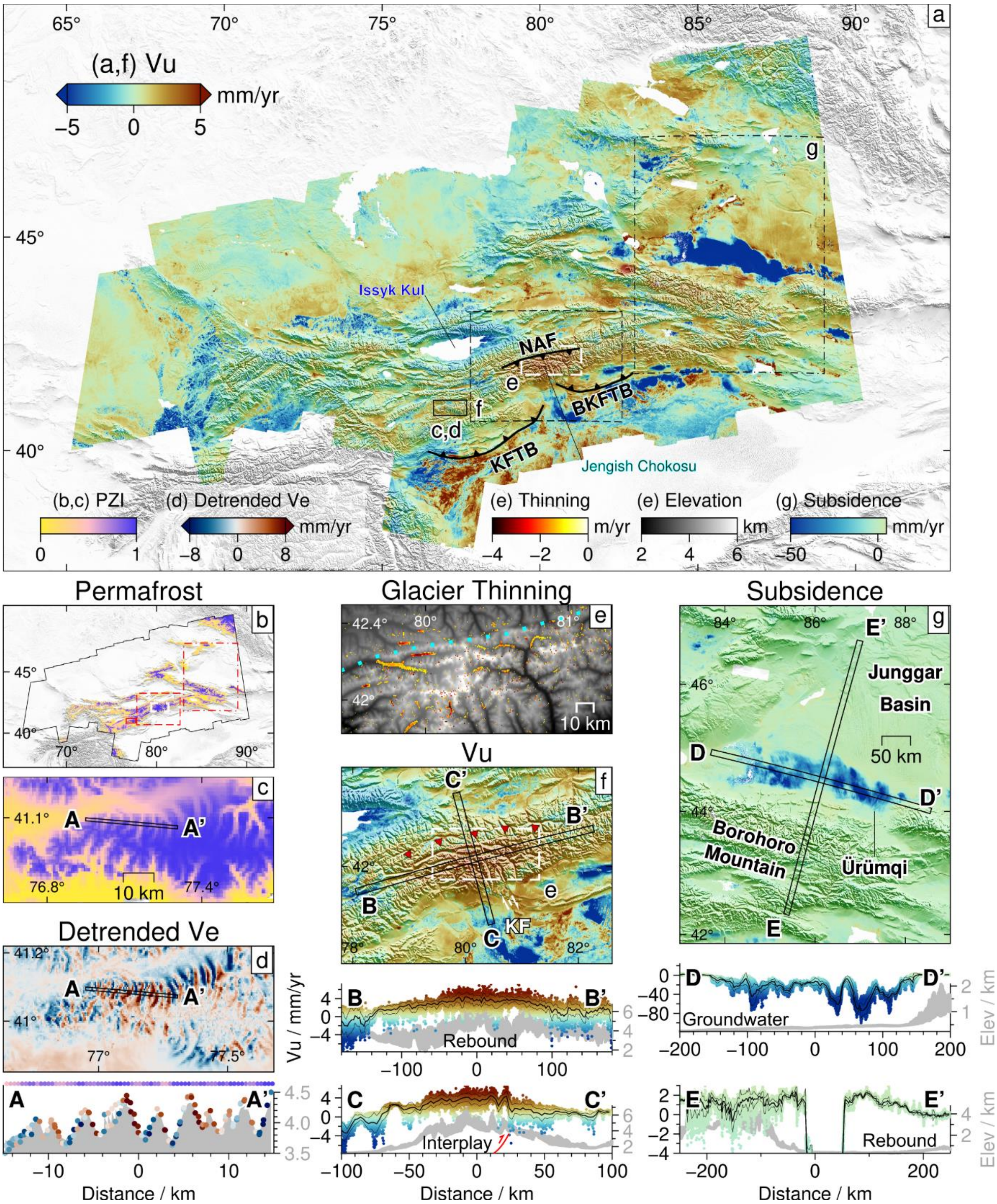

Detailed suggestions to improve clarity and completeness

Main Text

L63ff: When describing geographic locations I suggest to refer to Figure 1 and make sure that text and figure align. For example, the definition of "Kyrgyz Tian Shan" in line 66 is unclear as no political boundaries are plotted in Figure 1. You could mention maybe that South Tian Shan is more attached to Pamir, while the major part of Tian Shan is located between the basins or so, which is also why it accommodates more shortening? (I just see that you excluded the South Tian Shan from the analysis, maybe it is worth mentioning.) Later you use southwestern, western and eastern Tian Shan, I think you clearly should mark those regions somewhere.

Thank you for your suggestion. We have added "NW", "SW", "NE", "SE" labels to Figure 1e with reference to our InSAR data coverage, which indeed excluded the "South Tian Shan" north of the Pamir. We have replaced "Kyrgyz Tian Shan" with "western Tian Shan" to avoid confusion (Line 72). We have labelled more mountain and basin names to help refer to the specific parts of the Tian Shan (Fig 1). We have added a discussion of how the Pamir and the Tarim Basin might be pushing forward as a united front due to contact of the Indian mantle and Tarim block (Fig 4b) and propose that this contact might be a reason for the slowing down of the Talas Fergana Fault which separates the Tian Shan north of the Pamir and the Tarim, respectively:

Lines 69-72:
"Bounded between the rigid Tarim Basin in the south and the crystalline Kazakh Platform and Junggar Basin in the north (Fig. 1), the Tian Shan absorbs approximately half [4] (to nearly two-thirds in western Tian Shan [5]),"

Lines 877-885:
"As the Indian lithospheric mantle came into direct contact with the rigid deep lithospheric root of Tarim, like how the Indian and Asian lithospheric mantle are locking underneath Pamir today [54, 86], Pamir and the Western Tarim effectively became a united collisional front. They move north at similar rates, slowing the right-lateral strike-slip motion on the once-highly-active Talas-Fergana and Karakoram Faults."

L73: Can you provide a geologic time to the extension activity?
Thank you for your question. We have expanded on Tian Shan's complex tectonic history with more specific geological times.

Lines 79-96:
"However, Tian Shan's complex tectonic history has resulted in significant structural heterogeneity and kinematic variability across the mountain belt [7–9]. Four sub-parallel range-cutting NW-trending suture zones (Fig. 1e) were created by Late Proterozoic island arc accretion, Ordovician-to-Devonian back-arc rifting [10], and early Permian closure of oceanic basins [11, 12]. Early Mesozoic quiescence flattened topography, before late Mesozoic tectonics [13] and the major Cenozoic orogenic event, from the collision of India in the far south, rejuvenated the Paleozoic structures to accommodate over 200km of north-south shortening in western Tian Shan (~76.6◦E), and about 125km of shortening in eastern Tian Shan (~85.5◦E) [14, 15]."

L96: Can this paragraph be extended with more than just saying that you will present data, e. g., what products you will derive and how it helps to solve the problems raised in the paragraph above?

Thank you for your suggestion. We have expanded this paragraph to state how our velocity gradients and vorticity helped reveal a NE-trending shear zone that is playing a more important role in accommodating shear than the platform cutting strike-slip faults, and how our vertical velocities reveal spatially heterogeneity in uplift mechanisms.

Lines 149-187:

"The east velocity gradients and vorticity fields (Fig. 2c,e,g) reveal a newly identified range-scale and range-cutting arcuate left-lateral shear zone trending northeast. Based on our velocity, vorticity and strain rate mapping, and from earthquake focal mechanisms (Fig. 2d,f,h) and previously published shear wave splitting (Fig. 2e,g,i,j) and paleomagnetic rotation measurements (Fig. 3a), we explore the possibility that the Tian Shan is in the early stage of developing a range-cutting shear zone centered along a NE-trending diagonal (Fig. 2a,b, 3, 4). We show that this newly identified shear zone is driven by the clockwise rotation of the Tarim Basin, and propose that its nascent nature is due to a southward shift of the Tarim's Euler Pole following the Indian-Tarim lithospheric contact which changed the stress field and slowed down the platform-cutting strike-slip faults, such as the prominent Talas Fergana Fault. We further propose a hierarchical triangular wedge model to explain the sense of the strike-slip component motions on individual basin-bounding faults across the southeastern half of the Tian Shan. These conjugate strike-slip component motions across different spatial scales collectively play a more important role in accommodating the shortening and extension of the Tian Shan in this sinistral range-scale shear zone than the dextral slip on the platformcutting strike-slip faults. By examining our vertical component of the derived surface velocity (Fig. 5), we show how the rates, spatial scales, and mechanisms of uplift are heterogeneous across the mountain belt, with glacial isostatic adjustment potentially directing the localisation of shortening and uplift near the highest peak in southern Tian Shan (Jengish Chokusu, Tomur Feng, or Victory Peak, 7,439 m, Fig. 1e)."

L113: Instead of just mentioning the rate difference you could directly describe to which direction the east rates increase, which would use same space, but is more informative.

We have modified the sentence to:

Lines 195-200:

" Our derived east velocities show a significant extension of Tian Shan along its strike with an east component rate increasing from south-west-west to north-east-east by ~5 mm/yr (one quarter of the total convergence rate), over a distance of ~2000km (Fig. 1d)."

L73/111/246/ff: You mention extension in the Tian Shan several times but it is not always clear along which direction you mean. Since the key message is about this extension accommodated on a yet unidentified NE-shear zone, it would help to clarify throughout the text, of which type of extension you speak. Probably the extension mentioned in L73 is a different one?

Thank you for your question. Indeed, the extension mentioned in L73 is a different process. We have replaced the "extension" here with the more specific term "back-arc rifting" together with more detailed geological events.

Lines 79-96:

"Four sub-parallel range-cutting NW-trending suture zones (Fig. 1e) were created by Late Proterozoic island arc accretion, Ordovician-to-Devonian back-arc rifting [10], and early Permian closure of oceanic basins [11, 12]."

For L111 and L246, both refer to the current sense of extension along the strike of the Tian Shan range.

In L111 (now Line 196), we clarified the direction with the addition of "from south-west-west to north-east-east":
"Our derived east velocities show a significant extension of Tian Shan along its strike with an east component rate increasing from south-west-west to north-east-east by ~5 mm/yr (one quarter of the total convergence rate), over a distance of ~2000km (Fig. 1d)."

In L246 (now L363-367): we elaborated on the sense of extension with "suggesting a predominantly extensional regime in the northeast Tian Shan, with crustal materials moving northeastward, making space for the elongation of the Tian Shan range."

L173: one ']' to much
Thank you for identifying the mistake. We have removed the extra ']'.

L175-177: Are these average numbers per frame? Per Pixel? I am confused because you mention earlier that you processed over 100,000 ifgs in total. Should it be (more precise): "before/after quality control" rather than time-series?
Thank you for pointing out the confusion. We have moved the statistics panels from the main text to the supplementary information with the following clarified caption:

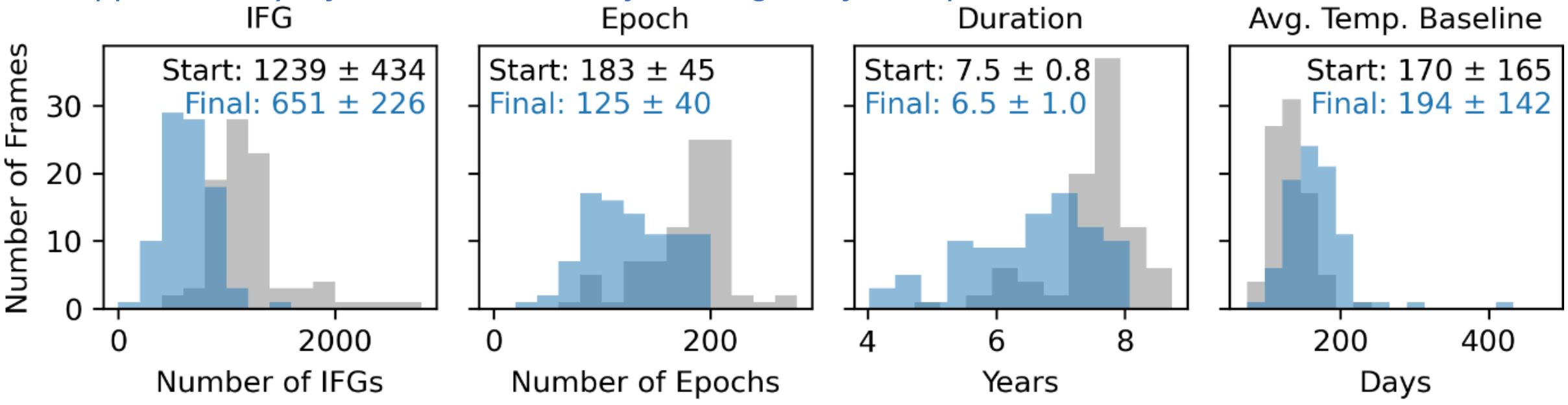

Figure S1: The per-frame statistics of number of interferograms, epochs, time span and average temporal baselines of interferograms before and after the quality control, through the improved LiCSBAS11 (Text S2) and newly developed LiCSBAS132 (Text S4) steps during the time series analysis.

L261: Figure 4d is referenced before the other subfigures of Figure 4.
Apologies for the mis-order of panels. We have moved the original Figure 4d to the new Figure 1c so that they are referenced in sequence.

L315: This could be material to mention at the end of the introduction?
Thank you for your suggestion. We have moved this sentence to the last paragraph of the introduction section.
Line 152-161:
"Based on our velocity, vorticity and strain rate mapping, and from earthquake focal mechanisms (Fig. 2d,f,h) and previously published shear wave splitting (Fig. 2e,g,i,j) and paleomagnetic rotation measurements (Fig. 3a), we explore the possibility that the Tian Shan is in the early stage of developing a range-cutting shear zone centered along a NE-trending diagonal (Fig. 2a,b, 3, 4)."

L399/568: Is "indented" a widely used term in the community for the kinematics or the shape of the Tarim basin? For me, an indenter is something more pointy.
Thank you for your question. We have checked the literature and find that the term "indent" appeared in literature that describes the Tarim as a secondary indenter that transmits the stress from the India-Eurasia collision to the Tian Shan (e.g., Neil and Houseman 1997 https://agupubs.onlinelibrary.wiley.com/doi/epdf/10.1029/97TC01413).

In recent literature, "rotation" is used the most often, with debate about the extent of thrusting or subduction of the Tarim material underneath the Tian Shan (Li et al., 2022 https://doi.org/10.1038/s41467-022-30795-6, Sun et al., 2022 https://doi.org/10.1130/G50510.1*).*

We have modified the kinematic description to be more in-line with recent literature:

Therefore, we have modified L399 to (new L514) "clockwise rotation of the rigid Tarim Basin", and modified L568 to (new L791) "pushes into the weaker Tian Shan"

L400-409 and 418 and 427 and 430: The block (or arc) rotation of the Tarim basin should be better visualized in the figures, e.g. Figure 4b-d and maybe also in Figure 1, see the white arrows in this unpublished figure (cf. Enrico Serpelloni) of the Adria plate as inspiration:
In addition, it is sometimes hard to follow what the azimuth numbers indicate (an angle between what and what?). Maybe you can add "XX°N" if they measure an azimuth clockwise from North? Angles on a map would help.
Thanks for your suggestion. We have added arrows like the unpublished figure you suggested in our Figure 1a. We have also redesigned Figure 2 to show unit vectors in the sense of Tarim's rotation to compare with the GNSS vectors in the new Figure 2a,b. We also add small arrows in the sense of Tarim's rotation in Figure 3a to better visualise the angle between the stress field and the fault strikes, and added these arrows to Figure 3b to contrast the old and new stress field due to the Indian-Tarim collision.

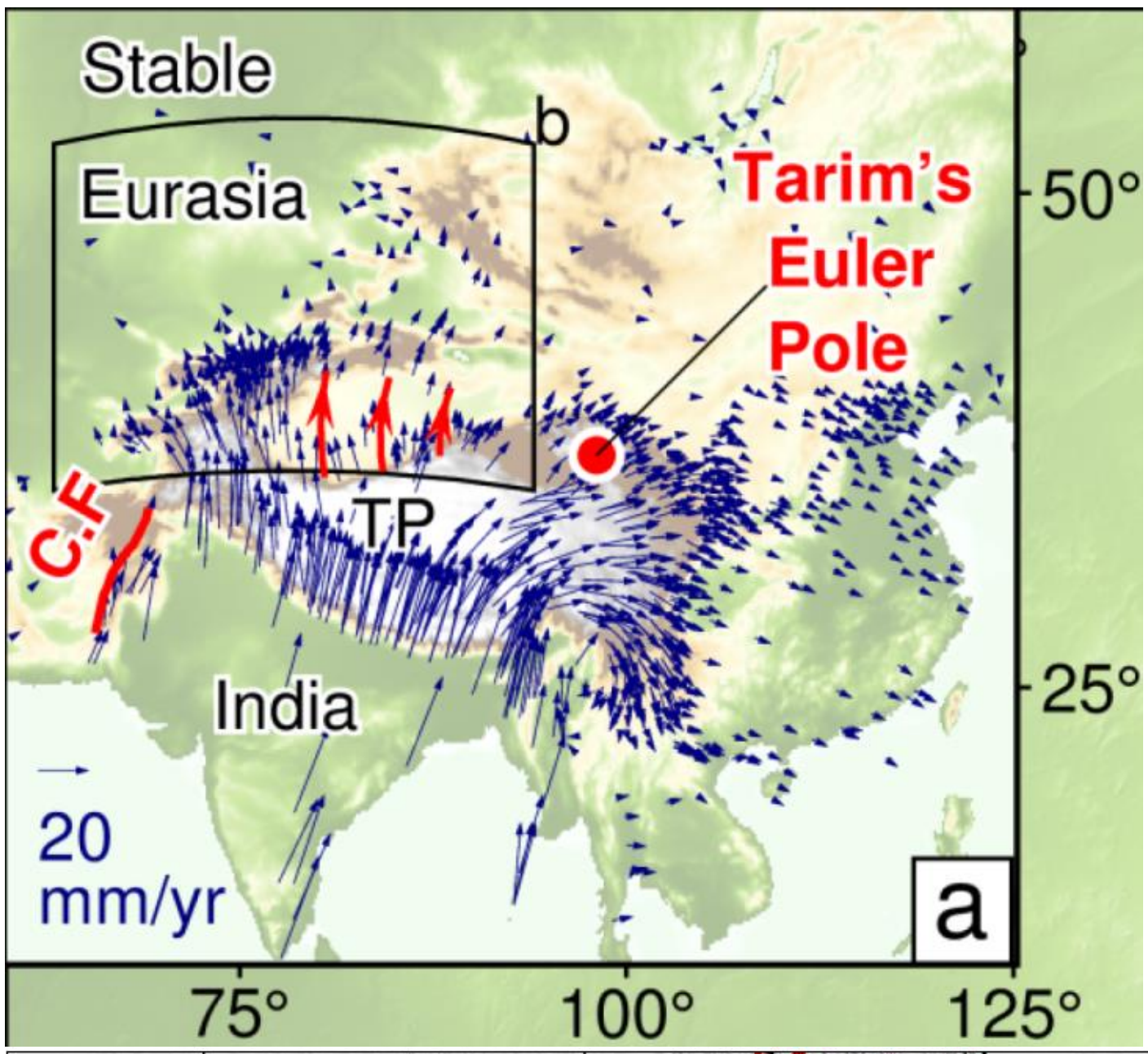

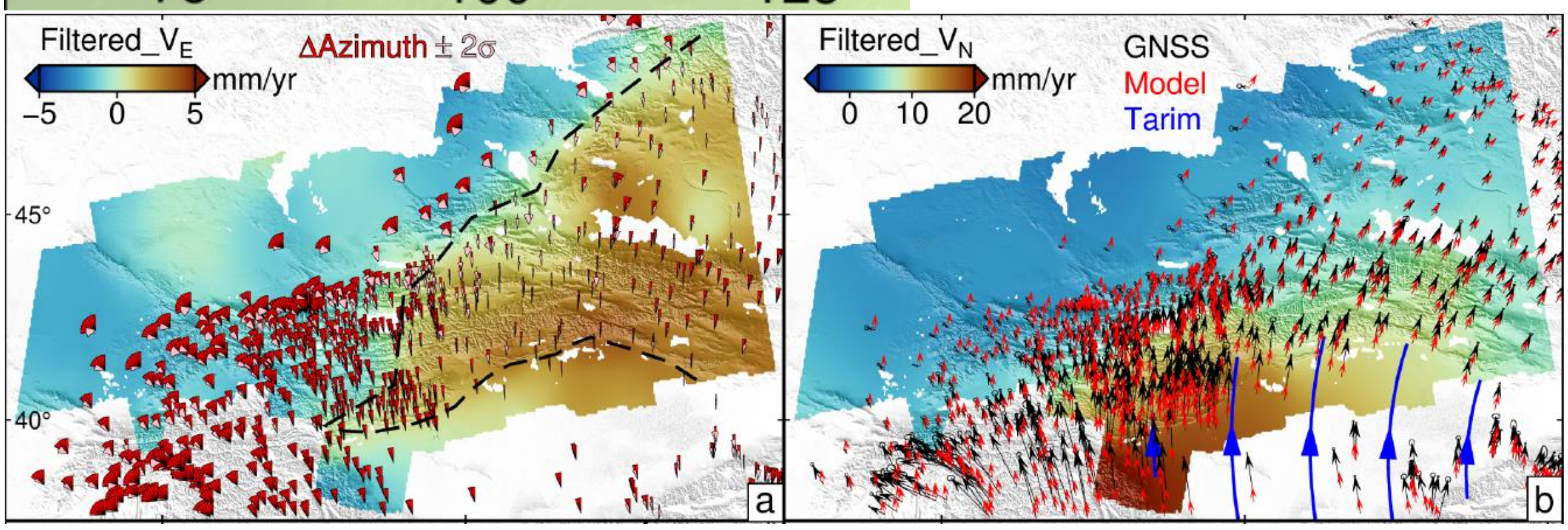

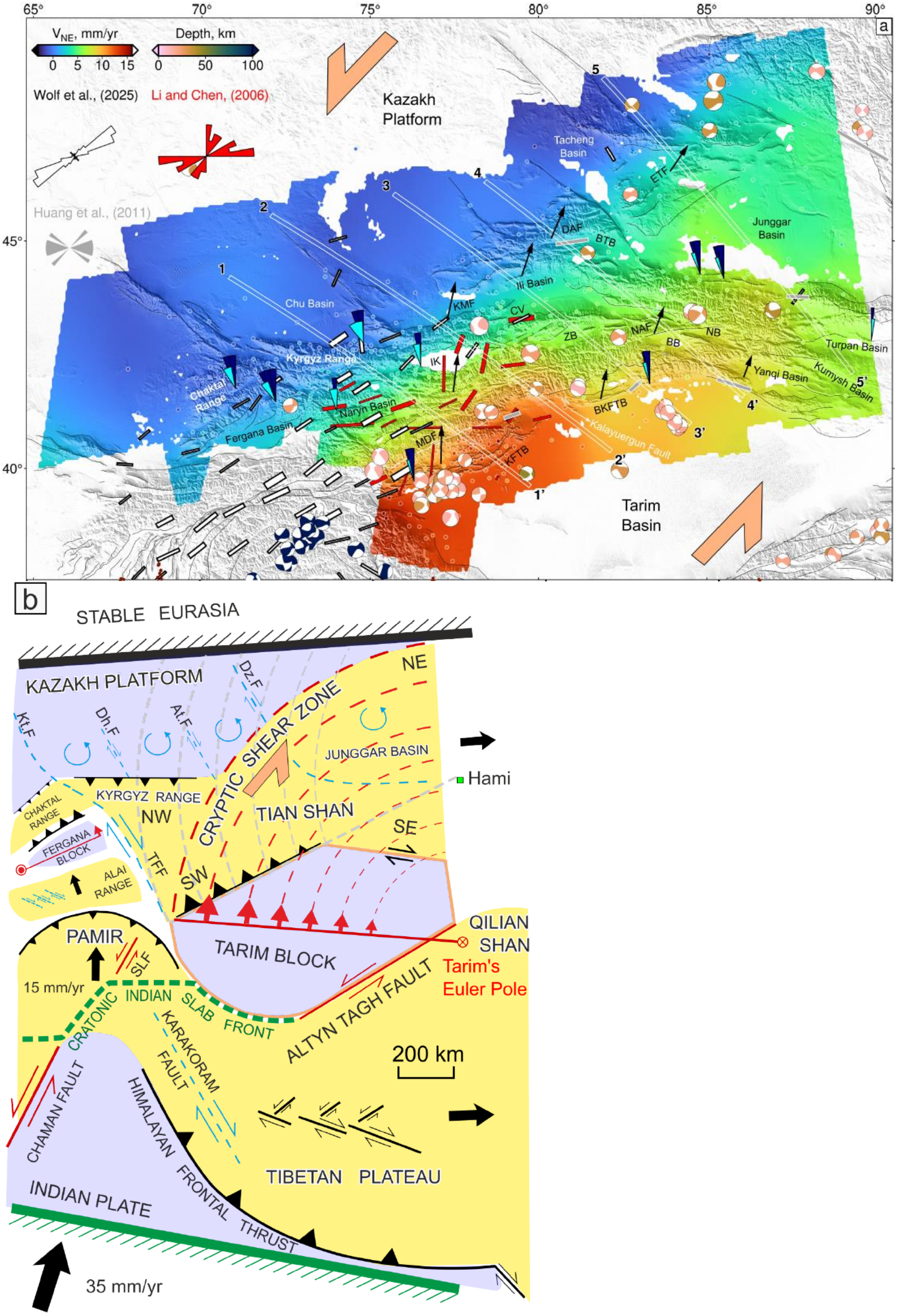

a
65°
70°
75°
80°
85°
90°
45°
40°
V_{NE}, mm/yr
0 5 10 15
Depth, km
0 50 100
Wolf et al., (2025)
Li and Chen, (2006)
Huang et al., (2011)
Kazakh Platform
Tacheng Basin
Junggar Basin
ETF
DAF
BTB
Ili Basin
Chu Basin
KMF
CV
ZB
NAF
BB
NB
Turpan Basin
Kumysh Basin
Yanqi Basin
Kyrgyz Range
IK
Chaktal Range
Fergana Basin
Naryn Basin
MDF
KFTB
BKFTB
Kalayuergun Fault
Tarim Basin
1
2
3
4
5
1'
2'
3'
4'
5'
b
STABLE EURASIA
KAZAKH PLATFORM
Kt.F
Dh.F
At.F
Dz.F
NE
CRYPTIC SHEAR ZONE
JUNGGAR BASIN
Hami
KYRGYZ RANGE
CHAKTAL RANGE
FERGANA BLOCK
ALAI RANGE
NW
TFF
SW
TIAN SHAN
SE
TARIM BLOCK
QILIAN SHAN
Tarim's Euler Pole
PAMIR
SLF
15 mm/yr
CRATONIC INDIAN SLAB FRONT
KARAKORAM FAULT
ALTYN TAGH FAULT
200 km
CHAMAN FAULT
HIMALAYAN FRONTAL THRUST
TIBETAN PLATEAU
INDIAN PLATE
35 mm/yr

L420-427: These two statements are somewhat contradicting: If the faults are nearly perpendicular the expected kinematics would be pure thrusting but then you say that the rates are partitioned?

Thanks for correcting. We rephrased the sentence to:
Line 549-555:
"The direction of Tarim's push (black arrows in Fig. 3a) is nearly perpendicular to the western tip of the Kalpin Fold-and-Thrust-Belt and oblique to the strike of the Maidan Fault, promoting pure thrust along the southern range-front and sinistral strike-slip fault to dislodge the southwest tip of the triangular wedge from the rest of the Tian Shan for its eastward escape."

L443-455: Could be partially explained in the method section if you need more space.)

Thank you for pointing out the lack of methodological explanation. There was a typo indeed – I meant to say that I filtered the focal mechanisms by the first rake of the two nodal planes. However, I now realise that this is not the most accurate. I have instead changed to filtering by the tension and null axes. On the GCMT search page: https://www.globalcmt.org/CMTsearch.html, there is the following instruction:
Use tension and null axis plunge to search by mechanism. For example, thrust faults have large plunge (>45) of tension axis, strike-slip faults have large plunge of null axis, and normal faults have small (<45) for both tension and null axes.

Hence, we have modified the description to:

Line 586-590:

"For post-1976 earthquakes, we divide the Global Centroid Moment Tensor (GCMT) Catalogue data [41] into strike-slip (plunge of null axis >45◦), thrust (plunge of tension axis >45◦), and normal (both plunges of tension and null axes >45◦) events."

L455-460: But they also nicely align with the individual mountain ranges of the Tien Shan, not only the band at depth.

Thank you for the question. We have reduced the clutter by performing block mean on the SKS results from Wolf et al., 2025 (Fig 3a) which only cover the part of the Tian Shan to the northwest of the shear zone. The SKS fast axes here do not align with the local mountain ranges. They are aligned with fast axes through the Pamir, which we interpret as being created by the same stress field due to Pamir's indentation.

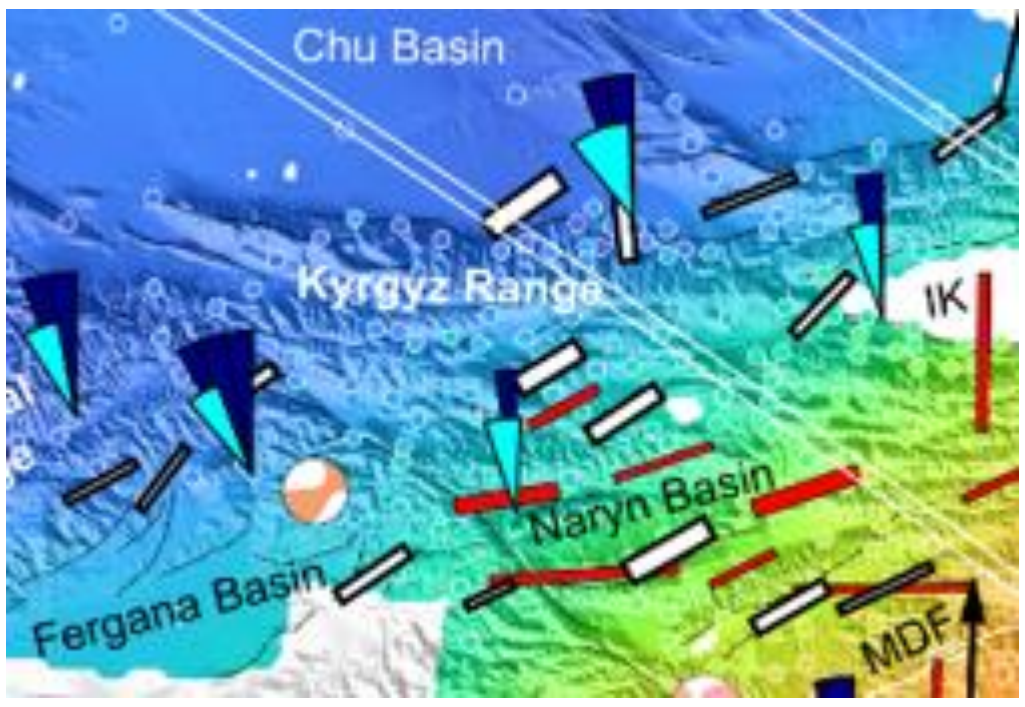

Line 673-681:
"Fast axes generally trend 60–70◦N, oblique to the local range strike (90–100◦N) of the Kyrgyz Range in northwest Tian Shan (Fig. 1b). We interpret this mismatch as arising from either the orientation of the Terskey Suture from the Paleozoic (Fig. 2i) or from the anticlockwise rotational indentation of the Fergana Basin that co-produced the left-stepping kink on the Talas-Fergana Fault (Fig. 4a). However,

these 60–70◦N fast axes are parallel to those along the Pamir front, consistent with a compressional stress field in the mantle induced by Pamir's push."

L468-473: But there is also a significant offset between these three structures, maybe related to the Indian indtenter? See Kufner et al. 2018a that propose a correlation of the Chaman-Badakhshan axis linked to marginal India, where as the Sarez-Karakul is linked to the cratonic India (https://doi.org/10.1029/2017TC004812, Figure 9) and Bloch et al. 2021 that completed the picture of the Eastern Pamir/Tarim (but is harder to interpret in how it might impact the NE-shear zone you detect, https://doi.org/10.1029/2021GL095413, Figure 4).

Thank you for the suggested reading about the structure under Pamir and how they relate to the crustal faults. Although the Indian indenter is now head-on against the Asian lithosphere underneath the Pamir, the continued push of the Indian plate is driving the underthrusted cratonic India towards the NNE. It is believed that the Indian slab is also against the southwest corner of the Tarim Basin, giving Tarim a torque for its clockwise rotation, which in turn bashes into the Tian Shan to push open an oblique shear zone. We expand the discussion in the newly added Section 2.3, and cite the key references about the contact between the Indian and Tarim lithosphere.

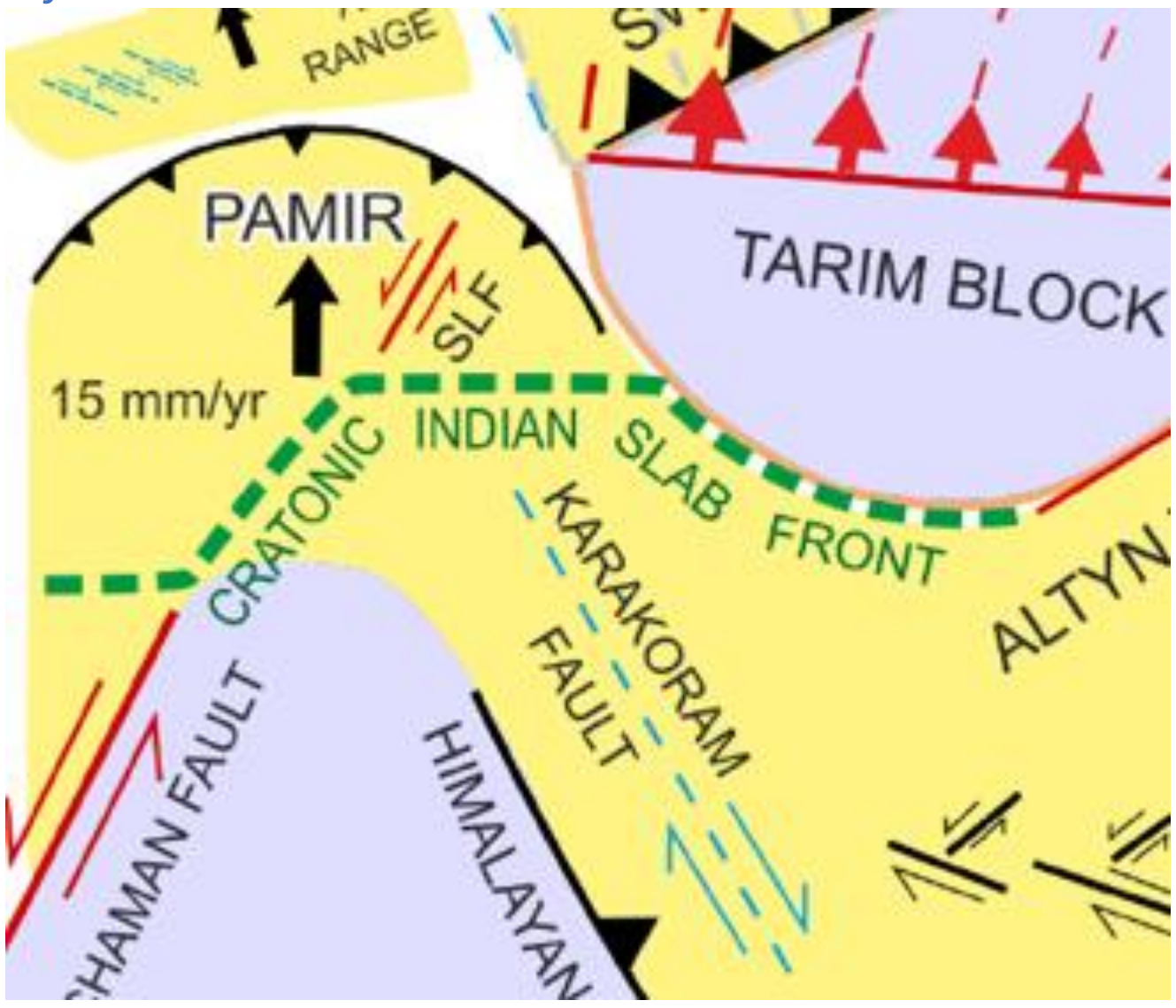

Zhao, J., Yuan, X., Liu, H., Kumar, P., Pei, S., Kind, R., Zhang, Z., Teng, J., Ding, L., Gao, X., Xu, Q., Wang, W.: The boundary between the Indian and Asian tectonic plates below Tibet. Proceedings of the National Academy of Sciences of the United States of America 107, 11229–11233 (2010) https://doi.org/10.1073/PNAS.1001921107

Huangfu, P., Li, Z.H., Zhang, K.J., Fan, W., Zhao, J., Shi, Y.: India-Tarim Lithospheric Mantle Collision Beneath Western Tibet Controls the Cenozoic Building of Tian Shan. Geophysical Research Letters 48, 2021–094561 (2021) https://doi.org/10.1029/2021GL094561

L481ff: Kufner et al. 2018B (https://doi.org/10.1029/2018JB015926) also present shear wave splitting data that does not cover new regions, but also shows some flow direction parallel to the TFF like Cherie et al. 2016.

Thanks for the reference. We have added the shear wave splitting results from Kufner et al., 2018 to Figure 2e. The fast axes from Kufner et al., 2018 are in the same direction as those from Cherie et al., 2016 but are not collocated. Those from Kufner et al are directly below the Talas Fergana Fault, but Cherie's are further east. We added Paleozoic suture zones to Fig 2i to suggest those NW trending lower-layer axes might be frozen flow lines from Paleozoic ocean closure events rather than from current flow.

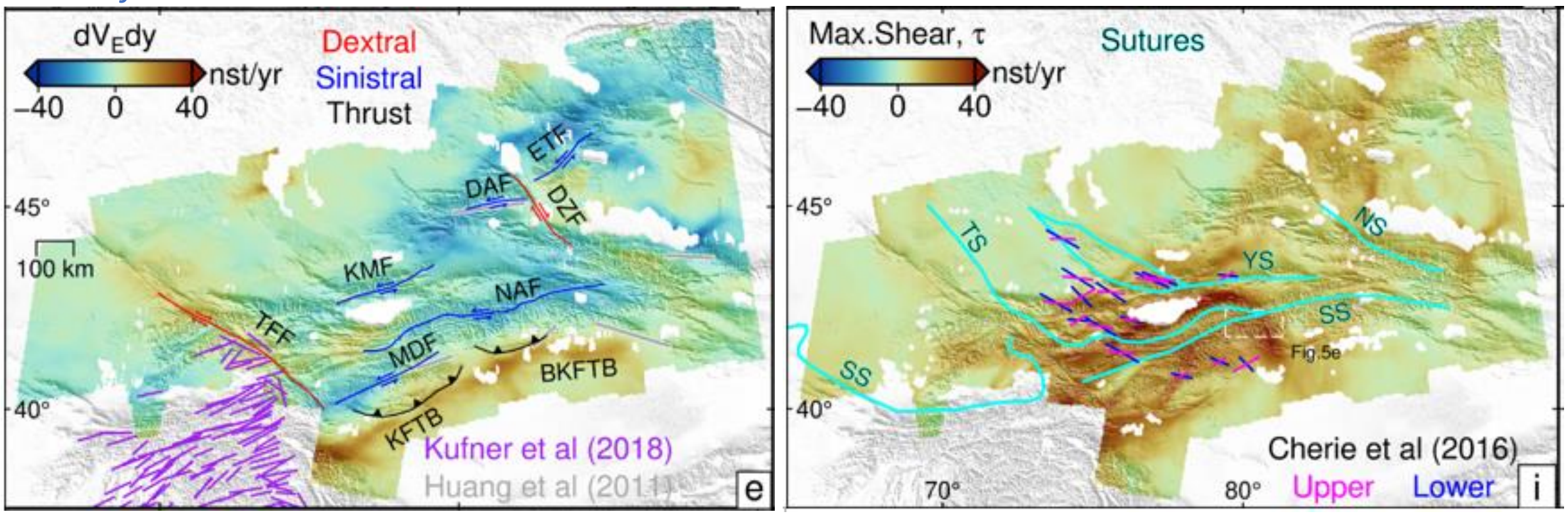

We discuss these features in Lines 636-658:
"However, our vorticity and strain rate patterns are at odds with the NW- and NNW-oriented fast axes derived from a two-layer model employed by Cherie et al. [57]. In particular, the NNW-directed lower layer anisotropy, interpreted as the NNW-directed mantle flow driven by the Tarim subduction in Cherie et al. [57], will not be compatible with our velocity field, unless the ductile portion of the lithosphere and the mantle are flowing almost perpendicular to each other; more likely, the orientation of the lower-layer anisotropy represents frozen fabric between the NW-trending suture zones generated during the Paleozoic closure of oceanic basins (Fig. 2i), rather than current flow direction. Anisotropy from Kufner et al. [54] highlights the NW-trending Talas-Fergana Fault (Fig. 2e), which is posited as a smaller-scaled, more-matured mirror reflection of the nascent NEtrending shear zone. It has a left-stepping kink against the Fergana Basin, likely due to the anticlockwise rotation of the Fergana Block driven by Pamir that co-produced the 100 km Tertiary displacement along the Talas-Fergana Fault [60](Fig 1a, Fig. 4b)."

L524-547: After the statements in this paragraph it would be interesting to discuss whether other large strike-slip fault systems went through similar stages before, for example, whether this is (still) reflected in the topography (Northern/Eastern Anatolian fault, northern part of the New Zealand Alpine fault, San Andreas fault?), and whether this is also reflected in the eastern Himalayan syntaxis? There is also numerical modeling done of how strike-slip faults are born (early analog: https://doi.org/10.1029/JB093iB12p15085
Thanks for prompting us to look for similar tectonic regimes worldwide and to compare our model of shear zone development in the Tian Shan with those described by early analogue and recent numerical models. The key difference between what we think is happening in the Tian Shan and the development stages of other large strike-slip fault systems lies in the presence of the Tarim Basin being a secondary rigid indenter being driven by the primary indenter (Neil and Houseman 1997 https://agupubs.onlinelibrary.wiley.com/doi/epdf/10.1029/97TC01413).

Following your suggestion, we found the Alps as going through a similar tectonic process with a strike-slip fault developing diagonal through a mountain range because of the rotation of a secondary indenter being driven by a primary indenter. Evidence suggests that the Euler Pole of the Adriac block also shifted in its recent tectonic past. We present the cartoon that we adapted from S´anchez et al, their present-day shear strain rate field (blue for sinistral, and red for dextral) and a tectonic plate reconstruction through the past 20 Ma for comparison.

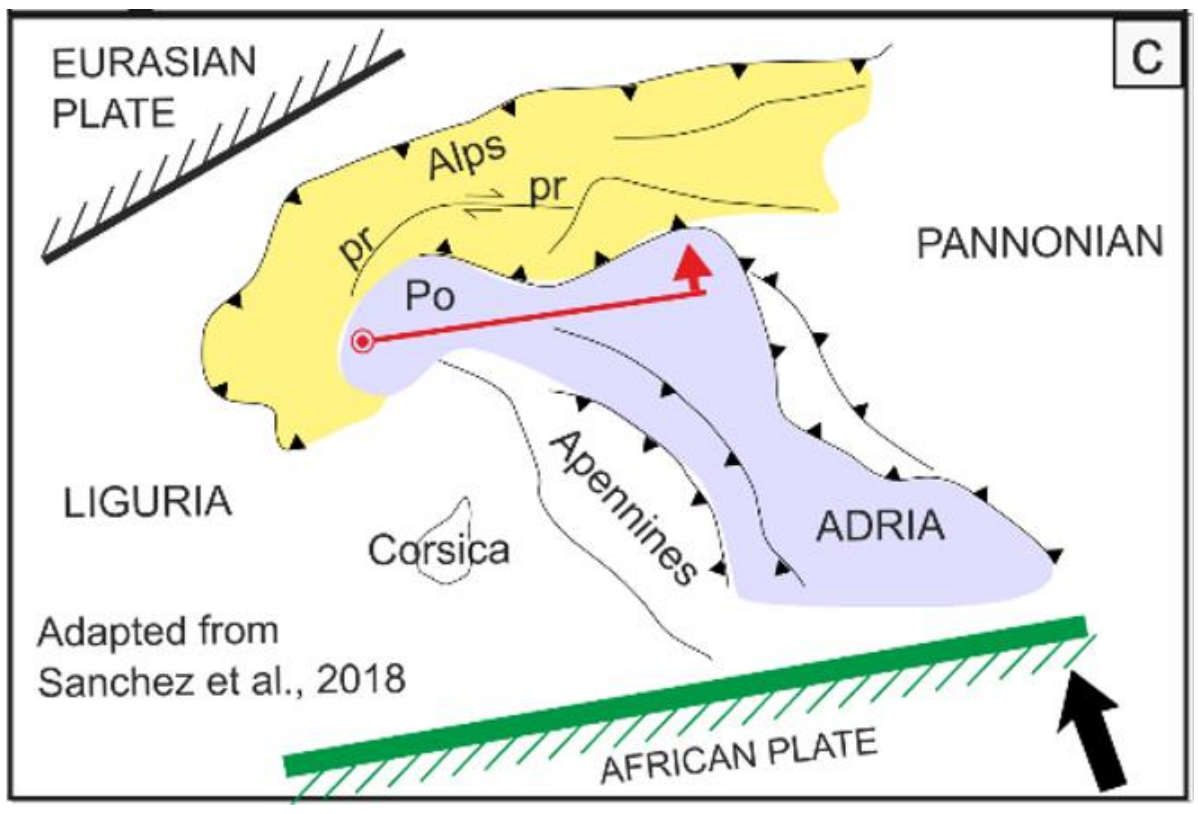
EURASIAN
PLATE
C
Alps
pr
pr
PANNONIAN
Po
LIGURIA
Apennines
ADRIA
Corsica
Adapted from
Sanchez et al., 2018
AFRICAN PLATE

[REDACTED]

Breton, E.L., Handy, M.R., Molli, G., Ustaszewski, K.: Post-20 Ma Motion of the Adriatic Plate: New Constraints From Surrounding Orogens and Implications for Crust-Mantle Decoupling. Tectonics 36, 3135–3154 (2017) https://doi.org/10.1002/2016TC004443
S´anchez, L., V¨olksen, C., Sokolov, A., Arenz, H., Seitz, F.: Present-day surface deformation of the Alpine region inferred from geodetic techniques. Earth System Science Data 10, 1503–1526 (2018) https://doi.org/10.5194/ESSD-10-1503-2018

We discuss the similarity between the Tian Shan and the Alps in Lines 798-827:
"We further explore the possibility of the southward shift of Tarim's Euler Pole being the cause of the "nascent" nature of the range-cutting sinistral shear zone through the Tian Shan, and compare this tectonic transition in Tian Shan with that in a similar tectonic regime in the European Alps (Fig. 4c). In the Alps, the NNW-headed farfield indenter of the African Plate is driving the anticlockwise rotation of the Adriaic microplate to create a right-lateral shear zone that is the Periadriatic Line through the Alps [97], mirroring how the NNE-headed India is driving the clockwise rotation of Tarim to create a left-lateral shear zone in Tian Shan. Both Adria and Tarim are promontories of much larger plates driving their motion. Due to irregular geometry and the random position of the secondary indenter relative to the first indenter, the torque generated by their collision could change the Euler Pole of the secondary indenter, hence changing the tectonic regime at its plate boundaries. Adria's Euler Pole was in Spain in the far west, when it first translated northwestward to indent Europe, causing the westward extrusion of the western Alps and the formation of the dextral Periadriatic Line[97]. After wedging the Adriatic toe in the western Alps, the Euler Pole changed to the Po valley to allow further northwest motion by indenting the Adriatic heel into the eastern Alps, driving present-day right-lateral shear around the heel [98], in the same way that Tarim is driving the cryptic shear zone in the Tian Shan."

L599: "many rigid blocks in Tian Shan" is this just a descriptive term or a proven statement (like micro/blocks)?
Thanks for the question. We initially used the term in a descriptive sense, but now realise the confusion this might cause. We recognise that not all regions between the conjugate fault pairs are proven to be rigid. Hence we have replaced the term with a more generalised term "crustal wedges" (L961).

L600: To facilitate an easy comparison of your conceptual model in 4c to the geographic features on a DEM it would be helpful if you repeat all features in Figure 4c also in Figure 1a and 3a. In your conceptual model the role of TFF and other faults with similar strike along the northwestern margin of the Tian Shan (DhF, AkF, DzF, ChF) remains unclear. The TFF is an impressive structure, but is completely misoriented to your stress field. It also is right-stepping which does not agree to the large sense of motion indicated in Figre 3a. Is it inherited from a previous stress field and how could that have looked like?
Thanks for the suggestion. We have repeated all the labels in the now Figure 4d in Figure 3a to facilitate the comparison between our conceptual model and the geographical features on the DEM. We repeated as many of the labels on Figure 1b as possible, but we had to omit the labels of Zhaosu, Nalati, Bayanbuluk, Yanqi and Kumysh Basins in Figure 1b as it's already too crowded in those regions.

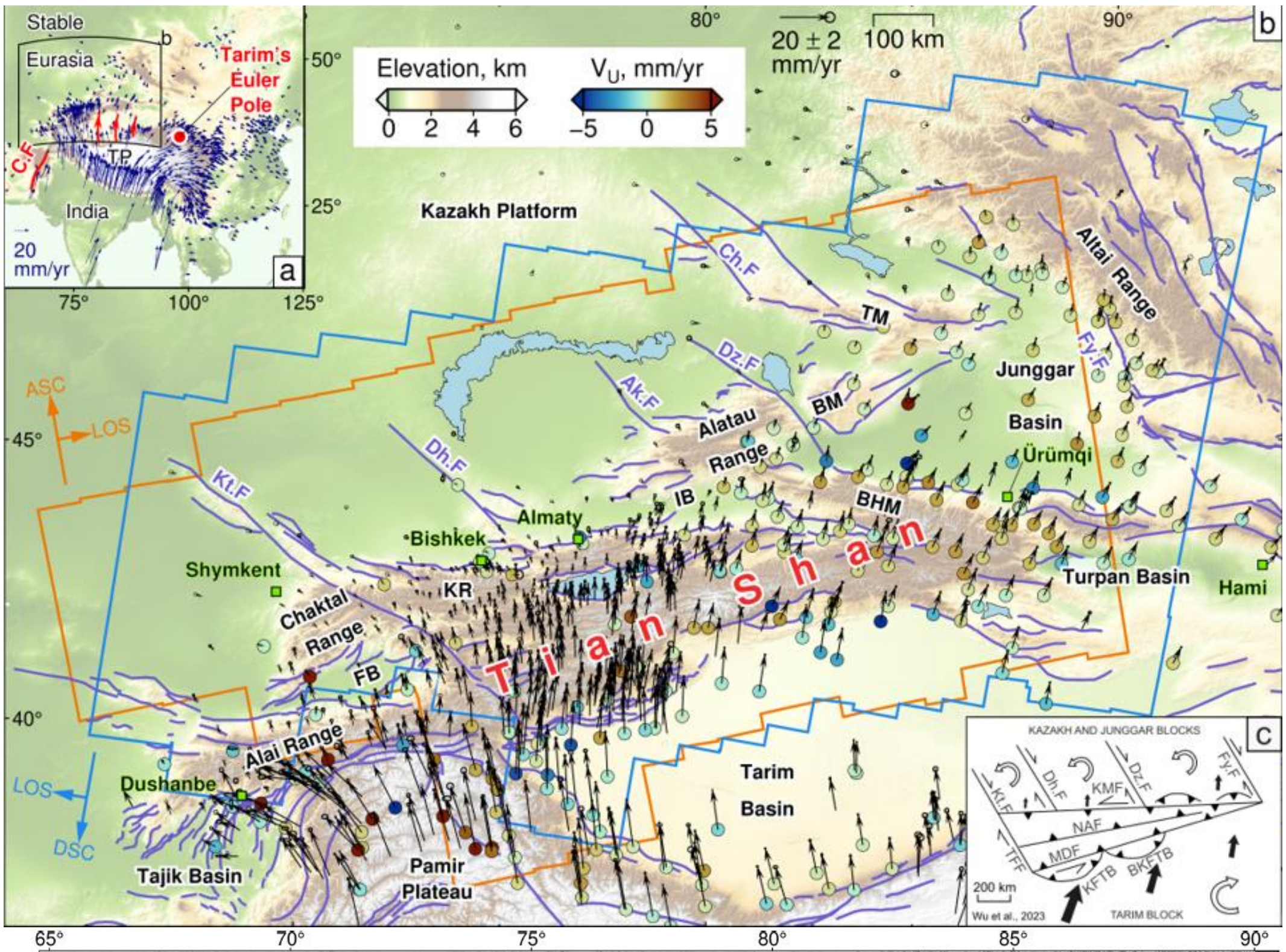

Stable
Eurasia
Tarim's
Euler
Pole
TP
India
C.F
20
mm/yr
a
50°
25°
75°
100°
125°
Elevation, km
0 2 4 6
V_U, mm/yr
−5 0 5
20 ± 2
mm/yr
100 km
80°
90°
b
Kazakh Platform
Ch.F
TM
Altai Range
Fy.F
Junggar
Basin
Dz.F
Ak.F
Alatau
Range
BM
Ürümqi
ASC
LOS
45°
Dh.F
IB
BHM
Kt.F
Almaty
Bishkek
Shymkent
KR
Tian Shan
Turpan Basin
Hami
Chaktal
Range
FB
40°
Alai Range
Dushanbe
LOS
DSC
Tajik Basin
Pamir
Plateau
Tarim
Basin
c
KAZAKH AND JUNGGAR BLOCKS
Kt.F
Dh.F
KMF
Dz.F
Fy.F
NAF
TFF
MDF
KFTB
BKFTB
200 km
Wu et al., 2023
TARIM BLOCK
65°
70°
75°
80°
85°
90°

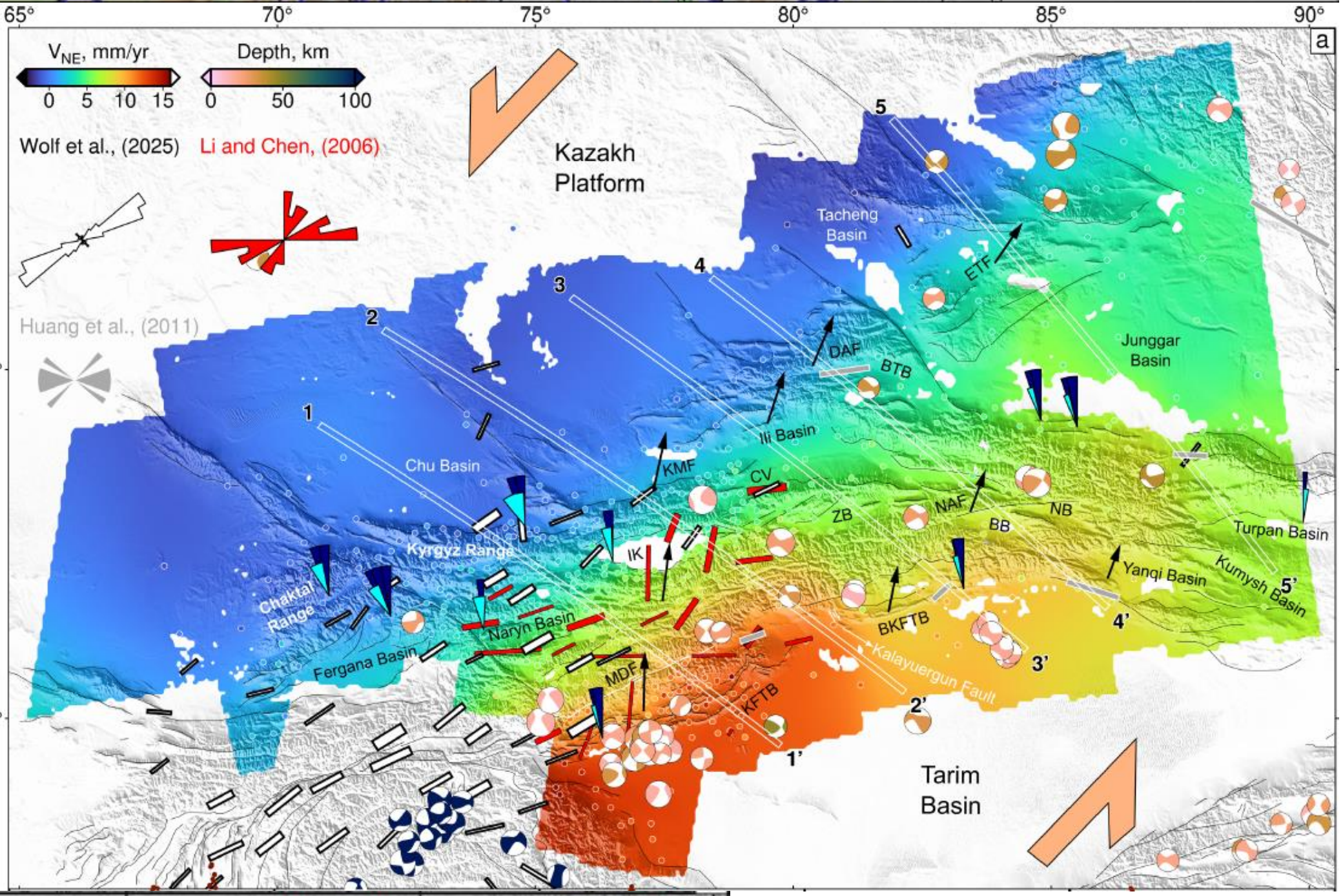

V_{NE}, mm/yr
0 5 10 15
Depth, km
0 50 100
a
Wolf et al., (2025)
Li and Chen, (2006)
Huang et al., (2011)
Kazakh
Platform
5
Tacheng
Basin
4
3
2
ETF
DAF
BTB
Junggar
Basin
45°
1
Chu Basin
KMF
Ili Basin
CV
ZB
NAF
BB
NB
Turpan Basin
IK
Kyrgyz Range
Chaktal
Range
Naryn Basin
BKFTB
Yanqi Basin
Kumysh Basin
5'
4'
3'
Fergana Basin
MDF
KFTB
Kalayuergun Fault
2'
40°
1'
Tarim
Basin

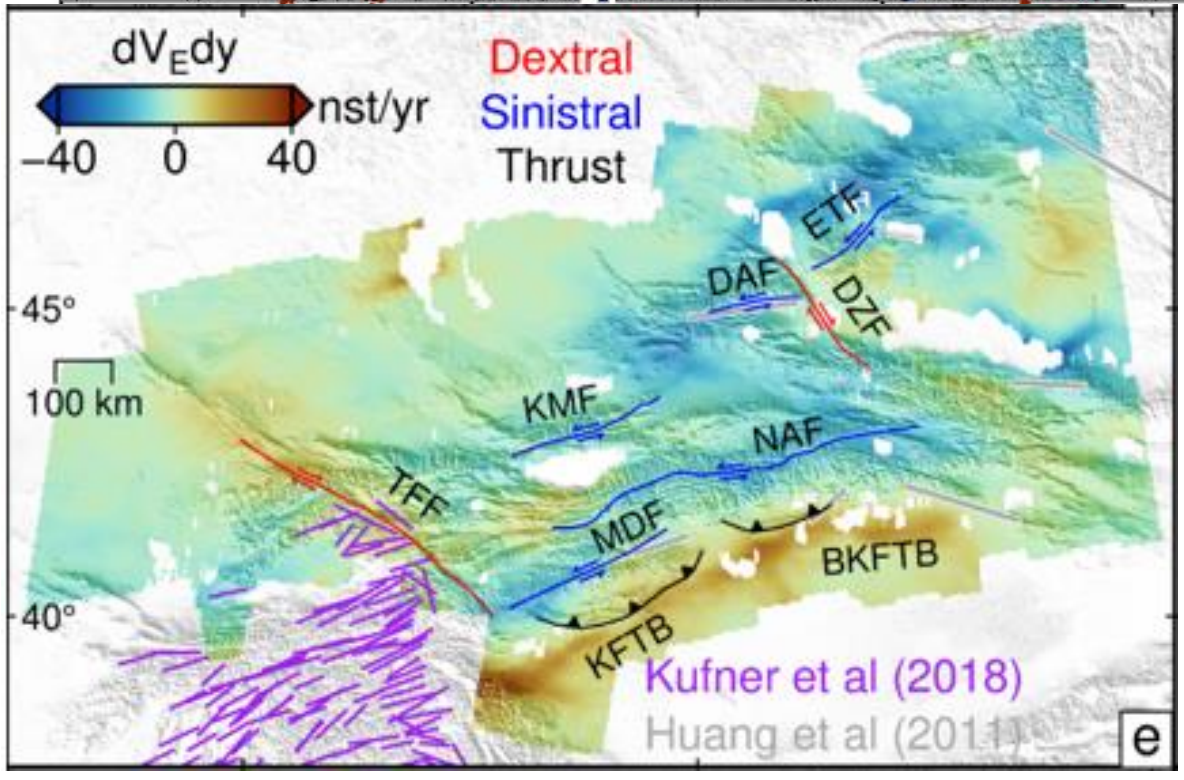

dV_Edy
nst/yr
−40 0 40
Dextral
Sinistral
Thrust
ETF
DAF
DZF
45°
100 km
KMF
NAF
TFF
MDF
BKFTB
KFTB
40°
Kufner et al (2018)
Huang et al (2011)
e

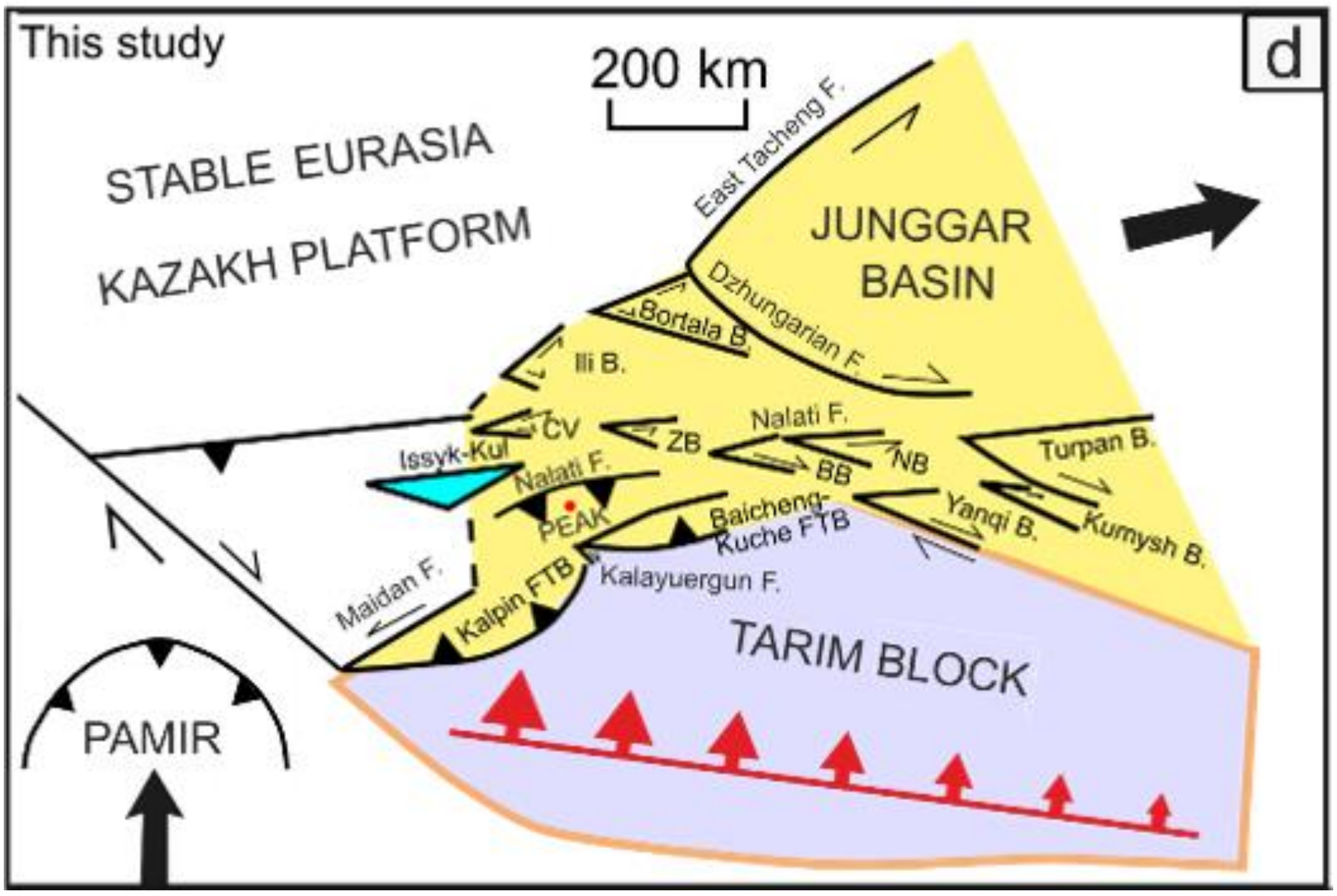

We have expanded on the discussion of the role of the TFF in the introduction and the results.

Introduction Lines 113-121:

"A total of over 200km of displacement has been recorded on the NW-trending right-lateral strike-slip Talas Fergana Fault (TFF) since the Permian (Fig. 1d), signifying its important role in accommodating deformation in the Tian Shan. However, different geological [19, 20] and geodetic [5, 16] slip rate estimates on this fault have invoked contrasting models of temporally variable fault kinematics [20, 21] and of block rotation [22, 23] to explain the discrepancy."

Section 2.1, Lines 219-326:

" About 2 mm/yr of dextral motion can be measured on the Karatau-Talas-Fergana Fault system, but it is distributed across ~200km on each side (Fig. 1d, 2e), consistent with the multiple branches on eitherside of the fault system (Fig. 2c). The longwavelength eastward velocity between the Karatau-Talas-Fergana Fault and the Aktas Fault, that partially induces the east velocity gradients on the Karatau-Talas-Fergana Fault (Fig. 2c,e), is not the best constrained due to the lack of GNSS stations in. this region. However, the relative eastward motion on the west of the Karatau-Talas- Fergana Fault and westward motion on the east of the Aktas Fault is consistent with anti-clockwise rotation of the block."

We think you meant the Talas-Fergana Fault is left-stepping. We discussed the left-stepping kink on the TFF as caused by the anticlockwise rotation of the Fergana Block in Lines 650-658:

"Anisotropy from Kufner et al. [54] highlights the NW-trending Talas-Fergana Fault (Fig. 2e), which is posited as a smaller-scaled, more-matured mirror reflection of the nascent NEtrending shear zone. It has a left-stepping kink against the Fergana Basin, likely due to the anticlockwise rotation of the Fergana Block driven by Pamir that co-produced the 100 km Tertiary displacement along the Talas-Fergana Fault [60](Fig 1a, Fig. 4b). "

This hypothesis would also explain the mismatch in fast axes orientations northwest of the nascent shear zone in the Kyrgyz Range in Lines 666-680:

"To illustrate how the flow fabric is in line with the NE-trending shear zone, we plot SKS shear-wave splitting measurements from Wolf et al. [56](Fig. 2j), reduced by 1- arc-degree block mean, and those from Huang et al. [53] and Li and Chen [55] on top of our NE-velocity (Fig. 3a). Stations from Wolf et al. [56] cover the part of the Tian Shan northwest of the shear zone. Fast axes generally trend 60–70◦N, oblique to the local range strike (90–100◦N) of the Kyrgyz Range in northwest Tian Shan (Fig. 1b). We interpret this mismatch as arising from either the orientation of the Terskey Suture from the Paleozoic (Fig. 2i) or from the anticlockwise rotational indentation of the Fergana Basin that co-produced the left-stepping kink on the Talas-Fergana Fault (Fig. 4a)."

We then explored the reason for the change of stress field that caused the TFF and Karakoram Fault to slow down in Lines 844-888:

"The direct contact between the Indian and Tarim lithospheric mantle could also explain the slowing down of the right-lateral slip rates on the Talas-Fergana and Karakoram Faults (Fig. 4b). The prominent right-lateral Talas-Fergana fault bounding the western Tian Shan, which had higher geological slip rates of 2.2–16 mm/yr [19, 20, 23], is currently geodetically moving at a much slower rate of ~1.5 mm/yr [16]. In our results, the change in east component of velocity across the Talas-Fergana Fault is ~2 mm/yr, which translates to ~2.3 mm/yr of slip rate distributed over a 200km zone (Fig. 2a,f), playing a less important role in accommodating shortening and lengthening than the NE-directed nascent shear zone identified in this study with over 10 mm/yr of velocity change distributed over 400 km of mountain range (Fig. 3a). Similarly, the Karakorum fault of the Western Tibetan Plateau, which had geological slip rates of 4–8 mm/yr [108–110], is geodetically inactive in the north and slowly slipping at 1.6–3.2 mm/yr in the south [111]. Cenozoic strike-slip motion on both the Talas-Fergana and Karakoram Faults commenced ~25 Ma, synchronous with the onset of exhumation and dextral strike-slip faulting along the eastern flank of Pamir and western Tarim [112, 113]. These right-lateral slips once played an important role in accommodating the Pamir's northward indentation that separated the Tarim and Tajik Basin [114] (Fig. 1b). That was when magmatism clustered in southern Tibet due to the delamination of the Tibetan lithospheric mantle, before the cold Indian lithospheric mantle shut off the heat from the asthenosphere at ~10Ma [101, 102]. As the Indian lithospheric mantle came into direct contact with the rigid deep lithospheric root of Tarim, like how the Indian and Asian lithospheric mantle are locking underneath Pamir today [54, 86], Pamir and the Western Tarim effectively became a united collisional front. They move north at similar rates, slowing the right-lateral strike-slip motion on the once-highly-active Talas-Fergana and Karakoram Faults. Hence, very limited strike-slip motion was recorded between Pamir and the Tarim since ~11Ma [115]."

We then discussed the changing roles of the parallel platform-cutting faults with respect to the changing stress field due to Tarim's rotation and the potential southward shift of Tarim's Euler Pole after the collision between Indian and Tarim's lithosphere in Lines 890-926:

"The roles of the NW-striking platform-cutting dextral faults (KtF, DhF, AkF, DzF,and ChF in Fig. 1b,c) likely also diminished with the changing stress field due to the rotation of the Tarim Basin. It was estimated that the Tarim Basin has rotated clockwise by 7±2.5◦ since the late Cenozoic, assuming an Euler Pole near Hami [14, 99] (Fig. 1b and 4b). If the Euler Pole were indeed around Hami near the onset of Tian Shan's reactivation, the stress field felt by the Kazakh Platform would have been northward compression (Fig. S19a,b), which promotes the development of the parallel Nw-trending platform-cutting strike-slip faults. These dextral faults likely formed trending ~N30◦E [116] and accommodated shortening by anticlockwise block rotation [22, 23, 117], hence rotating themselves to be NW-trending. We do not think Tarim's Euler Pole has always been in the Qilian Shan, as if so, the platform-cutting dextral faults could not have developed into the magnificent structures they are, and the nascent shear zone that we describe would have had more time to mature (Fig. S19c). Hence,

it is likely that the southward shift of Tarim's Euler Pole to the Qilian Shan, due to the India-Tarim lithospheric contact around 10 Ma[68, 100, 101](Fig. 4b), rotated the stress field to point northeasterly (Fig.3a). This new stress field bypasses the platform-cutting dextral faults. Hence the present-day strain rate on these structures becomes negligible (Fig. 1d and 2a,c). The Talas-Fergana Fault is still active [23] because of the differential north-south shortening rates in the Tian Shan that still exist north of Pamir and Tarim, respectively [5]. The Dzhungarian Fault is still active [22, 117] due to its new role in facilitating the eastward translation of the Junggar Basin (Section 2.4)."

L615: Figure 4b is discussed after 4c, consider rearranging the subfigures in this figure.
Thanks for pointing the sequencing error on the figure panels. We have rearranged the panels and made sure the panels are referred to in sequence.

L655: Has this lengthening mechanism of the southeast half of the Tian Shan been introduced/discussed earlier in the text? Why does it only affect the Southeastern part, and not the whole area covered by the newly found shear zone?
Thanks for your great question, which prompted us to perform some new analysis. We added a comparison between the GNSS vector azimuths and the unit vector azimuths predicted in the sense of Tarim's rotation across the Tian Shan (Fig.2a,b). We found that the misfits are near-zero within the triangular wedge to the southeast of the shear zone, but significantly larger to the northwest of the shear zone. This contrast suggests that the southeast half of the Tian Shan is escaping eastward under the control of the Tarim's rotation. The northwest half of the Tian Shan is absorbing mostly shortening from indentation from both the Pamir and the Tarim and escaping to the west (Fig. S18b). We introduce and discuss this lengthening mechanism in Section 2.4 (Lines 929-1069), and change our subsection heading to "A Hierarchical Triangular Wedge Model for Crustal Escape" to make the point clearer. In Figure S18b (bottom left here), we show that the triangular wedge with easterly motion falls short of the fan-shaped wedge swept by Tarim's rotational stress field. We believe that is due to the shortening accommodated through eastward crustal escape through the hierarchical triangular wedge model (Fig.4d).

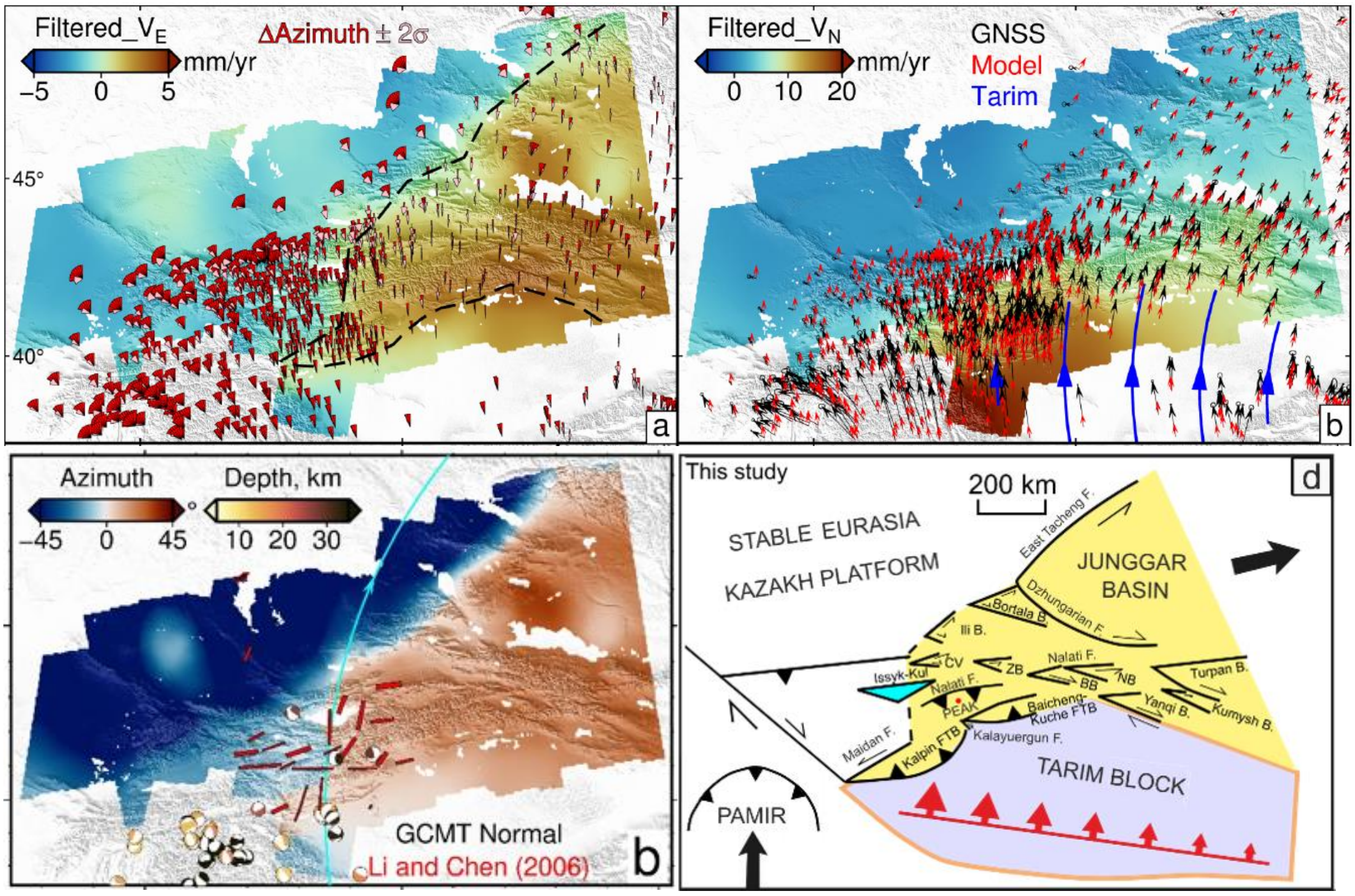

L665-677: Can these important statements be backed up with more references, e.g. works from tomography? Is it clear that the Junggar Basin is significantly weaker than the Kazakh platform?

We added references on

The geology of Kazakh Platform:

Degtyarev, K., Yakubchuk, A., Tretyakov, A., Kotov, A., Kovach, V.: Precambrian geology of the Kazakh Uplands and Tien Shan: An overview. Gondwana Research 47, 44–75 (2017) https://doi.org/10.1016/j.gr.2016.12.014

Tomography revealing the contact between Tian Shan and the Kazakh Platform:

Sychev, I.V., Koulakov, I., Sycheva, N.A., Koptev, A., Medved, I., Khrepy, S.E., Al-Arifi, N.: Collisional Processes in the Crust of the Northern Tien Shan Inferred From Velocity and Attenuation Tomography Studies. Journal of Geophysical Research: Solid Earth 123, 1752–1769 (2018) https://doi.org/10.1002/2017JB014826

Tomography under the whole Tian Shan:

Ziqiang, L., Gao, H., Lei, J., Yang, X., Rathnayaka, S., Li, C.: Crustal and Upper Mantle Structure of the Tien Shan Orogenic Belt From Full-Wave Ambient Noise Tomography. Journal of Geophysical Research: Solid Earth 124, 3987–4000 (2019) https://doi.org/10.1029/2019JB017387

Structure under the Junggar Basin and its rigid body rotation:

Zhu, M., Liang, Z., Wang, X., Wang, R., Yu, Y., Xiao, L., Wei, L.: Mesozoic strike-slip fault system at the margin of the Junggar Basin, NW China. Journal of Structural Geology 175, 104950 (2023) https://doi.org/10.1016/J.JSG.2023.104950

How material strength controls the spacing between the strike-slip faults:

Jiao, L., Tapponnier, P., Donz´e, F.V., Scholt`es, L., Gaudemer, Y., Xu, X.: Discrete Element Modeling of Southeast Asia's 3D Lithospheric Deformation During the Indian Collision. Journal of Geophysical Research: Solid Earth 128 (2023) https://doi.org/10.1029/2022JB025578

How material strength controls the spacing between the strike-slip faults:

Zuza, A.V., Yin, A., Lin, J., Sun, M.: Spacing and strength of active continental strike-slip faults. Earth and Planetary Science Letters 457, 49–62 (2017) https://doi.org/10.1016/J.EPSL.2016.09.041

We provide a clearer contrast between the hierarchical triangular wedge model and the bookshelf model in lines 1033-1069:

"The triangular wedge model contrasts with the bookshelf model traditionally employed to describe the parallel dextral faulting in the Kazakh Platform (Fig. 1c). The bookshelf model predicts the strike-slip motion on parallel new faults generated in a pristine and rigid material under a compressional stress governed by Anderson's theory of faulting [127]. Our hierarchical wedge model predicts the

sense of strike-slip component motions on pre-existing faults in response to a changing stress field. This model does not predict slip rates or the sense of dip-slip component motion on the faults. The bookshelf rotation was likely the dominant lengthening mechanism when Tarim had an Euler Pole near Hami and exerted a northward compressional stress through the Kazakh Platform. After the southward shift of Tarim's Euler Pole, the eastward crustal escape through the conjugate component strike-slip motions along the various-sized triangular wedges within the southeastern Tian Shan became a more dominant lengthening mechanism. The different styles of tectonic response come from the different rheologies characterising the northwest and southeast halves of the Tian Shan orogeny. To the north of the NE-trending shear zone, the Tian Shan is pushing against the Kazakh Platform where the crystalline basement [68, 128, 129] promotes brittle faulting along parallel strike-slip faults [130, 131]. To the southeast of the shear zone, except the Junggar Basin, which acts mostly as a rigid block [79], the weaker Tian Shan material [132] can deform more continuously, distributing itself to the east where there is no hard boundary [38]. Therefore, our observation and conceptual model are also consistent with the dynamic models that suggest how stress orientation and rheological structures together control the deformation fields [95]."

L719/720: Can you provide a reference to a similar finding that includes a modeling attempt?

Thank you for your suggestion. We have added the following reference in line 1115:

"This large-scale signal is likely a result of glacial isostatic adjustment due to the ice unloading [139]."

Li, Y., Liu, M., Wang, Q., Cui, D.: Present-day crustal deformation and strain transfer in northeastern Tibetan Plateau. Earth and Planetary Science Letters 487, 179–189 (2018) https://doi.org/10.1016/j.epsl.2018.01.024

L729: I cannot identify those red triangles in Figure 5e

Apologies for the mistake. We have added the red triangles to Figure 5f.

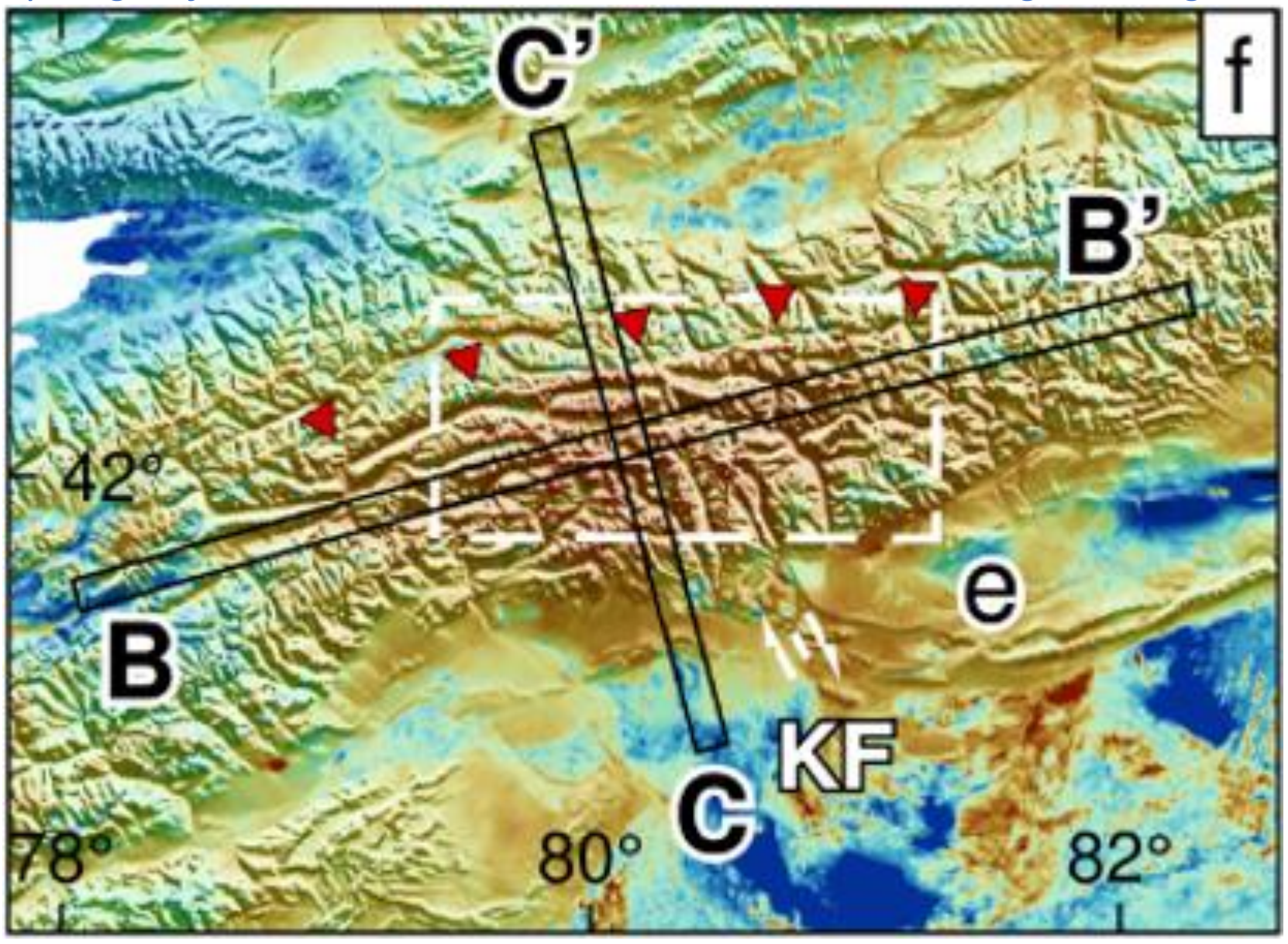

L746: A figure reference might help here to sort out the geographic features or mark those in 5f? Actually Kalpin is only labeled in figure 4c, Kuqa nowhere? It is a bit hard to follow you until L766.

Sorry for the confusion. We have labelled Kalpin and Baicheng-Kuche Fold-and-Thrust Belts, the Kalayuergun Fault relative to the peak now in Figure 1de, 2egij, 3a, 4d and 5a.

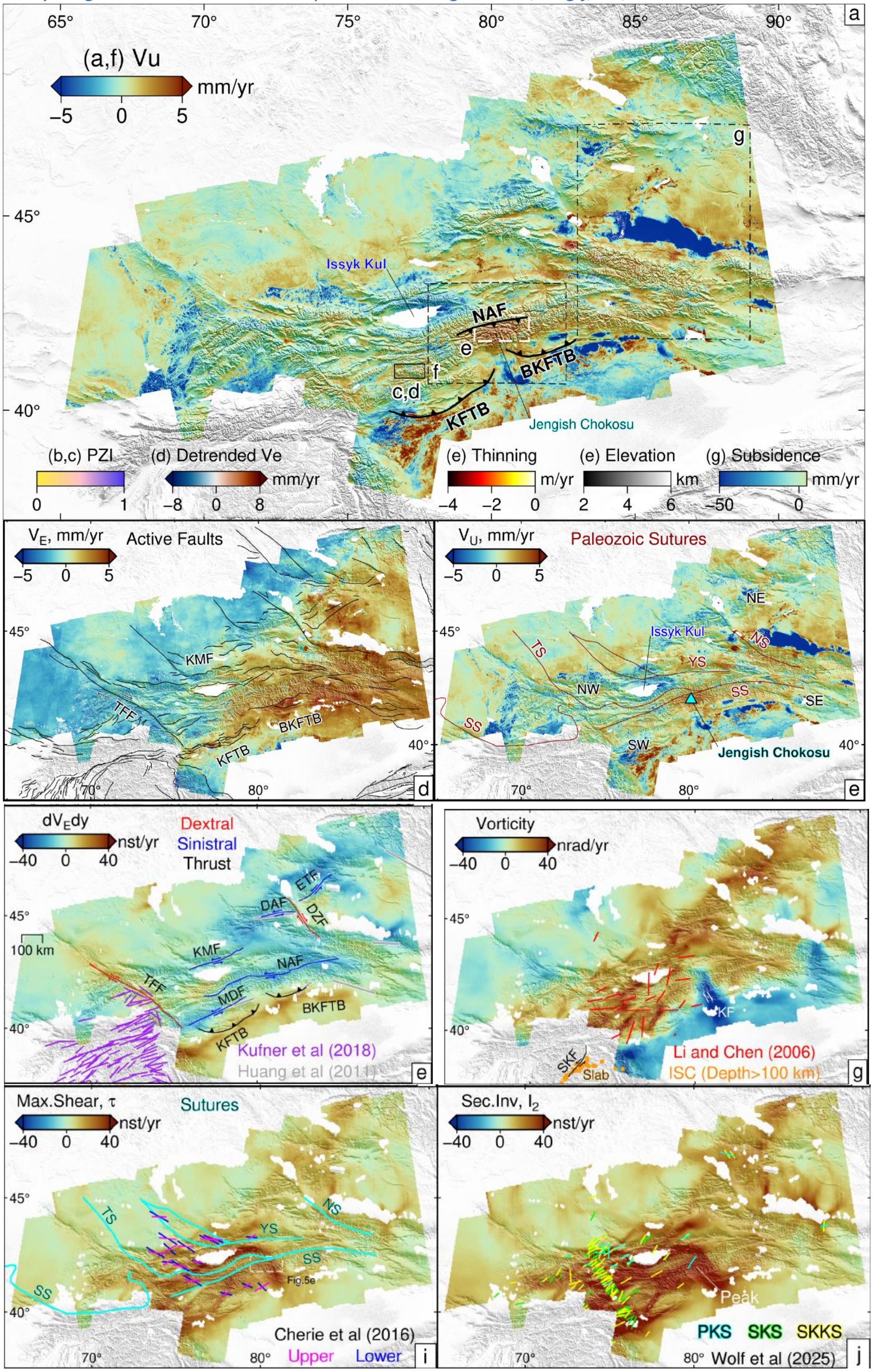

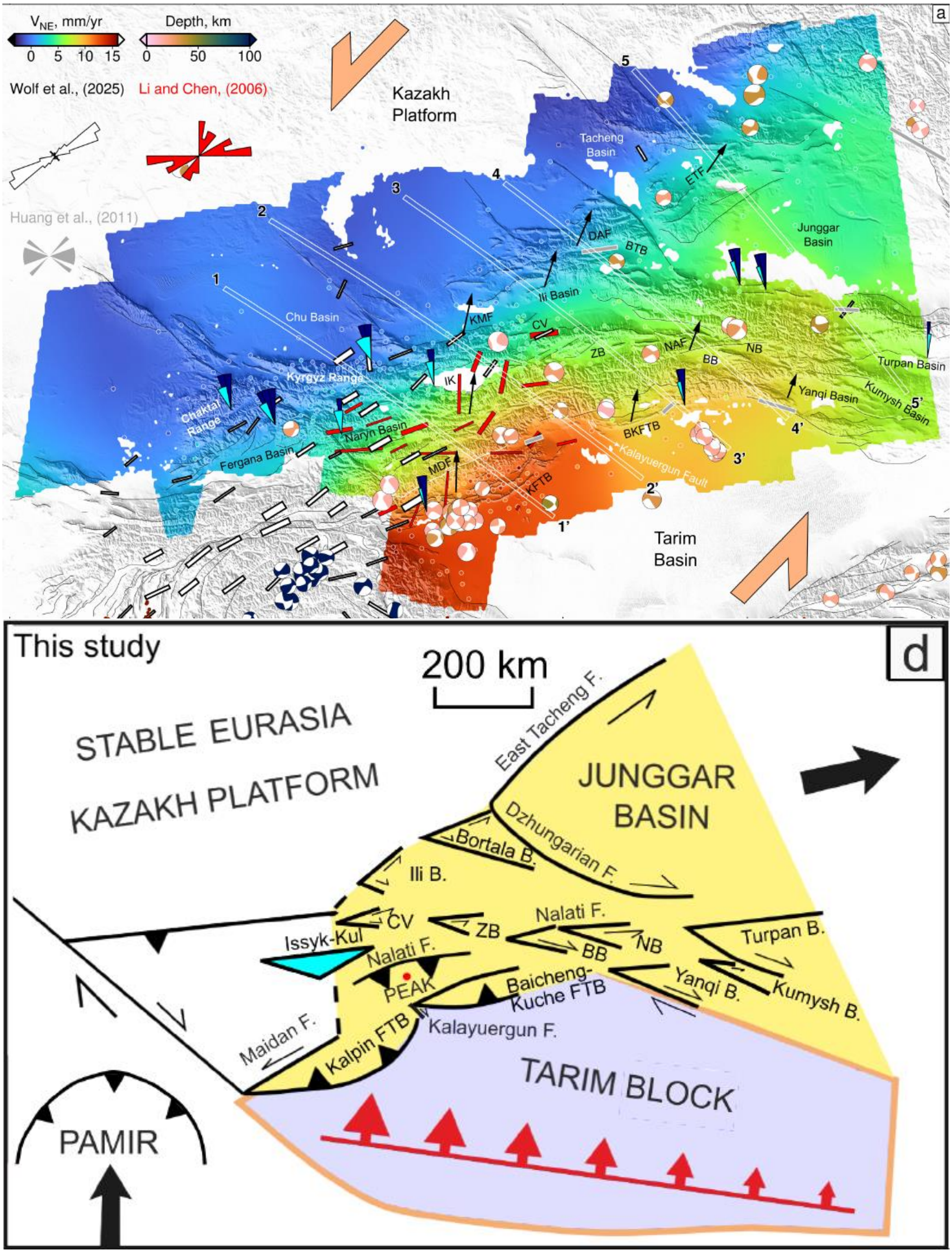

L768: Similar to the ice mass loss, it would be good to refer to a case study where this has been effectively modeled, e.g. Californian Central Valley (https://doi.org/10.1029/2019JB018490, but maybe not a key publication)

Thank you for your suggestion. We have added the following reference in line 1189:
"Further work is needed to model the uplift relative to the rate of groundwater extraction [149]."

Carlson, G., Werth, S., Shirzaei, M.: A novel hybrid GNSS, GRACE, and InSAR joint inversion approach to constrain water loss during a record-setting drought in California. Remote Sensing of Environment 311, 114303 (2024) https://doi.org/10.1016/j.rse.2024.114303

L812: Can you explain the meaning of the PZI, what is 0 what is 1?

Apologies about the confusion. We have added the meaning of PZI to the figure caption for Figure 5b:

"*A PZI near 1 indicates that permafrost is expected nearly everywhere in a pixel. A PZI near 0 means permafrost presence is only expected under the most favourable conditions."*

According to Gruber 2012, PZI is a preferred way of communicating a model of "permafrost extent" which is scaled from point data and not easily calibrated. Hence the author purposefully advised against interpreting PZI quantitatively like a likelihood or probability of permafrost presence. Instead, the author advised interpreting the PZI with respect to local conditions (shading, shadow, cover, etc). The modelled permafrost extent model is scaled from point observations of mean surface air temperature and the type of permafrost at the observation points (continuous permafrost = above 90% coverage, isolated patches = below 10% coverage). This scaled model at the coarse resolution of temperature data is further downscaled using the SRTM DEM based on how temperature drops with elevation. Ruggedness and landcover are then factored in stochastically as uncertainties, which is presented in the dataset as a green fringe outside of the contour of PZI=0, and not included in our figure.

L815: I cannot find the blue triangles. The gray shading in the profiles is not explained.

Apologies for the mistake. We have now replaced the blue triangles with a cyan dotted line representing the Southern Tian Shan Suture, which coincides in space with the drainage divide, and also the South Tian Shan Suture.

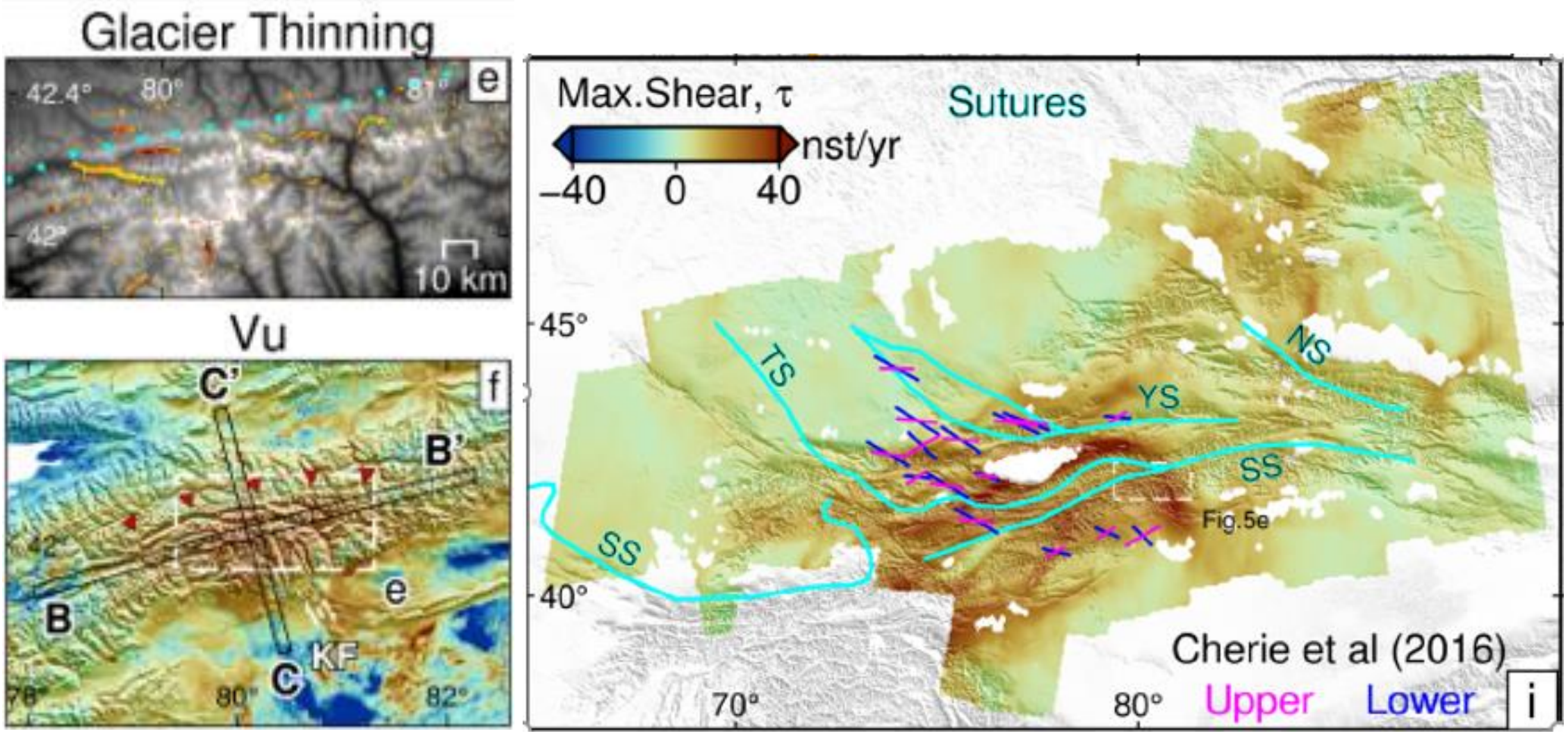

We've added the explanation of gray shading as topographic profiles from SRTM in the caption of Figure 5: "Grey shading in profiles B–B' to E–E' show topography profiles from SRTM [37]."

L829: Similar to above this statement could be backed up with some key literature where one can learn about the modeling strategy of such features.

Thanks for your request. After more thorough literature review, we are not sure if a lithospheric scale rebound is required. So we removed the sentence discussing about how we can obtain lithospheric thickness from modelling such features, and added the following citation at the end of the paragraph to direct the readers to modelling of uplift from groundwater depletion and from glacial isostatic adjustment instead.

Lines 1243-1246:
"Both uplift signals result from a combination of tectonic and nontectonic processes, requiring models coupling multiple mechanisms to explain and investigate further [150, 151]."

Amos, C.B., Audet, P., Hammond, W.C., B¨urgmann, R., Johanson, I.A., Blewitt, G.: Uplift and seismicity driven by groundwater depletion in central California. Nature 2014 509:7501 509, 483–486 (2014) https://doi.org/10.1038/nature13275

Dietrich, R., Ivins, E.R., Casassa, G., Lange, H., Wendt, J., Fritsche, M.: Rapid crustal uplift in Patagonia due to enhanced ice loss. Earth and Planetary Science Letters 289, 22–29 (2010) https://doi.org/10.1016/j.epsl.2009.10.021

L875: Since this is the discussion section it would be nice to better embed your findings into existing literature. In the result section you do this by (I think) reporting on similar findings. Maybe you could elaborate a bit on the process here (compaction due to pore pressure, or creep, or common compaction). For example I still wonder whether one can discriminate between signals caused by loss of permafrost and signals caused by simple gravitational compaction of unconsolidated sediments. Is the signal not also observed at low-latitude regions without permafrost but gravelly slopes?
Thanks for your suggestion. We had added the following literature, and discussed why we think the feature is related to permafrost degradation, in particular, solifluction and/or frost creep, possibly facilitated by basal sliding.

"At the kilometre scale, our detrended east velocities reveal rapid down-slope creep motions that have a horizontal component up to 8 mm/yr on both sides of northsouth- trending ridges in the permafrost zone. The rate of movement is slower than that of typical slow landslides [152]. The horizontal components of the motion also differentiate the mechanism from simple gravitational compaction of unconsolidated sediments [153, 154]. What we observed is most likely solifluction and/or frost creep [134], possibly facilitated by basal sliding [137], highlighting the rapid rate of permafrost degradation under a changing climate. Potentially similar rates of motion also occur on the north and south slopes of east-west-trending ridges, which we are currently missing due to the near-polar orbit and the two observation geometries of Sentinel-1 satellites alone."

Brain, M.J.: Past, Present and Future Perspectives of Sediment Compaction as a Driver of Relative Sea Level and Coastal Change. Current Climate Change Reports 2, 75–85 (2016) https://doi.org/10.1007/s40641-016-0038-6

Teshebaeva, K., Echtler, H., Bookhagen, B., Strecker, M.: Deep-seated gravitational slope deformation (DSGSD) and slow-moving landslides in the southern Tien Shan Mountains: new insights from InSAR, tectonic and geomorphic

analysis. Earth Surface Processes and Landforms 44, 2333–2348 (2019) https://doi.org/10.1002/esp.4648

Daout, S., Sudhaus, H., Kausch, T., Steinberg, A., Dini, B.: Interseismic and Postseismic Shallow Creep of the North Qaidam Thrust Faults Detected with a Multitemporal InSAR Analysis. Journal of Geophysical Research: Solid Earth 124(7), 7259–7279 (2019) https://doi.org/10.1029/2019JB017692

Watine, H., Daout, S., Lav´e, J., Doin, M.P.: Downslope solifluction movements and permafrost degradation in the northeastern Qinghai-Tibetan Plateau revealed by InSAR. Remote Sensing of Environment 329, 114926 (2025) https://doi.org/10.1016/J.RSE.2025.114926

L894: Is there any literature that could be suggested for further reading?

Thanks for your suggestion. We had added the following literature in lines 1307-1316:

"At between kilometre to hundreds-of-kilometre scales, vertical velocities exhibit long-wavelength uplift signals in response to rapid deglaciation and groundwater extraction that might be overprinting signals of, or in the case across the highest peak interacting with, the rate and style of mountain building and faulting activities, providing an opportunity to study the interplay between tectonic, climatic and anthropogenic activities [139, 141, 149, 155]."

Pintori, F., Serpelloni, E., Gualandi, A.: Common-mode signals and vertical velocities in the greater Alpine area from GNSS data. Solid Earth 13, 1541–1567 (2022) https://doi.org/10.5194/se-13-1541-2022

Wu, P., Steffen, R., Steffen, H., Lund, B.: Glacial Isostatic Adjustment Models for Earthquake Triggering. In: Steffen, H., Olesen, O., Sutinen, R. (eds.) Glacially-Triggered Faulting, pp. 383–401. Cambridge University Press, Cambridge (2021)

Carlson, G., Werth, S., Shirzaei, M.: A novel hybrid GNSS, GRACE, and InSAR joint inversion approach to constrain water loss during a record-setting drought in California. Remote Sensing of Environment 311, 114303 (2024) https://doi.org/10.1016/j.rse.2024.114303

Liu, S., J´onsson, S.: Glacial Isostatic Uplift in the Tibetan Plateau and Surroundings. Geophysical Research Letters 52, 2025–114899 (2025) https://doi.org/10.1029/2025GL114899

L897-905: While I find this paragraph somewhat generic I suggest to better point out how large InSAR rate maps can be used to discuss deeper processes instead of focusing on single faults, e.g. by comparing to shear wave splitting data and the like which opens new opportunities. (However this should be discussed in the context of how much InSAR there is left in the long-wavelength, or how much it is dominated by GNSS – where critics could ask why we need InSAR…) A second line of thought I suggest to think about: Do all large strike-slip systems evolve over time like we see now in the Tian Shan, or is this coincidence and mostly related to a change in the stress field over time? Would you find similar old topography along other large strike-systems world wide?

Thank you for the questions.

Indeed, we had challenges associated with mosaicking InSAR LOS velocities across the Tian Shan due to the lack of GNSS control in the Kazakh Platforms, especially on the east of the Talas Fergana Fault. We have highlighted limitations of InSAR and importance of GNSS stations in view of mosaicking artefacts along track boundaries and long-wavelength anomaly east of the Talas Fergana Fault.

Lines 223-323:
"The longwavelength eastward velocity between the Karatau-Talas-Fergana Fault and the Aktas Fault, that partially induces the east velocity gradients on the Karatau-Talas-Fergana Fault (Fig. 2c,e), is not the best constrained due to the lack of GNSS stations in this region"

We highlight the importance of GNSS for fixing the long wavelength ramps in InSAR in the Discussion and encourage the collaborative efforts between the InSAR and GNSS communities to improve the spatial distribution of GNSS data for the future referencing of InSAR at continental scales.

Lines 1327-1345:
"However, high-quality GNSS velocities will still be essential for mosaicking InSAR velocities over multiple tracks. It is important that we have a good distribution of more than 4 3-dimensional GNSS data at locations we expect InSAR to be coherent to anchor the planar ramp of each InSAR frame. Imperfect referencing due to the lack of GNSS constraints, for example in non-deforming regions around the Karatau-Talas-Fergana Fault and within the Tarim Basin (Fig. 1b, Fig. S12), led to strain artefacts along InSAR track boundaries (Fig. 2c). Therefore, it would be desirable for the InSAR and GNSS communities to collaborate strategically, in view of the footprint of radar satellites, to improve the spatial distribution of GNSS data acquisitions. This step is crucial to referencing the next-generation continental-scale high-resolution velocity maps [87]."

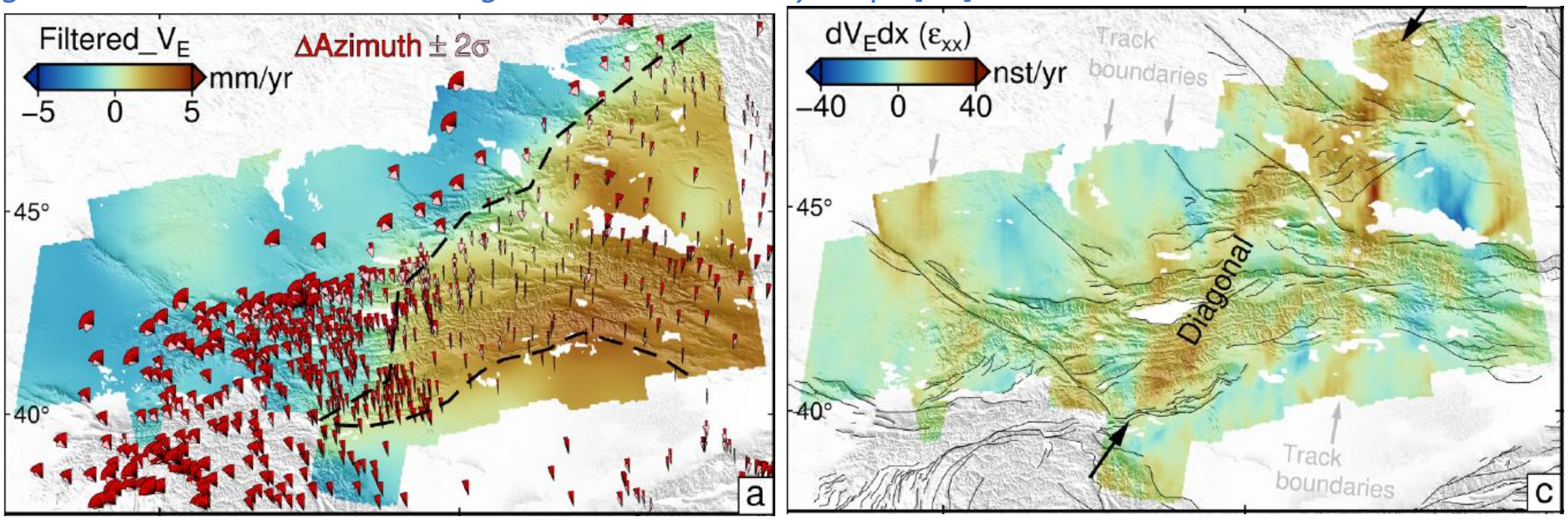

L929: Provide a reference to LiCS system.
We have added the reference to the LiCS system in Line 1380:

“We processed 8.5 years of Sentinel-1 InSAR data acquired between October 2014 to March 2023 to yield line-of-sight (LOS) velocities over 90 frames as defined in the Looking into Continent from Space (LiCS) system [157]”

Lazeck´y, M., Spaans, K., Gonz´alez, P.J., Maghsoudi, Y., Morishita, Y., Albino, F., Elliott, J., Greenall, N., Hatton, E., Hooper, A., Juncu, D., McDougall, A., Walters, R.J., Watson, C.S., Weiss, J.R., Wright, T.J.: LiCSAR: An Automatic InSAR Tool for Measuring and Monitoring Tectonic and Volcanic Activity. Remote Sensing 12(15), 2430 (2020) https://doi.org/10.3390/rs12152430

L936: I would exclude Fig 1c-f from the main text and put it to the supporting material.
We have moved the Figure 1c-f from the main text to the supporting material.

L972: mention that SNAPHU is a phase unwrapping algorithm
We have added the description to line 1401 and line 1429:

“All interferograms were unwrapped using the SNAPHU phase unwrapping algorithm [160, 161] incorporating modified Goldstein filtering [162].”

“To choose the reference window for each frame, we combined four proxies to select a 5 ×5km2 window with the highest pixel coverage, highest average coherence, lowest terrain ruggedness, and in the largest connected component obtained through the SNAPHU phase unwrapping algorithm [160, 161] (Text S3, Fig. S6).”

L955ff: See my comment to the figure caption of figure 1: I do not understand whether this is the average number for all tiles or something else?
We have now explained in the supporting Figure S1 that these numbers are average number for all frames.

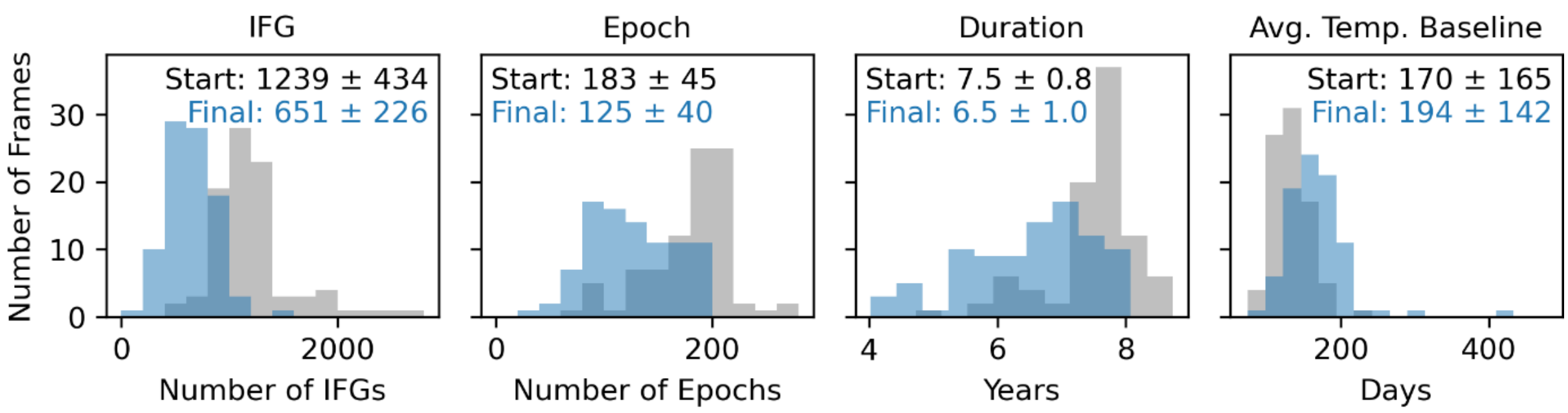

Figure S2: The per-frame statistics of number of interferograms, epochs, time span and average temporal baselines of interferograms before and after the quality control, through the improved LiCSBAS11 (Text S2) and newly developed LiCSBAS132 (Text S4) steps during the time series analysis.

References: The references sometimes lack capitalization, which (in latex) can be solved by using double brackets (title = {{…}})
Thank you pointing out the reference issue. We have added the double brackets to ensure correct capitalization.

Supporting Text

L35-39: Fix reference error

The references are missing?

Thank you pointing out my mistake. I have fixed the references in the supporting text. The references now display correctly, and all the references are included in the bibliography of the main text.

"(3) For phase unwrapping, we used the Statistical-cost, Network-flow Algorithm for Phase Unwrapping (SNAPHU) (Chen and Zebker, 2001, 2002) to perform the core phase unwrapping procedure in a processing chain incorporating modified Goldstein filtering (Goldstein and Werner, 1998). To facilitate unwrapping, we combined this step with atmospheric correction and removed atmospheric phase delays given by Generic Atmospheric Correction Online Service (GACOS) (Yu et al., 2017, 2018a, 2018b) before unwrapping, if it reduces the standard deviation of phases in the original interferogram (Lazecky et al., 2022). We retained the connected components by-products of the SNAPHU phase unwrapping to facilitate reference window selection (Text S3) and unwrapping mistake correction (Text S4) after preliminary time series analysis."

Reviewer #1 (Remarks on code availability):

I have used this code before, but have not checked the improvements described in the supporting material in detail. The improvements, however, sound very helpful and reasonable. The older version of the software is easy to install and use, comes with test cases, manuals, READMEs etc. The code is open and can be modified to one's own liking. I would appreciate if the authors make an effort to also provide an updated manual and check whether the LiCSAR data base contains all necessary metafiles to run the code without error (I, for example, often run into problems with the baseline information for some reason).

Thank you for pointing out the issue with the sometimes missing baseline files. We are aware of this problem, and have tried to minimise the number of LiCSAR frames with missing baseline information. However, this problem still occurs in a number of LiCSAR frames. The baseline does not affect data processing and is used by LiCSBAS to generate baselines plot and optionally estimate DEM error. The latter is not applied for Sentinel-1 data in this study due to the tight orbital tube of the satellite mission by design. As of now, non-existing or incomplete baselines file would only lead to auto-generated dummy baselines file, see LiCSBAS02_ml_prep.py lines 522-533. We will continue to experiment ways to tackle the missing baseline issue for improved user experience.

As you requested, we have now written an updated manual to the new developments in this study and added the documentation to the README page of https://github.com/qi-ou/LiCSBAS, *which is linked from the "dev" branch of* https://github.com/comet-licsar/LiCSBAS.

We also archive scripts used for mosaicking, decomposing and error analysing the InSAR velocities derived from LiCSBAS in the Modera repository https://github.com/qi-ou/Modera.

Reviewer #2 (Remarks to the Author):

This manuscript presents a compelling study of the active deformation mechanisms within the Tianshan orogenic belt. Based on dataset of Sentinel-1 InSAR and GNSS velocities, this study revealed a previously unrecognized, through-going northeast-trending shear zone in the entire Tianshan in response to the rotation of the Tarim basin. The key finding provides a novel framework for understanding the intracontinental evolution of this orogen. The manuscript is well-written, and the conclusions are well-supported by the data. However, several points require clarification to strengthen the arguments and enhance the manuscript's clarity and impact before consideration of publication.

Thank you for your review and your recognition of the novel framework for understanding the intracontinental evolution of the Tian Shan orogen provided by our study. We have strengthened the arguments and clarified the manuscripts according to your suggestions.

(1) The term "nascent shear zone" is central to the manuscript. While the geodetic and seismological evidence for distributed shear is convincing, direct evidence for its incipient nature is somewhat circumstantial. The authors should strengthen this argument by explicitly discussing what geological or geomorphological evidence would be expected for a newly developing shear zone. Besides, further elaborating on the comparison to other nascent fault systems is beneficial to clarifying if the current strain distribution and fault segmentation patterns of the research area are diagnostic of an early evolutionary stage.

Thanks for your question. We now placed our hypothesis with respect to Tian Shan's tectonic history since the Paleozoic and explained geomorphic features that we couldn't explain with present-day stress field in view of the locations of the Paleozoic Sutures and the Cenozoic tectonic arrangement since the indentation of Pamir. In this way, we tease out geomorphic features that reflect past tectonic episodes from the geodetic and seismological evidence that reflects present-day stress field. We also proposed the contact between the Indian and Tarim lithospheric mantle as a reason for the recent change of Tarim's Euler Pole around 10 Ma that caused the incipient nature of the NE-oriented shear zone that we describe. We cite the European Alps as a parallel system with the Adriatic block, driven by the NNW push from the African Plate, indenting first into western Alps when the Euler Pole was in Spain, and then shearing the eastern Alps now with an Euler Pole in the Po Basin, creating a dextral strike-slip Peri-Adriatic Lines against the Alps.

We summarise our evidence in lines 767-775:

"Thus, based on the (1) alignment of vorticity field and anisotropy results with the stress field, (2) the distributed nature of shear strain and strike-slip seismicity, and (3) the lack of optimally-oriented mature faults to accommodate the shear strain, we have possibly captured the early days of the development of a new shear zone across the Tian Shan, following the successive northward propagation of the development of large-scale sinistral strike-slip faults across the Tibetan Plateau [92, 93]."

In terms of geomorphic evidence, we note a mismatch between the strikes of the mountain ranges with the shear-wave splitting fast axes directions in the Kyrgyz Range northwest of the shear zone. We interpret the shear-wave splitting trends as the orientation of the present-day ductile flow fabric, which indicates the current stress field. We interpret the strikes of the mountain range to either be inherited from the tectonic episodes that formed the Paleozoic suture zones, or oroclinally bent from the clockwise rotation of the Fergana Block, both predating the NE-trending stress field we observe today, suggesting the current stress field is quite recent.

We also looked into paleomagnetic evidence. However, all reported vertical-axes rotations so far are found on the edges of the Tian Shan mountain belt and are interpreted by their authors as showing "local small block rotations" only. We use this interpretation to support the hierarchical triangular wedge model where we expect crustal wedges of various scales to translate and rotate through conjugate strike-slip component motion on basin bounding faults under the dominant stress field.

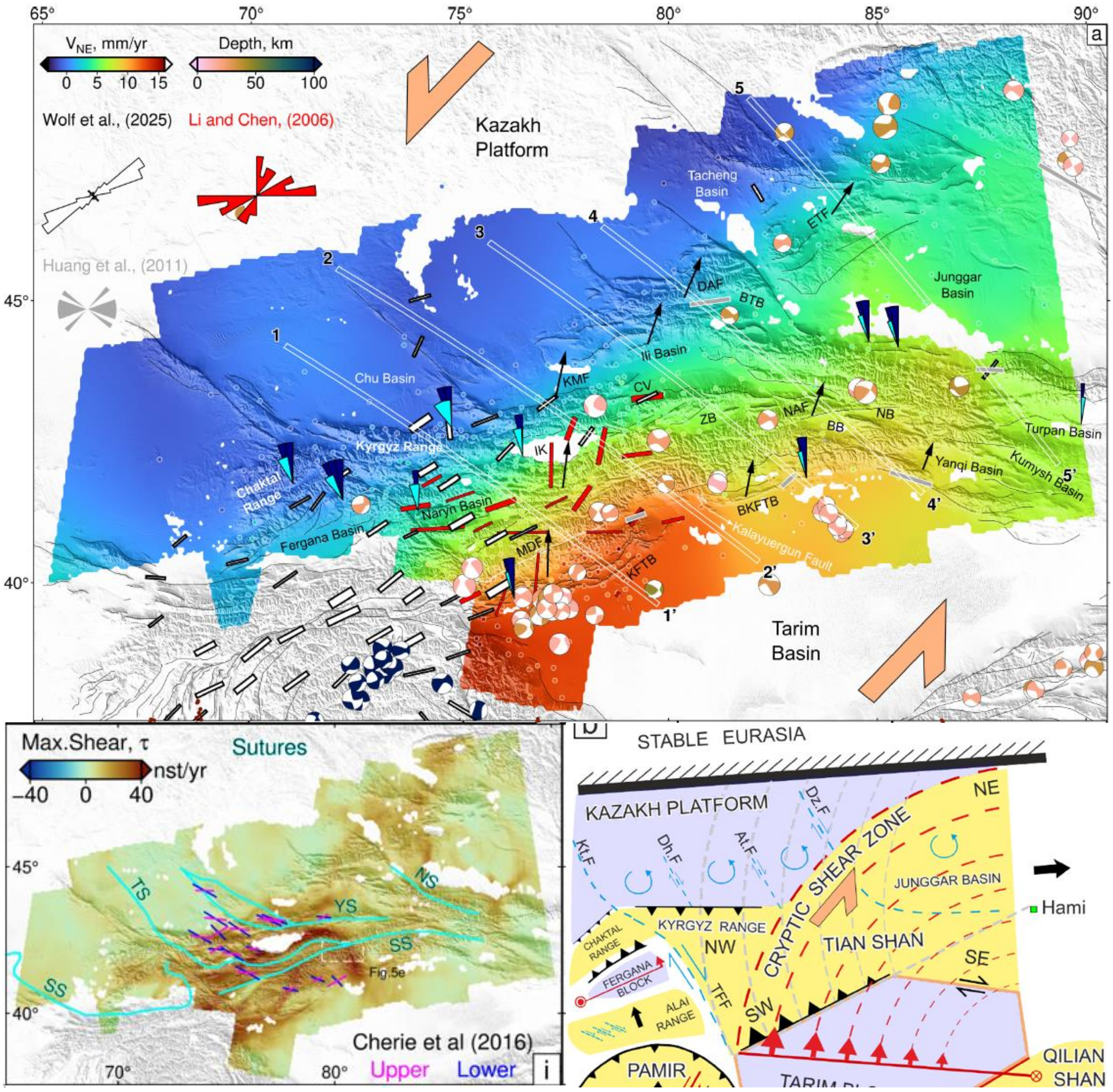

We particularly like a newly published paper (Xie et al., 2026) showing the Paleomagnetic vertical-axes rotation records along the Altyn Fault and through the Qilian Shan, which recorded a shift of tectonic regime at ~12.4 Ma.

H., Liu, C., Zhang, Z., Wang, W., Tamer, M., Zhao, X., Liu, K., Zhang, J., Li, X., Liu, Y., Yu, J., Zheng, D., Wang, Y., Zhang, H., Zhang, P.: Tectonic transition in the northern Tibetan Plateau during the Neogene. Earth and Planetary Science Letters 683, 119997 (2026) https://doi.org/10.1016/j.epsl.2026.119997

The closest evidence we found in Tian Shan is (Line 837):

"a reversal of the paleomagnetic rotations from clockwise to counterclockwise at ~12Ma along the southern edge of the Tian Shan [90]."

Huang, B., Piper, J.D.A., Peng, S., Liu, T., Li, Z., Wang, Q., Zhu, R.: Magnetostratigraphic study of the Kuche Depression, Tarim Basin, and Cenozoic uplift of the Tian Shan Range, Western China. Earth and Planetary Science Letters 251, 346–364 (2006) https://doi.org/10.1016/j.epsl.2006.09.020

Therefore, we encouraged future acquisition of new evidence in Lines 995-1002:
"To further test the nascent nature of the NE-trending shear zone, it would be interesting to see new evidence of reversals of paleomagnetic rotations [90, 121], or initiation / speed-up of anticlockwise rotation along the NE-trending diagonal of the Tian Shan that indicates a transition of tectonic regime around ~10 Ma. Such evidence is currently lacking from the interior of the Tian Shan."

Other geological evidence are related to the timing of accelerated uplift, exhumation and sedimentation, which suggest a change of tectonic regime around 10Ma, which we connect with our new tectonic story for the Tian Shan as inspired by the tectonic evolution of the Alps.

We compare the Tian Shan with the Alps which is also a nascent shear zone being developed from a rotating secondary indentor in lines 798-827:
"We further explore the possibility of the southward shift of Tarim's Euler Pole being the cause of the "nascent" nature of the range-cutting sinistral shear zone through the Tian Shan, and compare this tectonic transition in Tian Shan with that in a similar tectonic regime in the European Alps (Fig. 4c). In the Alps, the NNW-headed farfield indenter of the African Plate is driving the anticlockwise rotation of the Adriaic microplate to create a right-lateral shear zone that is the Periadriatic Line through the Alps [97], mirroring how the NNE-headed India is driving the clockwise rotation of Tarim to create a left-lateral shear zone in Tian Shan. Both Adria and Tarim are promontories of much larger plates driving their motion. Due to irregular geometry and the random position of the secondary indenter relative to the first indenter, the torque generated by their collision could change the Euler Pole of the secondary indenter, hence changing the tectonic regime at its plate boundaries. Adria's Euler Pole was in Spain in the far west, when it first translated northwestward to indent Europe, causing the westward extrusion of the western Alps and the formation of the dextral Periadriatic Line[97]. After wedging the Adriatic toe in the western Alps, the Euler Pole changed to the Po valley to allow further northwest motion by indenting the Adriatic heel into the eastern Alps, driving present-day right-lateral shear around the heel [98], in the same way that Tarim is driving the cryptic shear zone in the Tian Shan. It is therefore plausible that Tarim's Euler Pole was indeed near Hami [14] (Fig. 1b) at the onset of Tian Shan's shortening around 16 Ma[99], facilitating the north-south shortening that increases to the west through a clamping motion [99]. The southward drift of the Euler Pole to the Qilian Shan might be a result of the direct contact between the Indian lithospheric mantle and the western Tarim Basin [100, 101] in the late Miocene (~10 Ma) [102](Fig. 4b). This change of tectonic regime triggered accelerated exhumation throughout the Tian Shan [13, 81, 103–107], and a reversal of the paleomagnetic rotations from clockwise to counterclockwise at ~12Ma along the southern edge of the Tian Shan [90]."

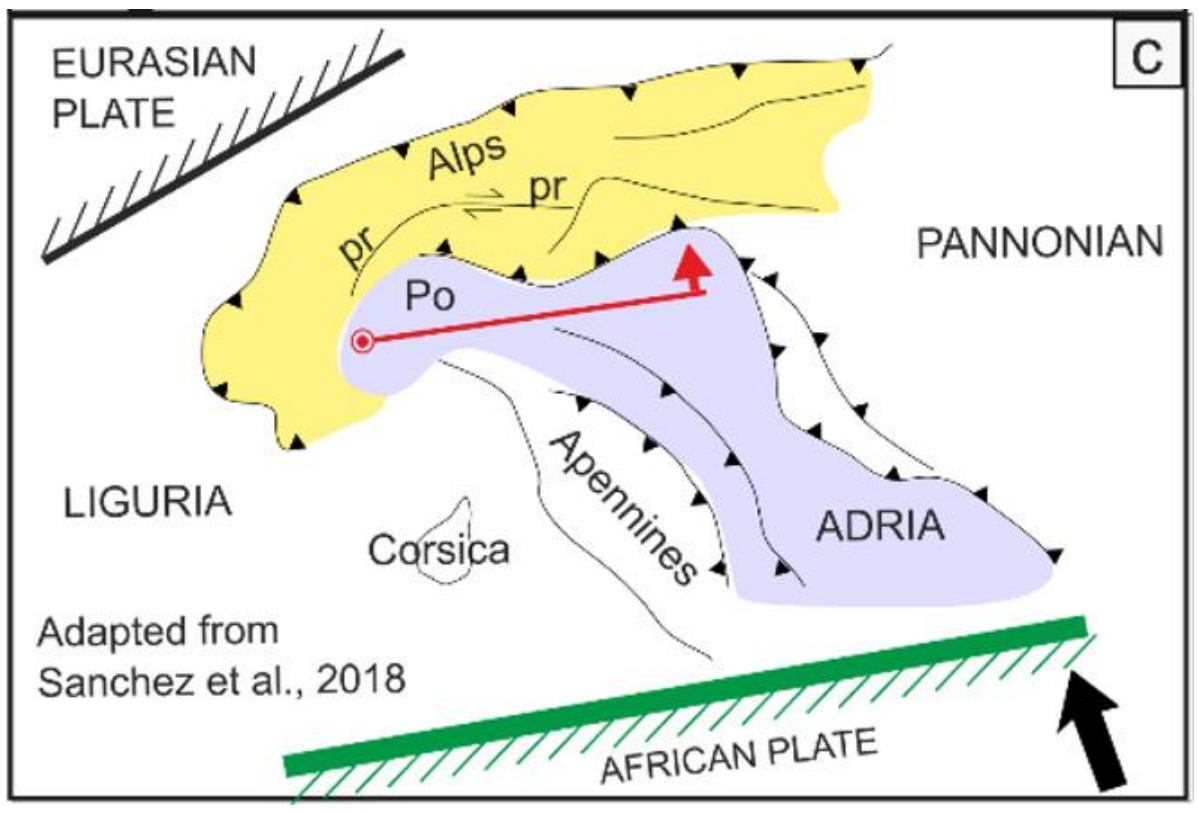

c
EURASIAN
PLATE
Alps
pr
pr
PANNONIAN
Po
LIGURIA
Corsica
Apennines
ADRIA
Adapted from
Sanchez et al., 2018
AFRICAN PLATE

[REDACTED]

Breton, E.L., Handy, M.R., Molli, G., Ustaszewski, K.: Post-20 Ma Motion of the Adriatic Plate: New Constraints From Surrounding Orogens and Implications for Crust-Mantle Decoupling. Tectonics 36, 3135–3154 (2017) https://doi.org/10.1002/2016TC004443
S´anchez, L., V¨olksen, C., Sokolov, A., Arenz, H., Seitz, F.: Present-day surface deformation of the Alpine region inferred from geodetic techniques. Earth System Science Data 10, 1503–1526 (2018) https://doi.org/10.5194/ESSD-10-1503-2018

(2) The manuscript convincingly links the deformation to the rotation of the Tarim Basin around a Euler pole to its southeast. However, the underlying driving forces for this specific rotational kinematics could be discussed in greater depth. A more detailed discussion on how this rotation fits into the broader context of the India-Eurasia collision, potentially relating to the indentation of the Pamir/India or large-scale mantle flow, would provide a more complete dynamical picture.

Thanks for your suggestion. We significantly expanded on the discussion of the driving forces for this rotation kinematics. First we compared the azimuths of our GNSS Data with that predicted from Tarim's rotation around the Qilian Shan to confirm that the current velocity field to the southeast of the NE-trending shear zone is indeed driven by the clockwise rotation of the Tarim Basin.

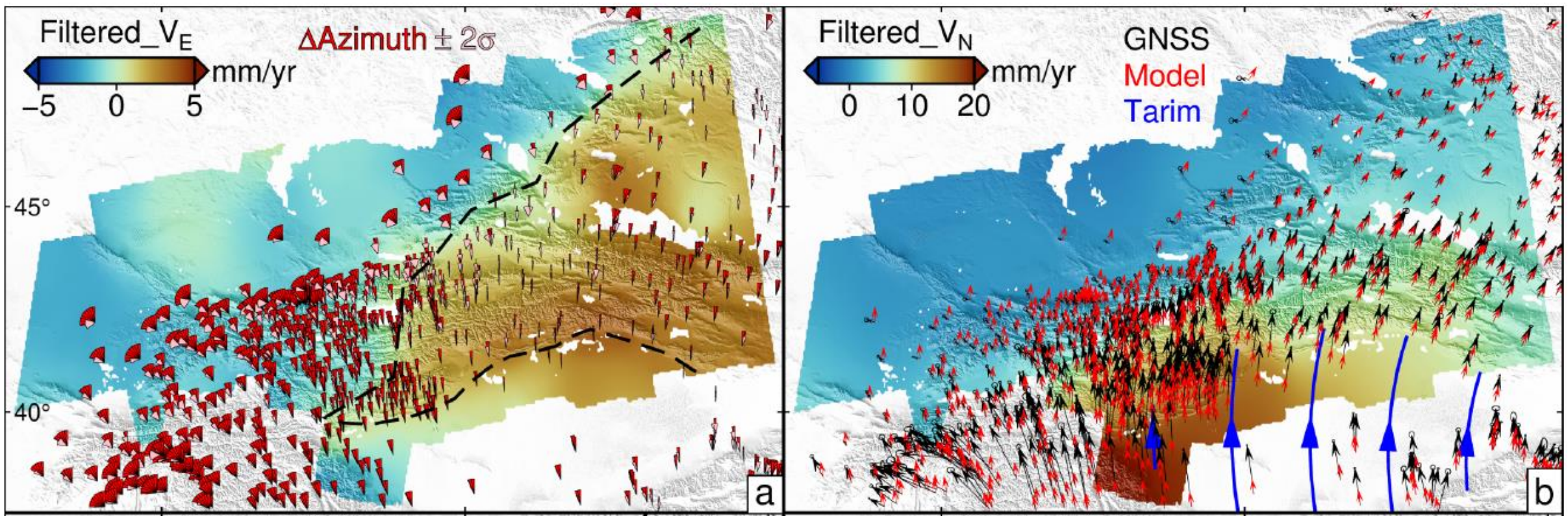

We note this Euler Pole is 500 km south of the tectonically determined Eular Pole near Hami. We propose that there has been a shift of the Euler Pole since the reactivation of the Tian Shan, and drew cartoons (Fig S18) to show that only when the Euler Pole was at Hami, the Kazakh Platform would have felt a northward push that promoted the development and bookshelf rotation between the NW-trending strike-slip faults. The southward shift of the Euler Pole would then explain why the present-day slip rates on these platform-cutting dextral faults are negligible despite their magnificent geomorphology.

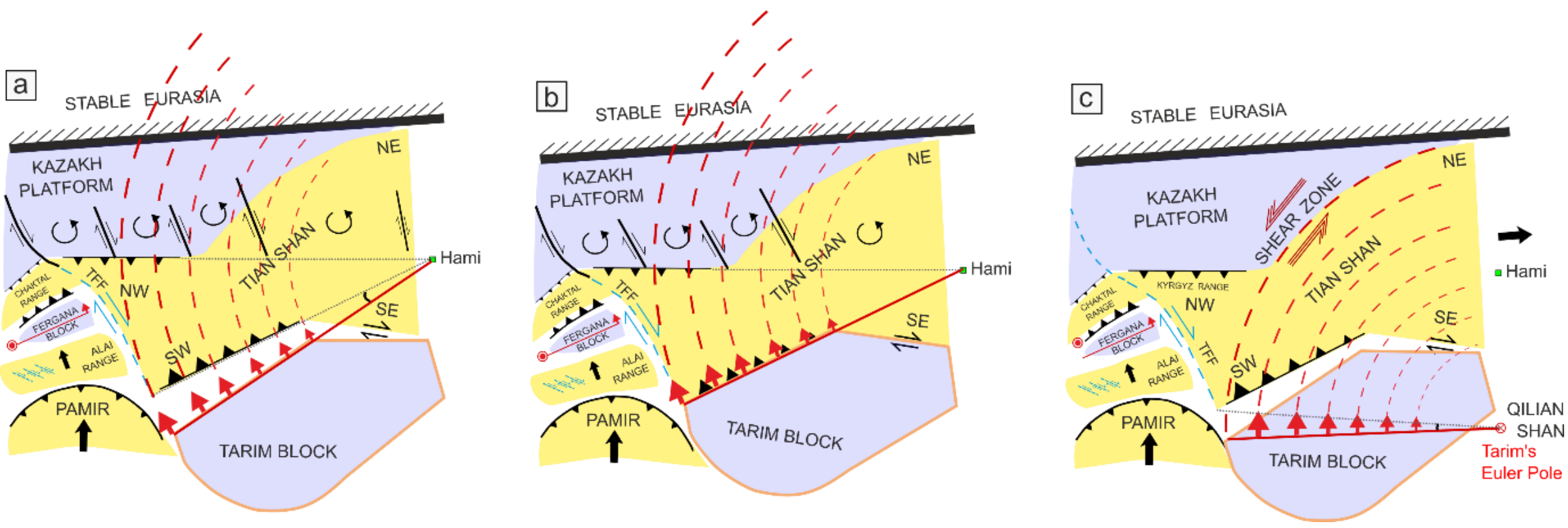

We then compiled various lines of evidence to propose the timing of this shift to be around 10 Ma, due to the contact between Indian and Tarim's lithospheric mantle. Before the Euler Pole shift, the northward indentation of the Pamir Plateau separated the Tarim and Tajik Basin. The translation between Pamir and Tarim drave rapid slip rates on the Talas Fergana Fault and the Karakoram Fault. After Indian and Tarim lithospheric root came into contact, the translation between Pamir and Tarim slowed or stopped, hence the Talas-Fergana Fault and the Karakoram Fault became less active, Tarim's Euler Pole shifted southward, the rotational stress of the Tarim no longer cause northward compression in the Kazakh Platform hence the parallel NW-trending strike-slip faults also became inactive. We described this thinking in the newly added result subsection titled "Shifted Euler Pole from Indian Tarim Collision?" (Lines 796-926).

(3) The proposed "hierarchical wedge model" is interesting, but its advantages and limitations could be more explicitly contrasted with existing models (e.g., the "bookshelf model"). Please more clearly state which specific observations the new model explains better than previous ones. In addition, a brief discussion on the model's limitations would strengthen the discussion.

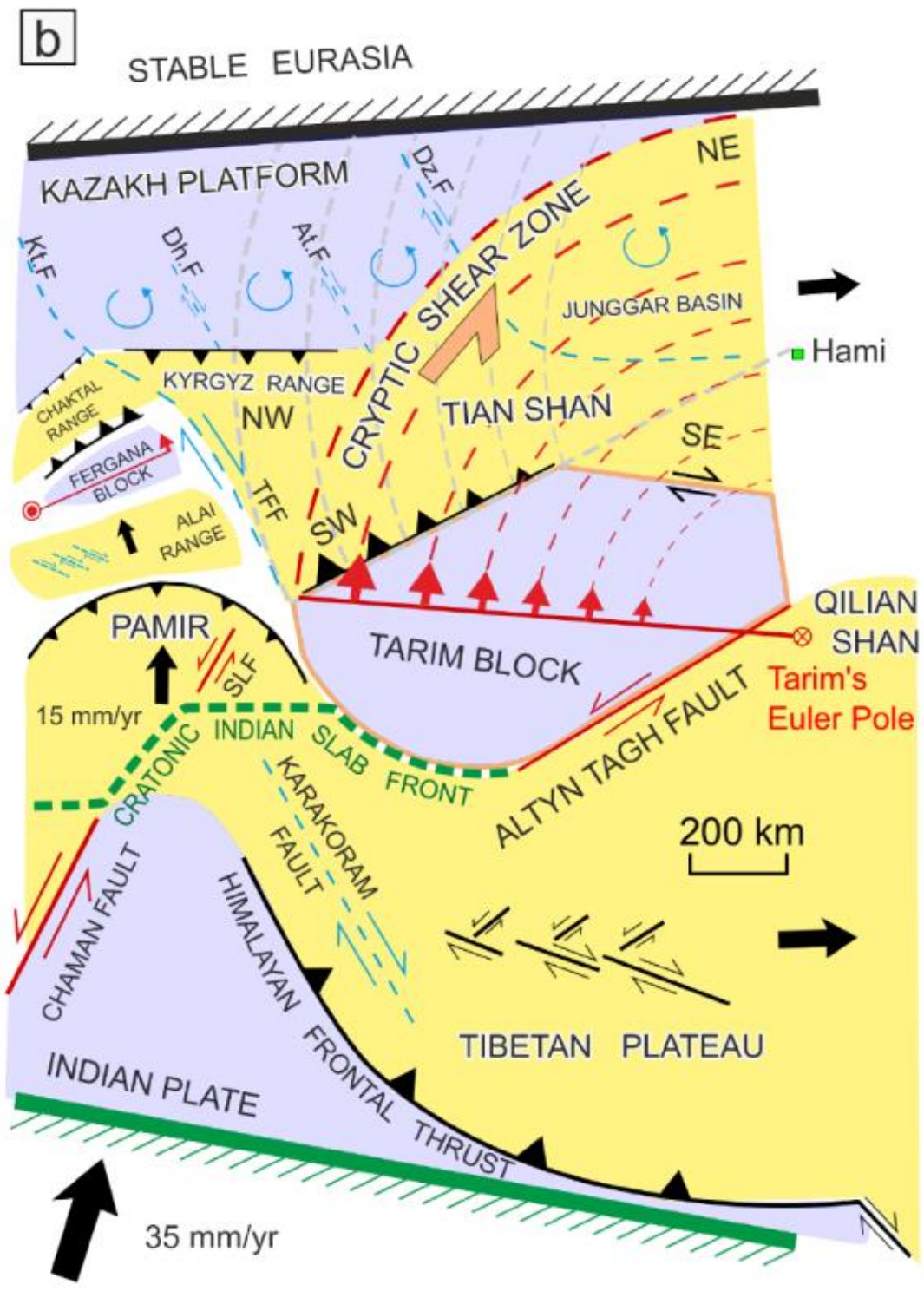

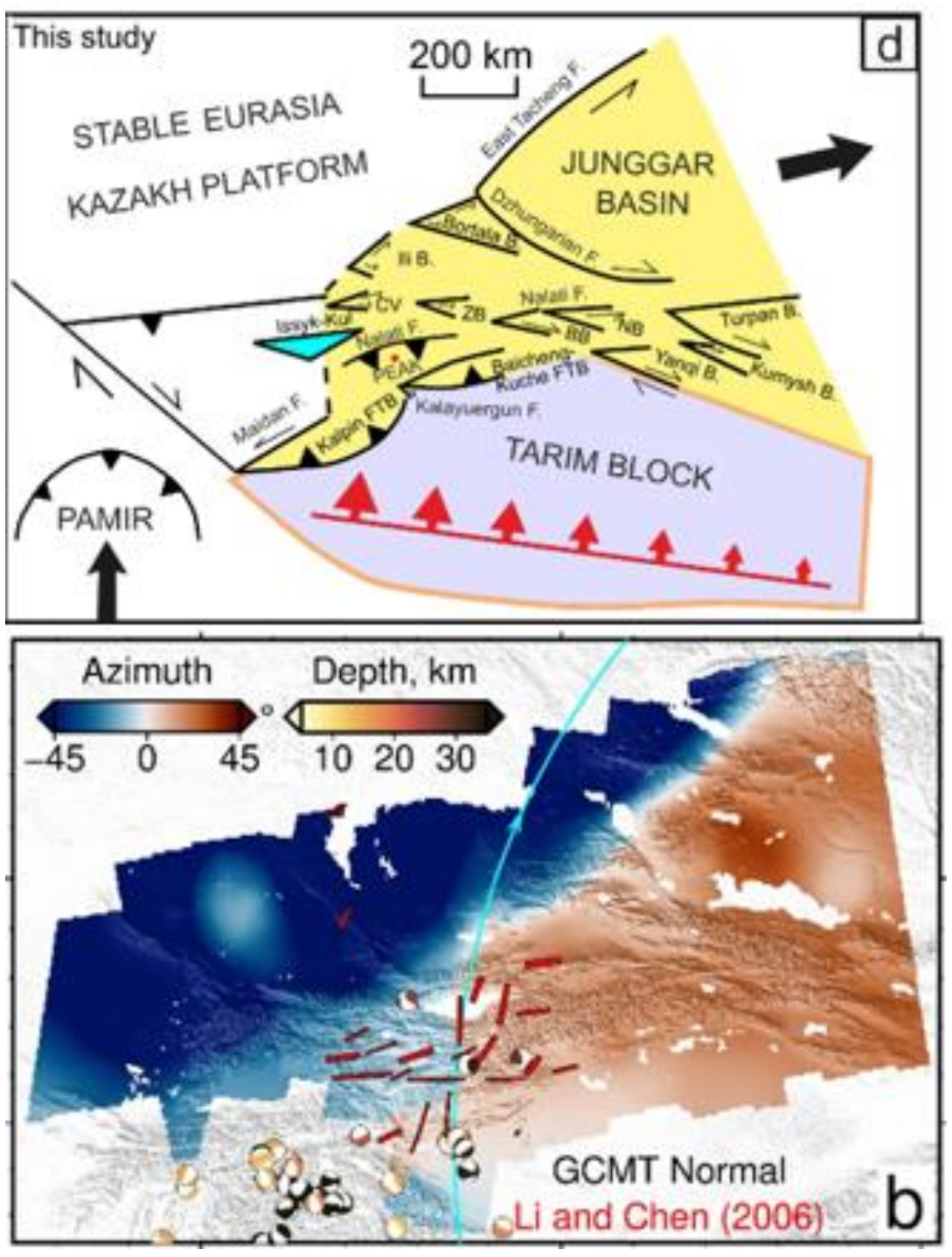

Thanks for your suggestion. Following the construction of a more complete dynamic picture, we now think that the two models describe two styles of deformation that were/are active in the Tian Shan at different locations due to contrasting rheological properties of the deforming materials and at different times due to the change of stress field. We expanded on the contrast between the two models and included a discussion of the model's limitation in the following paragraph (Lines 1031-1069):

"The triangular wedge model contrasts with the bookshelf model traditionally employed to describe the parallel dextral faulting in the Kazakh Platform (Fig. 1c). The bookshelf model predicts the strike-slip motion on parallel new faults generated in a pristine and rigid material under a compressional stress governed by Anderson's theory of faulting [127]. Our hierarchical wedge model predicts the sense of strike-slip component motions on pre-existing faults in response to a changing stress field.

Our model does not predict slip rates or the sense of dip-slip component motion on the faults. The bookshelf rotation describes well the platform-cutting NW-striking dextral faults and was likely the dominant lengthening mechanism when Tarim had a Euler Pole near Hami and exerted a northward compressional stress through the Kazakh Platform. After the southward shift of Tarim's Euler Pole, the stress-field bypassed the Kazakh Platform (Fig. 4b). The eastward crustal escape through the conjugate component strike-slip motions along the various-sized triangular wedges within the southeastern Tian Shan became a more dominant lengthening mechanism. The dif- ferent styles of tectonic response come from the different rheologies characterising the northwest and southeast halves of the Tian Shan orogeny. To the north of the NE- trending shear zone, the Tian Shan is pushing against the Kazakh Platform where the crystalline basement [68, 128, 129] promotes brittle faulting along parallel strike-slip faults [130, 131]. To the southeast of the shear zone, except the Junggar Basin, which acts mostly as a rigid block [79], the weaker Tian Shan material [132] can deform more continuously, distributing itself to the east where there is no hard boundary [38]. Therefore, our observation and conceptual model are also consistent with the dynamic models that suggest how stress orientation and rheological structures together control the deformation fields [95]."

(4) The identification of non-tectonic signals (permafrost creep, glacial isostatic adjustment, groundwater extraction) is a strength. However, the methodology for quantitatively separating these signals from the tectonic background is not fully detailed.

Thank you for your request. We discuss potential future direction for quantitatively separating these signals in Lines 1099-1101:

"These signals are kilometre-scaled and highly correlated with slope aspects, making them uniquely identifiable (e.g., through high-pass filtering) without obscuring larger-scale tectonic interpretations."

And in Lines 1138-1153:

"The fast thrust motion could potentially result from climatic-tectonic interplay where the glacial isostatic adjustment (GIA) causes a buoyancy that promotes slip on the fault [141–143]; in this case, creep on the fault because of the suture zone. To our knowledge, no such case has been reported or modelled before. Characterising this interplay might require coupling a finite-element GIA model with a tectonic stress field with faults [144] with finite-element rate-and-state modelling with a velocity-strengthening suture zone [145], which is likely implementable through the Pylith package [146, 147]."

(5) For the Introduction section, although it effectively sets the scene but could be more explicitly state the key knowledge gap and the specific contribution of this study in the final paragraph.

Thank you for your suggestion. We have expanded the last paragraph of the introduction section to introduce a range of new thinking that we derived from our analysis.

"In this study, we process 100,000 interferograms using Sentinel-1 data and compile 673 GNSS velocities from 22 published studies to derive the first 500m resolution east and vertical velocity fields (Fig. 1d,e) and 5 km-resolution vorticity and strain rate fields (Fig. 2) covering the majority (over 1.6 million km2) of the Tian Shan mountain belt (Section 4, Text S1–S7, Fig. S1–S17). The east velocity gradients and vorticity fields (Fig. 2c,e,g) reveal a newly identified range-scale and range-cutting arcuate left-lateral shear zone trending northeast. Based on our velocity, vorticity and strain rate mapping, and from earthquake focal mechanisms (Fig. 2d,f,h) and previously published shear wave splitting (Fig. 2e,g,i,j) and paleomagnetic rotation measurements (Fig. 3a), we explore the possibility that the Tian Shan is in the early stage of developing a range-cutting shear zone centered along a NE-trending diagonal (Fig. 2a,b, 3, 4). We show that this newly identified shear zone is driven by the

clockwise rotation of the Tarim Basin, and propose that its nascent nature is due to a southward shift of the Tarim's Euler Pole following the Indian-Tarim lithospheric contact which changed the stress field and slowed down the platform-cutting strike-slip faults, such as the prominent Talas Fergana Fault. We further propose a hierarchical triangular wedge model to explain the sense of the strike-slip component motions on individual basin-bounding faults across the southeastern half of the Tian Shan. These conjugate strike-slip component motions across different spatial scales collectively play a more important role in accommodating the shortening and extension of the Tian Shan in this sinistral range-scale shear zone than the dextral slip on the platformcutting strike-slip faults. By examining our vertical component of the derived surface velocity (Fig. 5), we show how the rates, spatial scales, and mechanisms of uplift are heterogeneous across the mountain belt, with glacial isostatic adjustment potentially directing the localisation of shortening and uplift near the highest peak in southern Tian Shan (Jengish Chokusu, Tomur Feng, or Victory Peak, 7,439 m, Fig. 1e).

Reviewer #3 (Remarks to the Author):

This paper presents a comprehensive and high-resolution analysis of crustal deformation across the Tian Shan orogenic belt, combining an extensive dataset of Sentinel-1 InSAR interferograms. The study makes several key contributions: 1) High-resolution deformation maps: the authors derive the east-west and vertical velocity fields, and strain-rate fields covering over 2 million km² of the Tian Shan. This represents a significant advancement in spatial resolution and coverage compared to previous studies. 2) Based on large-scale Earth observations from space, this paper proposes a novel explanation: a NE-trending, range-cutting shear zone driven by the rotation of the Tarim Basin. 3) The authors propose a kinematic model involving east-opening triangular wedges at multiple scales to explain the eastward crustal flow and extensional deformation in the south-eastern Tian Shan. This model offers a fresh perspective on how deformation is accommodated in the region.

The InSAR big-data processing is supported by the Looking into Continent from Space (LiCS) system of the COMET team. The data-processing strategy is robust, and I believe the results are solid. The article's logic is clear and its structure is sound.

Thank you for your review of our manuscript and for your recognition of our data processing strategy and the new perspective on the deformation model of the Tian Shan. We have improved the manuscript based on your suggestions.

Minor Suggestions for Revision:

1) While the velocity gradients, vorticity and strain-rate fields strongly suggest the presence of a shear zone, the authors should more explicitly discuss the limitations or alternative interpretations of this feature. For example, could the signal be influenced by deeper crustal flow without a clear surface expression?

Thank you for your question. Your suggestion of a deeper crustal flow without a clear surface expression is in line with our thinking. We believe that the ductile lower crust and the upper mantle underneath the part of the Tian Shan southeast of the NE-trending shear zone are indeed flowing eastward due to being driven by the rigid and rotating Tarim Basin (Fig 4a).

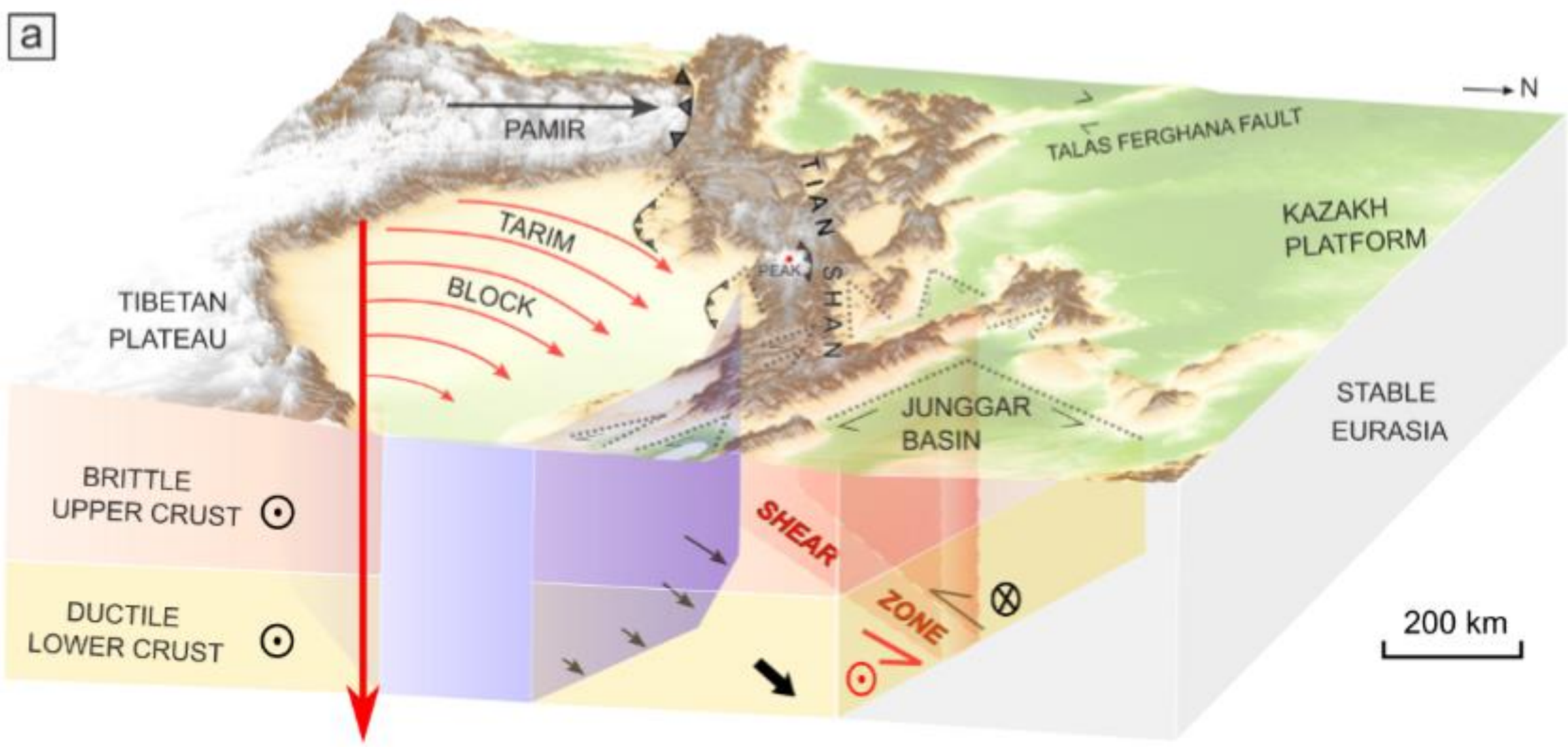

We add to the previous interpretation that we now think that this nascent shear zone is created because of a southward shift of the Tarim's Euler Pole, which changed the stress field from northward-directed to more northeastward direction. As the Tian Shan has a long and complex tectonic history with many preexisting faults already optimally oriented under the old stress field, the upper crustal faults are adjusting to accommodate the deformation driven by the new stress field, hence the less clear surface expression. We have added a new result section with the heading "2.3 Shifted Euler Pole from Indian-Tarim Collision?" to explain the potential cause of stress field change, and hence the nascent nature of the cryptic shear zone. We suggest that the most likely timing of this Euler Pole shift is around 10 Ma, in line with the timing of the Indian-Tarim collision supported by various independent evidence (Fig 4b).

"2.3 Shifted Euler Pole from Indian-Tarim Collision?

We further explore the possibility of the southward shift of Tarim's Euler Pole being the cause of the "nascent" nature of the range-cutting sinistral shear zone through the Tian Shan, and compare this tectonic transition in Tian Shan with that in a similar tectonic regime in the European Alps (Fig. 4c). In the Alps, the NNW-headed farfield indenter of the African Plate is driving the anticlockwise rotation of the Adriaic microplate to create a right-lateral shear zone that is the Periadriatic Line through the Alps [97], mirroring how the NNE-headed India is driving the clockwise rotation of Tarim to create a left-lateral shear zone in Tian Shan. Both Adria and Tarim are promontories of much larger plates driving their motion. Due to irregular geometry and the random position of the secondary indenter relative to the first indenter, the torque generated by their collision could change the Euler Pole of the secondary indenter, hence changing the tectonic regime at its plate boundaries. Adria's Euler Pole was in Spain in the far west, when it first translated northwestward to indent Europe, causing the westward extrusion of the western Alps and the formation of the dextral Periadriatic Line[97]. After wedging the Adriatic toe in the western Alps, the Euler Pole changed to the Po valley to allow further northwest motion by indenting the Adriatic heel into the eastern Alps, driving present-day right-lateral shear around the heel [98], in the same way that Tarim is driving the cryptic shear zone in the Tian Shan. It is therefore plausible that Tarim's Euler Pole was indeed near Hami [14] (Fig. 1b) at the onset of Tian Shan's shortening around 16 Ma[99], facilitating the north-south shortening that increases to the west through a clamping motion [99]. The southward drift of the Euler Pole to the Qilian Shan might be a result of the direct contact between the Indian lithospheric mantle and the western Tarim Basin [100, 101] in the late Miocene (~10 Ma) [102](Fig. 4b). This change of tectonic regime triggered accelerated exhumation throughout the Tian Shan [13, 81, 103–107], and a reversal of the paleomagnetic rotations from clockwise to counterclockwise at ~12Ma along the southern edge of the Tian Shan [90].

The direct contact between the Indian and Tarim lithospheric mantle could also explain the slowing down of the right-lateral slip rates on the Talas-Fergana and Karakoram Faults (Fig. 4b). The prominent right-lateral Talas-Fergana fault bounding the western Tian Shan, which had higher geological slip rates of 2.2–16 mm/yr [19, 20, 23], is currently geodetically moving at a much slower rate of ~1.5 mm/yr [16]. In our results, the change in east component of velocity across the Talas-Fergana Fault is ~2 mm/yr, which translates to ~2.3 mm/yr of slip rate distributed over a 200km zone (Fig. 2a,f), playing a less important role in accommodating shortening and lengthening than the NE-directed nascent shear zone identified in this study with over 10 mm/yr of velocity change distributed over 400 km of mountain range (Fig. 3a). Similarly, the Karakorum fault of the Western Tibetan Plateau, which had geological slip rates of 4–8 mm/yr [108–110], is geodetically inactive in the north and slowly slipping at 1.6–3.2 mm/yr in the south [111]. Cenozoic strike-slip motion on both the Talas-Fergana and Karakoram Faults commenced ~25 Ma, synchronous with the onset of

exhumation and dextral strike-slip faulting along the eastern flank of Pamir and western Tarim [112, 113]. These right-lateral slips once played an important role in accommodating the Pamir's northward indentation that separated the Tarim and Tajik Basin [114] (Fig. 1b). That was when magmatism clustered in southern Tibet due to the delamination of the Tibetan lithospheric mantle, before the cold Indian lithospheric mantle shut off the heat from the asthenosphere at ~10Ma [101, 102]. As the Indian lithospheric mantle came into direct contact with the rigid deep lithospheric root of Tarim, like how the Indian and Asian lithospheric mantle are locking underneath Pamir today [54, 86], Pamir and the Western Tarim effectively became a united collisional front. They move north at similar rates, slowing the right-lateral strike-slip motion on the once-highly-active Talas-Fergana and Karakoram Faults. Hence, very limited strike-slip motion was recorded between Pamir and the Tarim since ~11Ma [115].

The roles of the NW-striking platform-cutting dextral faults (KtF, DhF, AkF, DzF,and ChF in Fig. 1b,c) likely also diminished with the changing stress field due to the rotation of the Tarim Basin. It was estimated that the Tarim Basin has rotated clockwise by 7±2.5◦ since the late Cenozoic, assuming an Euler Pole near Hami [14, 99] (Fig. 1b and 4b). If the Euler Pole were indeed around Hami near the onset of Tian Shan's reactivation, the stress field felt by the Kazakh Platform would have been northward compression (Fig. S19a,b), which promotes the development of the parallel Nw-trending platform-cutting strike-slip faults. These dextral faults likely formed trending ~N30◦E [116] and accommodated shortening by anticlockwise block rotation [22, 23, 117], hence rotating themselves to be NW-trending. We do not think Tarim's Euler Pole has always been in the Qilian Shan, as if so, the platform-cutting dextral faults could not have developed into the magnificent structures they are, and the nascent shear zone that we describe would have had more time to mature (Fig. S19c). Hence, it is likely that the southward shift of Tarim's Euler Pole to the Qilian Shan, due to the India-Tarim lithospheric contact around 10 Ma[68, 100, 101](Fig. 4b), rotated the stress field to point northeasterly (Fig.3a). This new stress field bypasses the platform-cutting dextral faults. Hence the present-day strain rate on these structures becomes negligible (Fig. 1d and 2a,c). The Talas-Fergana Fault is still active [23] because of the differential north-south shortening rates in the Tian Shan that still exist north of Pamir and Tarim, respectively [5]. The Dzhungarian Fault is still active [22, 117] due to its new role in facilitating the eastward translation of the Junggar Basin (Section 2.4)."

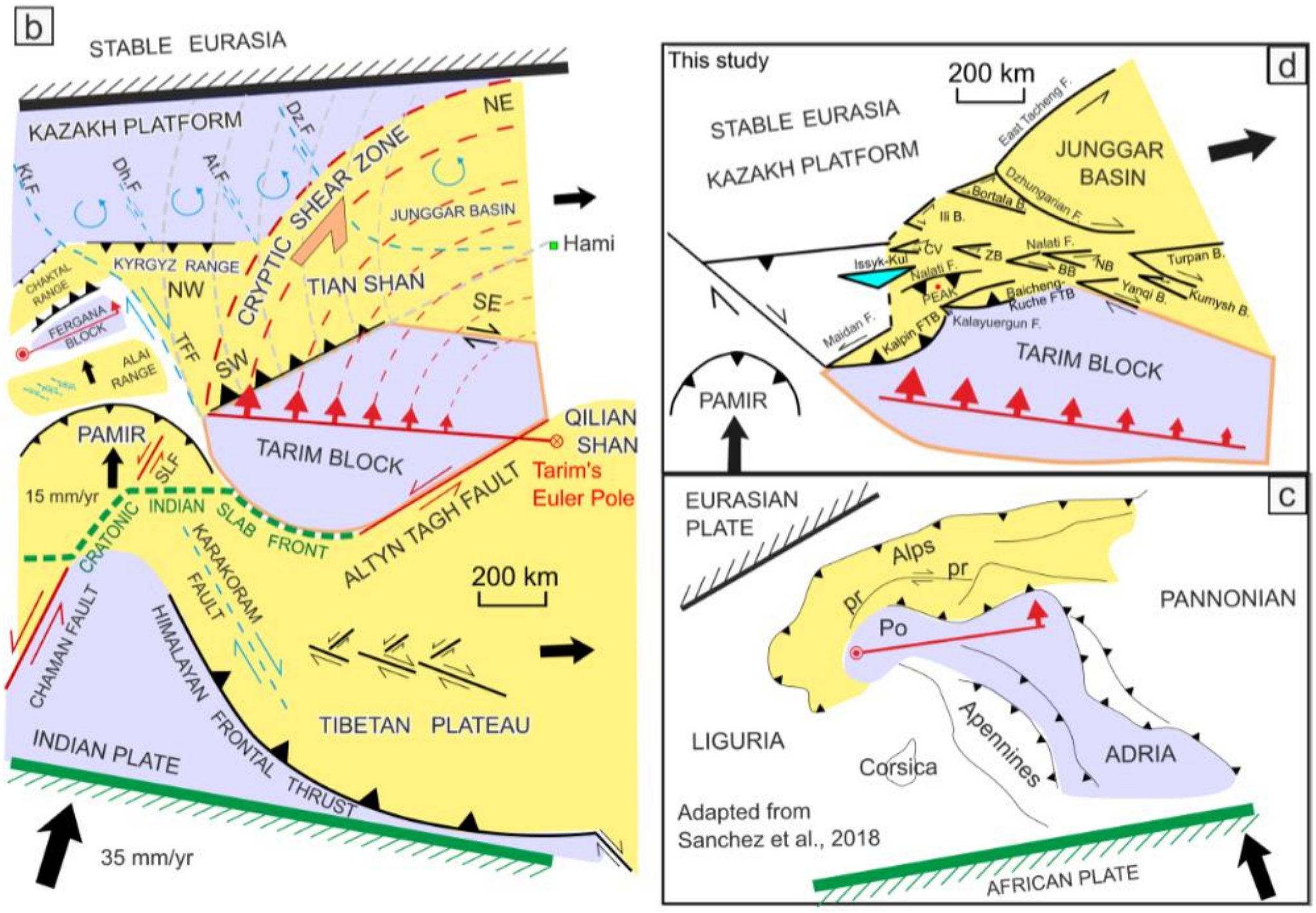

However, we did not find in the literature evidence of the timing of initiation or reversal of paleomagnetic vertical-axes rotation along the shear zone, as all available paleomagnetic rotation data reported were around the edges of the Tian Shan and were interpreted to be representing local rotation. Nevertheless, this is in line with our proposed hierarchical triangular wedge model (Fig 4d) where the basin bounding faults of each triangular wedge form conjugate strike-slip pairs to accommodate the eastward crustal flow, hence inducing local rotations. We discuss this limitation in Section 2.4 Lines 975-999:

"This hierarchical wedge model, by allowing the small wedges to respond to the stress field in their own way, makes it possible to explain the counterclockwise verticalaxis rotations reported along the southern edges of the Tian Shan and of the Junggar Basin (Fig.3a). Both Huang et al. [90] and Charreau et al. [62] believe that these fast anticlockwise rotations represent localised small-block rotation. Indeed, if the entire southeastern half of the Tian Shan were to systematically rotate like a rigid block, the paleomagnetic records across the wedge would all show clockwise rotation like Tarim, for which we found no evidence in the literature. The localised rotations and fault slips likely represent the optimal way through which the southeastern Tian Shan responds to the new stress field, exploiting pre-existing structures that happen to be conjugate to each other. To further test the nascent nature of the NE-trending shear zone, it would be interesting to see new evidence of reversals of paleomagnetic rotations [90, 121], or initiation / speed-up of anticlockwise rotation along the NE-trending diagonal of the Tian Shan that indicates a transition of tectonic regime around ~10 Ma. Such evidence is currently lacking from the interior of the Tian Shan."

However, we do not think there is current deeper crustal flow along the NW-SE axes as suggested by Cherie et al., 2016, based on their two-layer shear wave splitting results that show that there is fabric oriented NW-SE in the bottom layer. This orientation is not compatible with the stress field revealed by our velocity fields. We suggest that this fabric is inherited from past tectonic events, most likely from the Paleozoic collisions that created the now NW-SE oriented suture zones (Fig 2i). We explain our reasoning in Lines 635-650:

"However, our vorticity and strain rate patterns are at odds with the NW- and NNW-oriented fast axes derived from a two-layer model employed by Cherie et al. [57]. In particular, the NNW-directed lower layer anisotropy, interpreted as the NNW-directed mantle flow driven by the Tarim subduction in Cherie et al. [57], will not be compatible with our velocity field, unless the ductile portion of the lithosphere and the mantle are flowing almost perpendicular to each other; more likely, the orientation of the lower-layer anisotropy represents frozen fabric between the NW-trending suture zones generated during the Paleozoic closure of oceanic basins (Fig. 2i), rather than current flow direction."

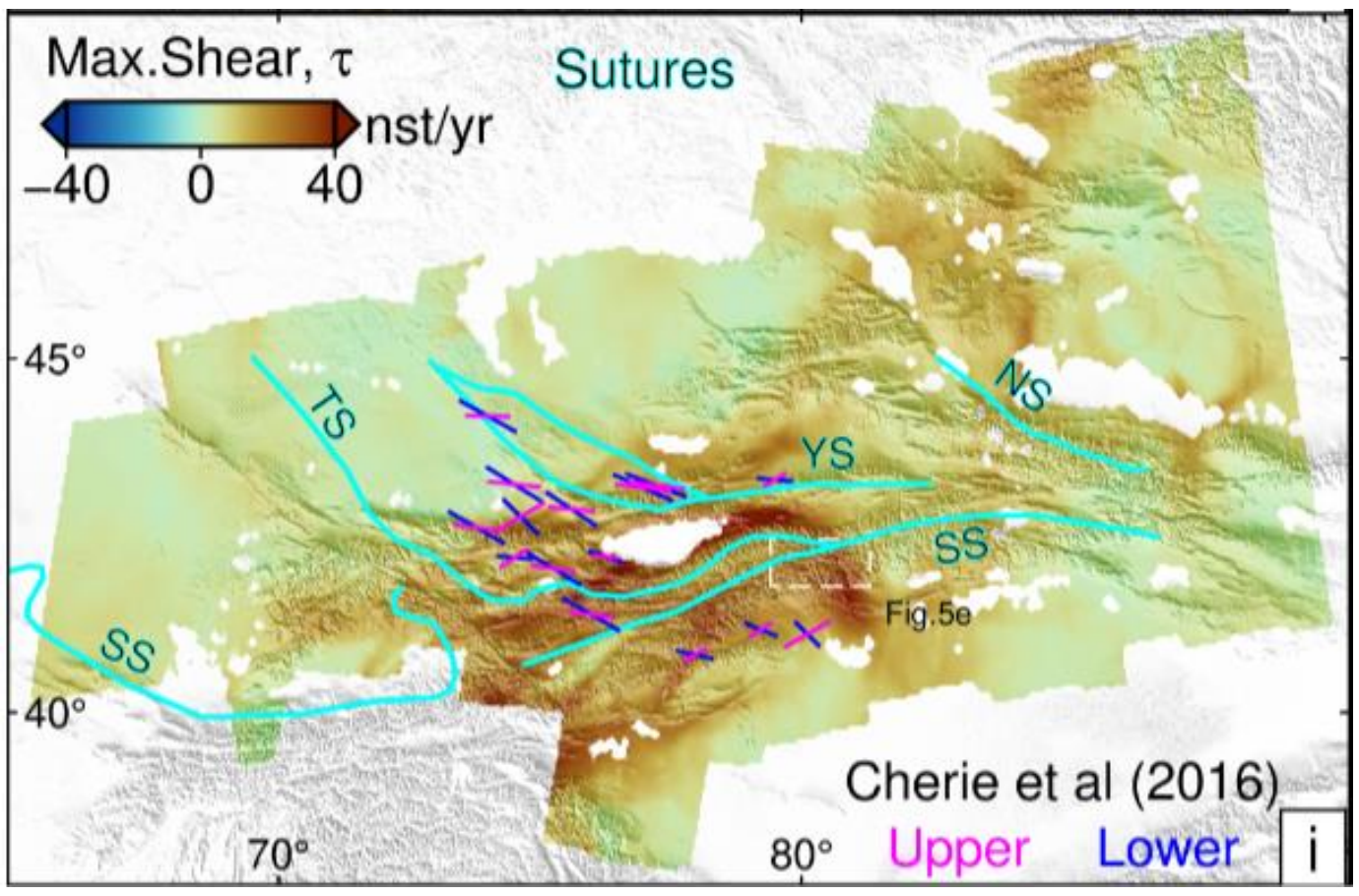

2) The InSAR velocity results are sensitive to errors such as atmospheric delay, especially for wide-area processing. A brief description of how these were mitigated, and an estimate of the remaining uncertainties, would strengthen the interpretation of vertical motions.

Thank you for your suggestion. We have added our approach to atmospheric correction in Lines 1402-1403: "Tropospheric delay was corrected through using the Generic Atmospheric Correction Online Service (GACOS) [163–165]."

In particular, we expand on how we used GACOS to facilitate unwrapping in the supporting information Text S1.

"(3) For phase unwrapping, we used the Statistical-cost, Network-flow Algorithm for Phase Unwrapping (SNAPHU) (Chen and Zebker, 2001, 2002) to perform the core phase unwrapping procedure in a processing chain incorporating modified Goldstein filtering (Goldstein and Werner, 1998). To facilitate unwrapping, we combined this step with atmospheric correction and removed atmospheric phase delays given by Generic Atmospheric Correction Online Service (GACOS) (Yu et al., 2017, 2018a, 2018b) before unwrapping, if it reduces the standard deviation of phases in the original interferogram (Lazecky et al., 2022). We retained the connected components by-products of the SNAPHU phase unwrapping to facilitate reference window selection (Text S3) and unwrapping mistake correction (Text S4) after preliminary time series analysis."

We have also added a full uncertainty analysis in the methodology section (Lines 1505-1537) and added two new figures in the supporting information:

"We estimate the uncertainties of our line-of-sight and decomposed east and vertical velocities exploiting redundancies at track boundaries [87, 191](Fig. S16). The uncertainties of east and vertical velocities decomposed from one ascending and one descending tracks are 2.0 mm/yr and 1.6 mm/yr, respectively, estimated at diamond shapes overlapped by two ascending and two descending tracks. Propagating these numbers, and a conservative uncertainty of 3 mm/yr for north velocities, through the unit vector of the average line-of-sight yields an uncertainty of 1.8mm/yr for line-of-sight velocities predicted from the east and vertical velocities. At stripes overlapped by three tracks (2 ascending + 1 descending, or 1 ascending + 2 descending), the observed line-of-sight velocities, and those predicted from the east and vertical velocities decomposed from the other two independent ascending and descending tracks, have root-mean-squared differences of 2.6 mm/yr for ascending overlaps and 2.2 mm/yr for descending overlaps. Removing the 1.8 mm/yr of uncertainties of the predicted line-of-sight velocities from these differences yields 1.9 mm/yr and 1.2 mm/yr of uncertainties for observed ascending and descending line-of-sight velocities. Propagating these two line-of-sight velocity uncertainties through the velocity decomposition gives uncertainties of 1.6±0.2 mm/yr and 1.3±0.2 mm/yr for our full east and vertical velocity fields, which compare well with the 2.1mm/yr and 2.5 mm/yr of RMS differences between InSAR and GNSS east and vertical velocities, respectively (Fig. S17)."

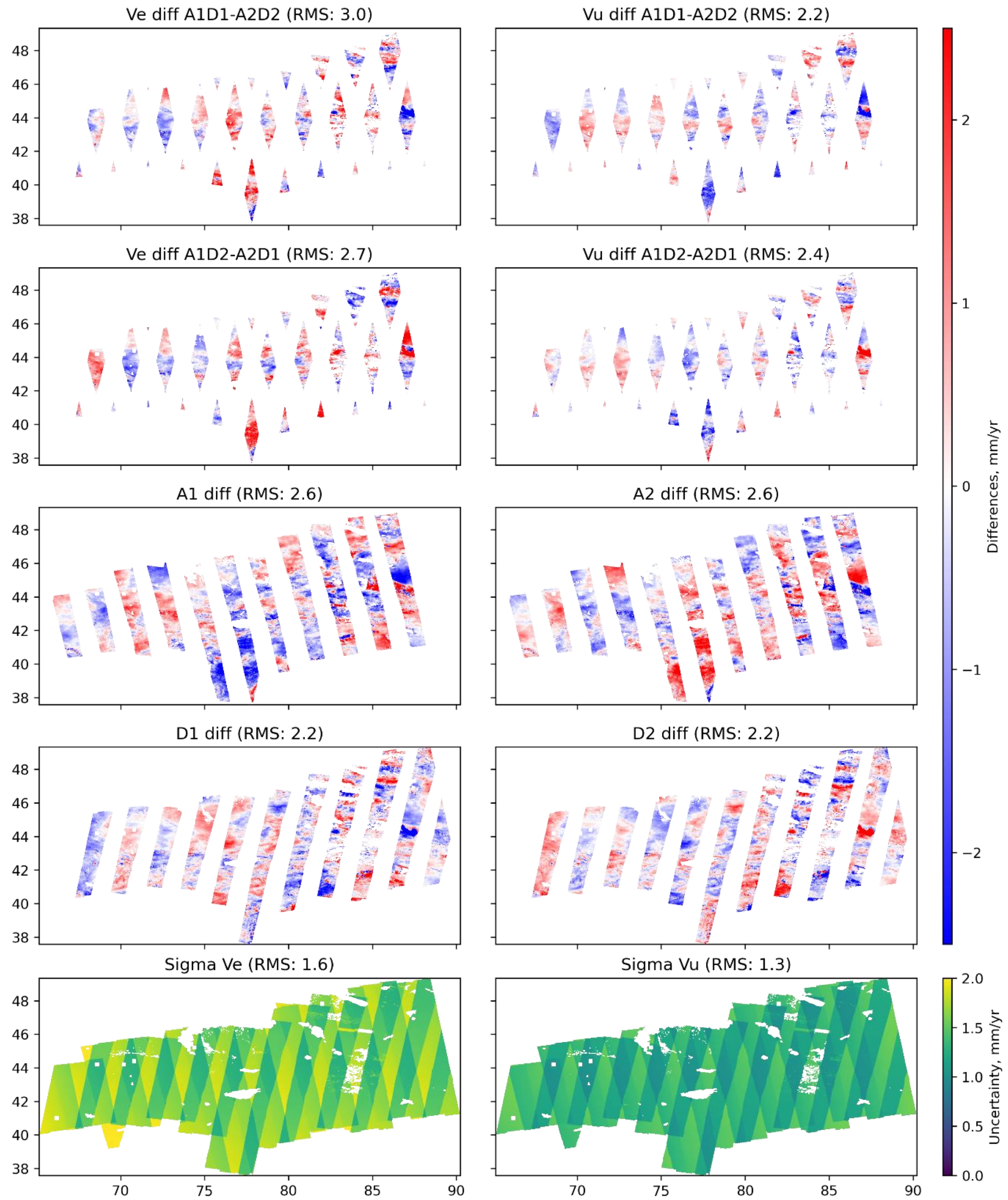

Figure S16: Uncertainty estimations through the evaluation of (top 4 panels) differences between east and vertical velocities decomposed from two independent sets of ascending and descending line-of-sight velocities at the diamond shapes overlapped by 4 tracks, and (middle 4 panels) the differences between observed line-of-sight velocities (in Figure S15) and line-of-sight velocities predicted from east and vertical velocities decomposed from independent ascending and descending line-of-sight velocities at stripes overlapped by 3 tracks. These RMS differences yield 1.9 mm/yr and 1.2 mm/yr as average uncertainties of observed ascending and descending velocities. Propagating them through velocity decomposition gives pixel-wise east and vertical velocity uncertainties (bottom two panels).

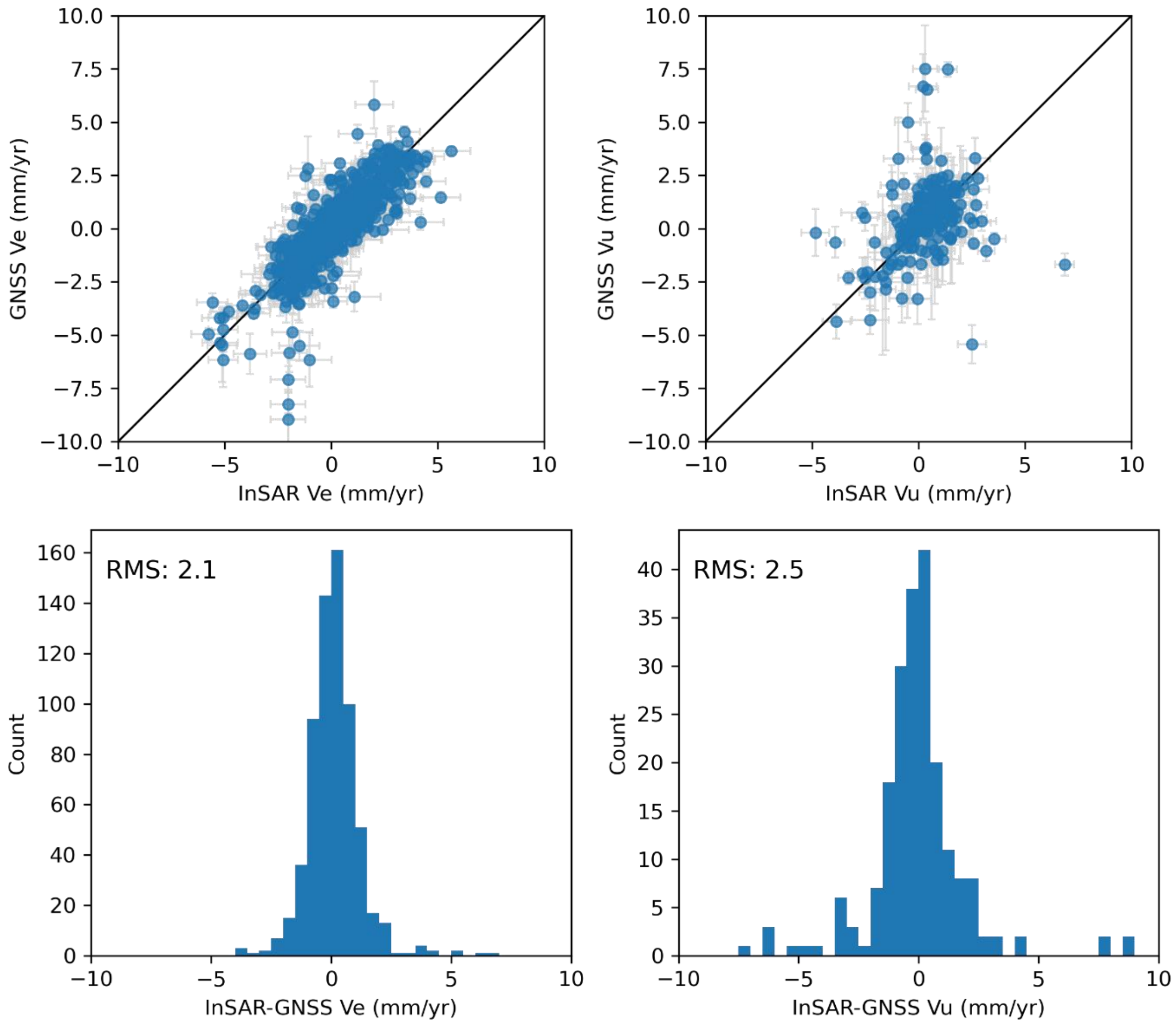

Figure S17: Comparison between InSAR and GNSS east and vertical velocities.

3) The Hierarchical Wedge Model is innovative but could benefit from a more detailed comparison with existing models (e.g., bookshelf faulting) to better highlight its advantages and limitations.

Thanks for your suggestion. We have expanded on the contrast between the two models and included a discussion of the model's limitation in Lines 1033-1069:

"The triangular wedge model contrasts with the bookshelf model traditionally employed to describe the parallel dextral faulting in the Kazakh Platform (Fig. 1c). The bookshelf model predicts the strike-slip motion on parallel new faults generated in a pristine and rigid material under a compressional stress governed by Anderson's theory of faulting [127]. Our hierarchical wedge model predicts the sense of strike-slip component motions on pre-existing faults in response to a changing stress field. This model does not predict slip rates or the sense of dip-slip component motion on the faults. The bookshelf rotation was likely the dominant lengthening mechanism when Tarim had an Euler Pole near Hami and exerted a northward compressional stress through the Kazakh Platform. After the southward shift of Tarim's Euler Pole, the eastward crustal escape through the conjugate component strike-slip motions along the various-sized triangular wedges within the southeastern Tian Shan became a more dominant lengthening mechanism. The different styles of tectonic response come

from the different rheologies characterising the northwest and southeast halves of the Tian Shan orogeny. To the north of the NE-trending shear zone, the Tian Shan is pushing against the Kazakh Platform where the crystalline basement [68, 128, 129] promotes brittle faulting along parallel strike-slip faults [130, 131]. To the southeast of the shear zone, except the Junggar Basin, which acts mostly as a rigid block [79], the weaker Tian Shan material [132] can deform more continuously, distributing itself to the east where there is no hard boundary [38]. Therefore, our observation and conceptual model are also consistent with the dynamic models that suggest how stress orientation and rheological structures together control the deformation fields [95].”

Reviewer #3 (Remarks on code availability):

LiCSBAS is a time-series InSAR analysis tool widely used by the InSAR community. My team uses it in their research, so I can confirm its usability.

Reviewer #1 (Remarks to the Author):

Dear author team,
I want to thank and congratulate you for this careful review. I have nothing to add to my previous comments and look forward to see this work published.
With warm regards,
Sabrina Metzger

Thank you, Sabrina. We are grateful for your comments which helped us significantly improved the manuscript.

Reviewer #1 (Remarks on code availability):

All three software links provided in the data availability section are correct and useful. The link to the data (rate and strain maps in high resolution) is missing. Please make sure to provide your valuable maps to the public.

Thank you for asking. We have now added the DOI (10.5281/zenodo.20293600) to the acknowledgement section.

Reviewer #2 (Remarks to the Author):

This manuscript presents a comprehensive geodetic analysis of deformation across the Tian Shan, integrating large-scale Sentinel-1 InSAR and GNSS data. The identification of a NE-trending, range-scale distributed shear zone and its linkage to the rotation of the Tarim Basin represents a potentially important contribution to understanding intracontinental deformation. The authors have responded carefully to the previous comments, and the manuscript has been significantly improved in clarity, structure, and geodynamic context. I believe it is now suitable for publication after minor revision.

Thank you for your positive comments and your suggested further changes which improved the clarity the uncertainty levels of our assertions in the manuscripts.

A few points could still be clarified to further strengthen the manuscript. First, the interpretation of a "nascent shear zone" is now better justified, but remains somewhat inferential. It would be helpful to more explicitly state what observational criteria uniquely support an incipient shear zone (as opposed to long-lived distributed deformation).

Thank you for the suggestion. We added text to explicitly state that it is the rotation of the Tarim Basin that made us believe this is an incipient shear zone. We proposed how it should develop in the future with further rotation of the Tarim and how the distributed shear zone currently in western Tian Shan might demise.

Lines 783-806:

"As a result, the northeastern end of this cryptic shear zone could develop into a through-going left-lateral strike-slip fault pushing against the crystalline Kazakh Platform in a pattern similar to how the Altyn Tagh Fault currently is established against the rigid southern margin of the Tarim Basin [95]. As the deeply rooted rigid Tarim Basin continues to rotate around its Euler pole located to the southeast of itself at a rate of 0.6–0.7◦/Myr [36, 96], it will push into the weaker Tian Shan crust. This will leave behind a growing boundary of rheological contrast along the southwest portion of the shear zone between the weak Tian Shan material and the strong Tarim Basin in a similar fashion as how the Chaman Fault is juxaposed against the Indian Plate (Fig. 1a). The two ends of the shear zone might not be perfectly aligned, in which case the broad zone of distributed deformation in Western-Central Tian Shan might first deform into a shape similar to today's Pamir. However, as long as India maintains its push, it is plausible that a through-going shear zone will eventually cut through the Tian Shan along the north-east diagonal, like how the Sarez-Karakul Fault is shearing apart the Western and Eastern Pamir."

Second, the discussion of the driving mechanism related to Tarim rotation and Euler pole migration is much improved; however, it would benefit from a clearer statement of the level of constraint (inferred vs. demonstrated) and a brief acknowledgment of uncertainties in the proposed mechanism within the broader India–Eurasia collision framework.

Thank you for the suggestion. We have made clear that our proposed tectonic regime change due to the India-Tarim collision was based on the condition that Tarim's Euler Pole did change place. Further

palaeomagnetic evidence needs to be collected to test this hypothesis, and if true, we believe there is a developing plate-scale share zone. We've stated that we have demonstrated that wedge-style deformation south of the cryptic shear zone is the dominating lengthening mechanism of the Tian Shan at the present day.

Lines 1294-1302:

"The bookshelf-style deformation in the Kazakh Platform was likely more active when Tarim's Euler Pole was near Hami, if Tarim's Euler Pole was indeed centered relatively further north at an earlier stage of the Indo-Eurasian Collision. What is demonstrated in this study is that the wedge-style deformation in the southeast half of the Tian Shan is a more dominant mechanism accommodating Tian Shan's lateral extension at the present day, with Tarim's Euler Pole in the Qilian Shan."

Line 1377:

"We infer that the cryptic nature of the nascent shear zone is due to a southward shift of Tarim's Euler Pole due to the collision between the Indian lithospheric"

Lines 1384-1390:

"Testing this hypothesis requires new palaeomagnetic vertical axes rotation time series data along the cryptic shear zone. If the above hypothesis were true, we suggest that there is a developing extension on the western boundary of the India Eurasia collision zone propagating through the mountain range of the Tian Shan."

Third, the manuscript would benefit from a brief clarification of how these signals are distinguished from tectonic deformation in practice (e.g., spatial scales or filtering considerations), to reassure readers of the robustness of the tectonic interpretations.

Thanks for the suggestion. We have added the following text to stress that one should still use the full-resolution (unfiltered) east and vertical velocities to make tectonic interpretations on single faults, and how the features indicative of the shear zone despite the strong filtering that we applied suggests our interpretation of the cryptic shear zone is robust.

Lines 1363-1370:

"Our study shows that large-scale InSAR velocities can be used to infer deeper processes in addition to localised strain on single faults. Whilst ideally it is best to model deformation across single faults from the unfiltered velocities, the robustness of the northeast-trending diagonal despite strong median filtering clearly delineates a boundary in the regional stress field."

These are relatively minor clarifications and do not require additional analyses.

Comments on manuscript NCOMMS-25-70243 "Extension of Tian Shan along a nascent shear zone" by Ou et al.

Summary

Ou et al. present the first InSAR-derived surface deformation maps of the full Tian Shan mountain range. Using a dense GNSS velocity field compiled from various sources the authors projected 90 individual InSAR rate maps into a European-fixed reference frame. They found a broad shear zone along a northeastern axis, suggesting that the Tian Shan is extending along the strike of that shear zone, which they explain with the northward indentation of the Tarim basin. These findings are backed up with published seismic observations from shear wave splitting and earthquake focal mechanisms. In addition they show examples of localized fault activity, as well as climatic and anthropogenic processes related to ice loss, respectively, water pumping as it has already been observed elsewhere using the same methods.

The author group unites high experience in creating large-scale InSAR products from other orogens and they are constantly refining their strategy of InSAR data processing and time-series analysis to improve the signal-to-noise ratio and produce highly informative surface deformation rate maps, so I have nothing to criticize on the methods. The conclusions drawn from their observations are reasonable. The language is clear and concise, the figures are informative, if somewhat dense or small.

I therefore have only minor questions and suggestions to improve the clarity of the manuscript, see below. Not all my suggestions must be considered, but I think the first two key points in particular would increase the impact of the work for tectonic experts if acknowledged, as they will help to better understand (1) the tectonic evolution of the Tian Shan over time and (2) how large strike-slip systems worldwide might form.

I hope the authors find my comments useful.

Sabrina Metzger, 23.10.2025

Key points

- **The role of Talas Fergana fault (and others)** You introduce the (role and presence of) NW-trending large fault systems at the northwestern margin of the Tian Shan (Talas Fergana fault, AkF, DzF, ChF) very late in the text and I did not understand how you explain their unfavorable geometry w.r.t. the kinematics of the large shear zone.
  Along those lines I suggest to better introduce and later discuss the temporal evolution of the Tian Shan. Are there paleomagnetic records or other geologic data explaining how/when the Tian Shan subranges and dextral large faults systems have been built?
- **Nascent shear zone** You state that we observe here the “birth” of a shear zone. If true, do we find similarities (in topography) in well-developed other shear zone world wide or also in lab experiments? Do we see remnants of (rotated) ranges on both sides of a large strike-slip fault system, e.g. North Anatolian fault (that grew westward), East Anatolian fault, Altyn Tagh, or maybe Southern Alpine fault, San Andreas fault etc. . Also, is there evidence in the Tian Shan that the wedge-shaped faults maybe rotate over time to (in the geologic future) then more align with the strike of the broad shear zone?

- **Chaman-Karakul-Tien Shan shear zone** I my opinion it is a simplification to state that Chaman-Sarez-Karakul-Tian-Shan form one single shear zone, as they are clearly offset, kinematically and structurally, the reason of which could be discussed. My explanation would be the geometry of the Indian plate, but maybe there is another reason?
- **GNSS/InSAR data independence** I very much enjoy large-scale InSAR rate maps and how they present surface deformation. But the text should clearly state that the long-wavelength InSAR rates are mostly (purely?) constrained by the excellent GNSS velocity field of the region. This also touches the discussion whether the community should invest on cheap GNSS coverage or InSAR. It would be great to learn more about your experiences on that matter.
- **More references** If the format allows I would include more references in the discussion. Your data also shows mass loss signal and permafrost melt but you do not go much into detail, for example, guiding the reader to further works that explain how these processes could be modeled.

Suggestions for Figures

Figure 1 Suggestions for better distinguishing features in this dense plot: In a) reduce the contrast in the background (using weak colors or transparency). You could also thin out the high number of GNSS and do not show the rate uncertainties to make them easier to identify and be able to enlarge their size somewhat. In b) you could remove the gray track lines and make the arrows a bit thinner. Instead of using shaded background it might be lighter (but as informative) if you use a minimized topographic color map (1000+m). Subfigures c-f could be moved to the supporting material.

Figure 2 The line width of the colorbar is unnecessarily thick. Check whether all subfigures are needed or if their number could be reduced and instead increase the size of individual subfigures for readability.

Figure 4 In the text the subfigures are not discussed in order. For clarification it would be helpful to use the same color coding in d) as in b) and c) , e. g., color the Tarim block lilac and use the same name (Tarim Basin, or actually block might be better?). Also it is unclear in b) what you mean by the Tian Shan "mirroring" the Western Tibetan Plateau (L355), maybe you could work with colors as well? I also find the Euler pole in b) confusing as it should point into the plane I would say and you could use curved red arrows in b) to d) as you do in a) to represent the block rotation of Tarim. Would it be possible to simplify b) somewhat?

Figure 5 You mention some triangles but I cannot find them (subfigure e and f, I believe).

Detailed suggestions to improve clarity and completeness

Main Text

L63ff: When describing geographic locations I suggest to refer to Figure 1 and make sure that text and figure align. For example, the definition of "Kyrgyz Tian Shan" in line 66 is unclear as no political boundaries are plotted in Figure 1. You could mention maybe that South Tian Shan is more attached to Pamir, while the major part of Tian Shan is located between the basins or so, which is

also why it accommodates more shortening? (I just see that you excluded the South Tian Shan from the analysis, maybe it is worth mentioning.) Later you use southwestern, western and eastern Tian Shan, I think you clearly should mark those regions somewhere.

L73: Can you provide a geologic time to the extension activity?

L96: Can this paragraph be extended with more than just saying that you will present data, e. g., what products you will derive and how it helps to solve the problems raised in the paragraph above?

L113: Instead of just mentioning the rate difference you could directly describe to which direction the east rates increase, which would use same space, but is more informative.

L73/111/246/ff: You mention extension in the Tian Shan several times but it is not always clear along which direction you mean. Since the key message is about this extension accommodated on a yet unidentified NE-shear zone, it would help to clarify throughout the text, of which type of extension you speak. Probably the extension mentioned in L73 is a different one?

L173: one ']' to much

L175-177: Are these average numbers per frame? Per Pixel? I am confused because you mention earlier that you processed over 100,000 ifgs in total. Should it be (more precise): "before/after quality control" rather than time-series?

L261: Figure 4d is referenced before the other subfigures of Figure 4.

L315: This could be material to mention at the end of the introduction?

L399/568: Is “indented” a widely used term in the community for the kinematics or the shape of the Tarim basin? For me, an indenter is something more pointy.

L400-409 and 418 and 427 and 430: The block (or arc) rotation of the Tarim basin should be better visualized in the figures, e.g. Figure 4b-d and maybe also in Figure 1, see the white arrows in this unpublished figure (cf. Enrico Serpelloni) of the Adria plate as inspiration:

[REDACTED]

In addition, it is sometimes hard to follow what the azimuth numbers indicate (an angle between what and what?). Maybe you can add “XX°N” if they measure an azimuth clockwise from North? Angles on a map would help.

L420-427: These two statements are somewhat contradicting: If the faults are nearly perpendicular the expected kinematics would be pure thrusting but then you say that the rates are partitioned?

(L443-455: Could be partially explained in the method section if you need more space.)

L455-460: But they also nicely align with the individual mountain ranges of the Tien Shan, not only the band at depth.

L468-473: But there is also a significant offset between these three structures, maybe related to the Indian indtenter? See Kufner et al. 2018a that propose a correlation of the Chaman-Badakhshan axis linked to marginal India, where as the Sarez-Karakul is linked to the cratonic India (https://doi.org/10.1029/2017TC004812, Figure 9) and Bloch et al. 2021 that completed the picture of the Eastern Pamir/Tarim (but is harder to interpret in how it might impact the NE-shear zone you detect, https://doi.org/10.1029/2021GL095413, Figure 4).

L481ff: Kufner et al. 2018B (https://doi.org/10.1029/2018JB015926) also present shear wave splitting data that does not cover new regions, but also shows some flow direction parallel to the TFF like Cherie et al. 2016.

L524-547: After the statements in this paragraph it would be interesting to discuss whether other large strike-slip fault systems went through similar stages before, for example, whether this is (still) reflected in the topography (Northern/Eastern Anatolian fault, northern part of the New Zealand Alpine fault, San Andreas fault?), and whether this is also reflected in the eastern Himalayan syntaxis?
There is also numerical modeling done of how strike-slip faults are born (early analog: https://doi.org/10.1029/JB093iB12p15085

L599: "many rigid blocks in Tian Shan" is this just a descriptive term or a proven statement (like micro/blocks)?

L600: To facilitate an easy comparison of your conceptual model in 4c to the geographic features on a DEM it would be helpful if you repeat all features in Figure 4c also in Figure 1a and 3a.
In your conceptual model the role of TFF and other faults with similar strike along the northwestern margin of the Tian Shan (DhF, AkF, DzF, ChF) remains unclear. The TFF is an impressive structure, but is completely misoriented to your stress field. It also is right-stepping which does not agree to the large sense of motion indicated in Figre 3a. Is it inherited from a previous stress field and how could that have looked like?

L615: Figure 4b is discussed after 4c, consider rearranging the subfigures in this figure.

L655: Has this lengthening mechanism of the southeast half of the Tian Shan been introduced/discussed earlier in the text? Why does it only affect the Southeastern part, and not the whole area covered by the newly found shear zone?

L665-677: Can these important statements be backed up with more references, e.g. works from tomography? Is it clear that the Junggar Basin is significantly weaker than the Kazakh platform?

L719/720: Can you provide a reference to a similar finding that includes a modeling attempt?

L729: I cannot identify those red triangles in Figure 5e

L746: A figure reference might help here to sort out the geographic features or mark those in 5f? Actually Kalpin is only labeled in figure 4c, Kuqa nowhere? It is a bit hard to follow you until L766.

L768: Similar to the ice mass loss, it would be good to refer to a case study where this has been effectively modeled, e.g. Californian Central Valley (https://doi.org/10.1029/2019JB018490, but maybe not a key publication)

L812: Can you explain the meaning of the PZI, what is 0 what is 1?

L815: I cannot find the blue triangles. The gray shading in the profiles is not explained.

L829: Similar to above this statement could be backed up with some key literature where one can learn about the modeling strategy of such features.

L875: Since this is the discussion section it would be nice to better embed your findings into existing literature. In the result section you do this by (I think) reporting on similar findings. Maybe you could elaborate a bit on the process here (compaction due to pore pressure, or creep, or common compaction). For example I still wonder whether one can discriminate between signals caused by loss of permafrost and signals caused by simple gravitational compaction of unconsolidated sediments. Is the signal not also observed at low-latitude regions without permafrost but gravelly slopes?

L894: Is there any literature that could be suggested for further reading?

L897-905: While I find this paragraph somewhat generic I suggest to better point out how large InSAR rate maps can be used to discuss deeper processes instead of focusing on single faults, e.g. by comparing to shear wave splitting data and the like which opens new opportunities. (However this should be discussed in the context of how much InSAR there is left in the long-wavelength, or how much it is dominated by GNSS – where critics could ask why we need InSAR…)
A second line of thought I suggest to think about: Do all large strike-slip systems evolve over time like we see now in the Tian Shan, or is this coincidence and mostly related to a change in the stress field over time? Would you find similar old topography along other large strike-systems world wide?

L929: Provide a reference to LiCS system.

L936: I would exclude Fig 1c-f from the main text and put it to the supporting material.

L972: mention that SNAPHU is a phase unwrapping algorithm

L955ff: See my comment to the figure caption of figure 1: I do not understand whether this is the average number for all tiles or something else?

References: The references sometimes lack capitalization, which (in latex) can be solved by using double brackets (title = {{…}})

Supporting Text

L35-39: Fix reference error

The references are missing?
